# High energy triplet-state manipulation via temperature-responsive twisted hetero-annulation systems

**DOI:** 10.1038/s41467-026-73715-8

**Published:** 2026-05-25

**Authors:** Guigui Ye, Yan Gao, Wentao Yuan, Juqing Gu, Jiaqiang Wang, Yujie Yang, Qianqian Li, Zhen Li

**Affiliations:** 1 Hubei Key Lab on Organic and Polymeric Opto-Electronic Materials, Department of Chemistry, Wuhan University, Wuhan, China; 2 School of Sports Medicine, Wuhan Sports University, Wuhan, China

**Keywords:** Excited states, Organic molecules in materials science, Optical materials

## Abstract

Precisely controlling excited-state transitions is vital for advanced technologies, particularly for sensitive, long-lived triplet states. Unlike traditional research focused on the lowest-energy excited triplet state (T_1_), this work focuses on the high-energy triplet state (T_n_), which presents significant challenges due to its short-lived and elusive nature. By implementing an annulation strategy to tailor the size, geometry, and electronic properties of the π-conjugated systems, we achieve precise control over two distinct T_n_-mediated pathways, T_n_ → S_1_ → S_0_ and T_n_ → T_1_ → S_0_ transitions, using temperature as an external trigger. This approach enables temperature-modulated blue-to-red afterglow, characterized by an exceptionally high energy gap of up to 0.76 eV between the delayed fluorescence and phosphorescence. It offers an elegant solution to resolving the key challenge in T_n_ manipulation, providing a blueprint for developing next-generation responsive organic semiconductors with tailored excited-state behavior.

## Abstract (web summary)

Control of excited state transitions is important for a range of applications, but can be challenging. Here, the authors report the tailoring of size, geometry and electronic properties of conjugated systems to control high-energy and lowest-energy triplet-mediated pathways.

## Introduction

**Fig. 1:**
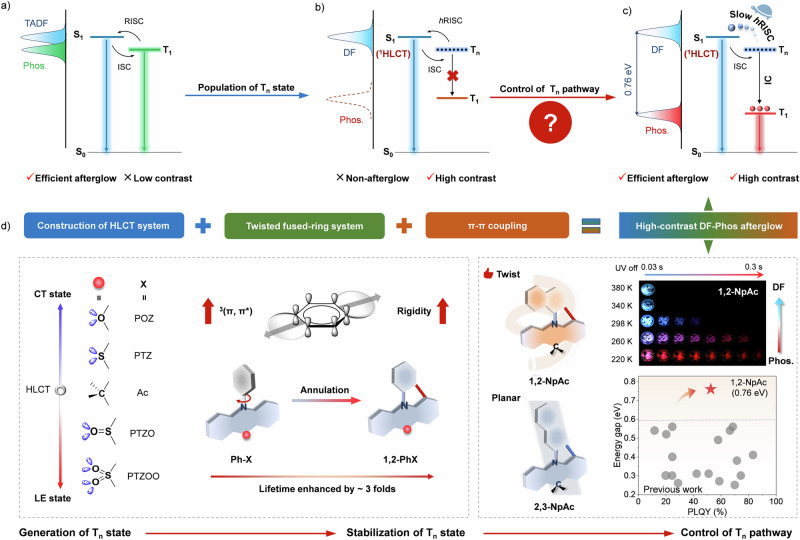
Schematic illustration of modulating high-energy triplet state transition pathways. **a** Schematic illustration of phosphorescence and TADF emission related to the transitions from T_1_ states. **b** Schematic illustration of DF emission from high-level triplet states (T_n_) by *h*RISC processes. **c** Schematic illustration of both phosphorescence and DF emission related to the transitions from T_n_ states. **d** The molecular structures of extended π-conjugation systems through annulation strategy, and the adjustment of electronic property by the incorporation of various nitrogen-containing heterocyclic systems (POZ, PTZ, Ac, PTZO, and PTZOO); the corresponding afterglow photographs of 1,2-NpAc across the temperature range of 220–380 K, in which the afterglow color changes from red to blue by the conversion of phosphorescence and DF emission; the recent progress in temperature-modulated afterglow (observable by the naked eyes) through the conversion between DF and phosphorescence in single-component systems (*y*-axis corresponds to the energy gap between DF and phosphorescence, and *x*-axis corresponds to the PLQY, the corresponding data was shown in Table S2).

Excited states are the transient, high-energy engines that drive the functionality of organic opto-electronic materials^1–3^, including light-emitting diodes^4–6^, solar cells^7,8^, and sensors^9,10^. Luminescence, which serves as the visible signature of an excited state returning to the ground state (S_0_), can reveal the nature and pathway of excited states through its timescale and temperature-dependent properties^11^. Regarding the afterglow with long-lifetime (ms∼s) emission, it is typically associated with excited triplet states in spin-forbidden processes, such as intersystem crossing (ISC), reverse intersystem crossing (RISC), and transitions from excited triplet states to the S_0_ state^12–14^. Actually, phosphorescence from triplet-state decay and delayed fluorescence (DF) via RISC with afterglow emission can be achieved by regulating the pathways of excited triplet states^15^. According to Kasha’s Rule^16^, the characterization of the lowest-energy excited triplet state (T_1_) plays a crucial role in the wavelengths, brightness, and lifetimes of phosphorescence emission. Through the dedicated efforts of researchers, effective strategies have been developed to promote T_1_ generation via facilitated ISC, extend T_1_ lifetime through suppressed nonradiative decay, and modulate T_1_ energy levels by tailoring electronic properties^17–20^. Various afterglow materials with colorful emissions and prolonged duration have been developed by the incorporation of polymers as rigid matrices^21,22^, crystal engineering^23,24^, the employment of heteroatoms and halogens^25,26^, etc. For the DF-type afterglow, the RISC process is essential, and largely determined by the energy gap (Δ*E*_ST_) between the excited singlet (S_1_) and triplet (T_1_) states (Fig. 1a), further confirming the key role of T_1_ states^27–30^. Until now, the transition pathways from T_1_ states, including T_1_ → S_1_ and T_1_ → S_0_, can be well-managed by the modulation of molecular structures and aggregation behaviors, resulting in the high utilization of T_1_ states with improved afterglow performance.

In contrast, T_n_ states (*n* ≥ 2, higher-energy triplet states, e.g., T_2_, T_3_) have historically been overlooked, as they are short-lived, high-energy, and challenging to characterize. However, recent advances in spectroscopy and theoretical modeling have enabled systematic investigation of T_n_ states, revealing their value that complements and extends the limitations of T_1_-focused research^31–33^. For instance, high-level RISC (*h*RISC) from T_n_ to S_1_ has been explored as a strategy to address the trade-off between large singlet–triplet energy gaps (Δ*E*_ST_) and efficient RISC processes (Fig. 1b). This approach is primarily realized through the construction of hybridized local and charge-transfer (HLCT) excited states, which integrate both local excitation (LE) and charge-transfer (CT) characteristics^34,35^. By employing this strategy, a wide range of Δ*E*_ST_ values (0.03–0.93 eV) has been achieved, accompanied by DF emission (Fig. S1 and Table S1). Generally, the *h*RISC process from short-lived T_n_ states demonstrated fast rates, offering an efficient way to address the critical “efficiency roll-off” problem in organic light-emitting diodes (OLEDs)^36^. Meanwhile, the ultrafast dynamics inherent to the T_n_ state render it both transient and difficult to capture, control, or modulate in most cases, consequently limiting the breadth of research in this area.

Herein, we report an efficient strategy for manipulating T_n_ states by integrating annulation strategies into diverse heterocyclic frameworks. Through synergistic regulation of molecular conformations and aggregation modes, we achieve precise, temperature-controlled modulation of T_n_-mediated transition pathways (Fig. 1c and Table S2), specifically the T_n_ →  T_1_ → S_0_ and T_n_ → S_1_ → S_0_ routes. Notably, the fused-ring conjugated systems exhibit HLCT excited states with large singlet-triplet energy gaps, which facilitate potential T_n_-mediated transitions^37,38^. Moreover, the rigid molecular skeletons, combined with strong intermolecular interactions in the aggregated state, effectively restrict molecular motions with nonradiative internal conversion (IC) from T_n_ states. In addition, enhanced intermolecular electronic coupling in the crystalline state amplifies spin-orbit coupling (SOC) between singlet and triplet states. Collectively, these features enable the involvement of T_n_ states in the *h*RISC process (T_n_ → S_1_) while preserving radiative decay from T_n_ → T_1_ → S_0_. As a result, DF and phosphorescence coexist, giving rise to a high-contrast, temperature-dependent afterglow that transitions from blue to red and is clearly visible to the naked eye. The large energy gap up to 0.76 eV between DF and phosphorescence further confirms the efficient regulation of high-lying triplet states (Fig. 1d). This work not only deepens the understanding of excited-state dynamics but also harnesses the full potential of excited states intelligently and effectively, laying the groundwork for predicting and designing a series of functional materials with tailored excited-state behaviors.

## Results

### Molecular design and characterization

**Fig. 2:**
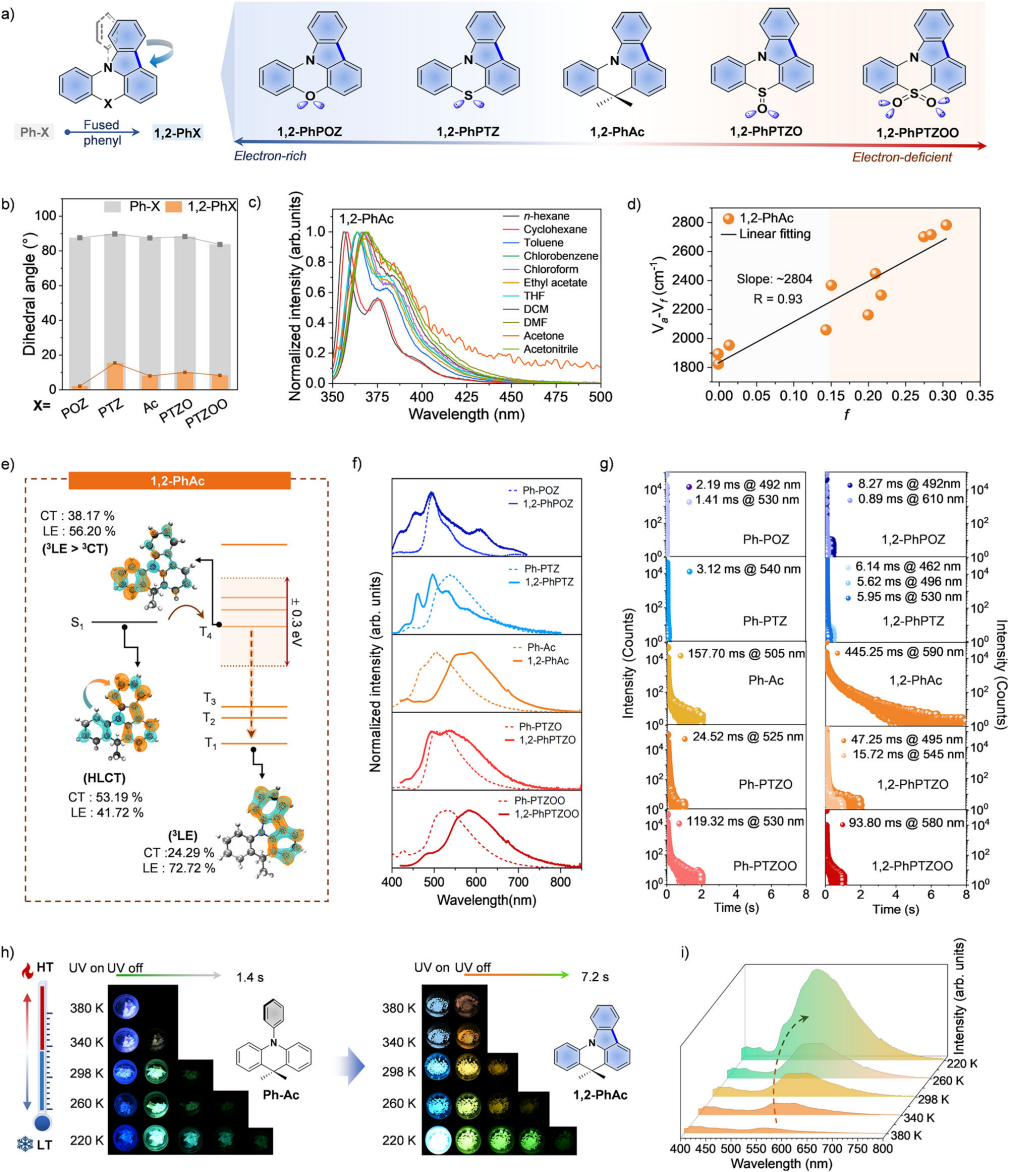
Characterization of phenyl-based fused-ring systems. **a** Molecular structure of phenyl-based fused-ring systems with various electronic properties. **b** The change of molecular conformation between Ph-X and 1,2-PhX (X = POZ, PTZ, Ac, PTZO, PTZOO), with the variations in dihedral angles between phenyl (Ph) moieties and X core. **c** The normalized photoluminescence spectra of 1,2-PhAc (10 μM) in different solutions at room temperature. **d** The solvatochromic Lippert–Mataga models of 1,2-PhAc, where the *x*-axis denotes solvent orientational polarizability, and the *y*-axis represents the Stokes shift. **e** The energy levels and isosurface plots (isovalue = 0.0020) of electron–hole distributions in the S_1_, T_n_, and T_1_ states of 1,2-PhAc as isolated states. **f** The delayed emission spectra (1 ms delay) of Ph-X and 1,2-PhX crystals. **g** The delayed emission decays of Ph-X and 1,2-PhX crystals. **h** The afterglow photographs of Ph-Ac and 1,2-PhAc crystals with the temperature ranging from 220 to 380 K. LT low temperature, HT high temperature. **i** The delayed emission spectra (1 ms delay) of 1,2-PhAc crystals with the temperature ranging from 220 to 380 K.

The commonly used functional moieties with room temperature phosphorescence (RTP) property^39^, including phenoxazine (POZ), phenothiazine (PTZ), 9,9-dimethyl-9,10-dihydroacridine (Ac), 10H-phenothiazine-5-oxide (PTZO) or phenothiazine-5,5-dioxide (PTZOO) moiety, bearing the same *N* atom and different heteroatoms (O, S, S=O, O=S=O, etc.) with varied electronic properties have been employed as conjugation cores (defined as X). The introduction of different heteroatoms alters the orbital composition of the excited states, shifting their n → π versus π → π* character and consequently modulating ISC and radiative decay pathways^40,41^. The *N*-phenyl substituents were introduced by *Buchwald-Hartwig* C–N coupling reaction to form Ph-X lumonogens, including Ph-POZ, Ph-PTZ, Ph-Ac, Ph-PTZO, and Ph-PTZOO as the references, and the cyclization process was conducted by one-step C–C and C–N coupling reaction to form the target 1,2-PhX luminogens (1,2-PhPOZ, 1,2-PhPTZ, 1,2-PhAc, 1,2-PhPTZO, and 1,2-PhPTZOO) (Fig. 2a). Furthermore, fused-naphthalene moiety with a larger π-conjugation plane was incorporated by a similar procedure to obtain the target luminogens of 1,2-NpAc and 2,3-NpAc with different fusion sites (Supporting Information, Figs. S2and S3). These luminogens were well characterized by ^1^H and ^13^C NMR spectroscopy, mass spectrometry, high-performance liquid chromatography (HPLC), and elemental analysis (Figs. S4–S18).

### Photophysical properties of phenyl-based fused-ring systems

Compared to the *N*-phenyl-substituted analogues (Ph-X series), the fused-phenyl derivatives (1,2-PhX series) exhibited red-shifted absorption spectra in THF solution (10 µM, Table S3 and Fig. S19), accompanied by reduced energy gaps between the highest occupied molecular orbitals (HOMOs) and the lowest unoccupied molecular orbitals (LUMOs) energy levels (Figs. S20 and S21). It was mainly due to conformation transformation from twist to planar ones by the annulation strategy, as proved by largely decreased dihedral angles between the *N*‑phenyl rings (Ph) and X moieties, from 83.72° to 89.72° of Ph-X series to 2.07°–15.43° of 1,2-PhX ones (Figs. 2b and S22). Accordingly, the photoluminescence quantum yields (PLQYs) generally exhibit an increasing tendency from the Ph-X series to the 1,2-PhX ones (Table S3). Notably, pronounced enhancements are observed for 1,2-PhPOZ and 1,2-PhAc, whereas the other derivatives show modest improvements, mainly due to the suppression of molecular motions by the locked rings and corresponding conjugated effect. With the introduction of various solvents with increased polarities from *n*-hexane to acetonitrile, the emission spectra of the 1,2-PhX series undergo gradual red shifts (10–20 nm) and progressive broadening, evolving from well-resolved vibronic structures to featureless bands (Figs. 2c, d, and S23). The excited-state dipole moments derived from quantitative analysis, ranging from 4.9 to 14.6 D, confirm the involvement of partial CT character in the excited state (Tables S4 and S5). Furthermore, theoretical calculations reveal that the S_1_ state of the 1,2‑PhX series involves a balanced contribution from CT (47.76–58.64%) and LE (39.29–50.84%) components^42–44^, resulting in HLCT excited states (Figs. 2e and S24). With the adjustment of luminescence cores by the variations in C10-positions from electron-donating units (O and S) to the electron-withdrawing ones (S=O and O=S=O), the corresponding maximum emission wavelengths blue-shifted from 405 nm (1,2-PhPOZ) and 415 nm (1,2-PhPTZ), to 384 nm (1,2-PhAc), 374 nm (1,2-PhPTZO), and 375 nm (1,2-PhPTZOO) in THF solution, respectively, similar to the trend of the maximum absorption wavelength under the same conditions, indicating the adjustable intramolecular charge transfer through the whole conjugation skeletons (Table S3).

At 77 K, the obvious blue and green afterglow can be observed as phosphorescence emission in these solutions. 1,2-PhAc, 1,2-PhPTZO, and 1,2-PhPTZOO demonstrated the blue-shifted emission with wavelengths located at about 460 nm (Fig. S19), compared to those of 1,2-PhPOZ (470 nm) and 1,2-PhPTZ (515 nm). The 1,2-PhX series exhibited much longer phosphorescence lifetimes than those of Ph-X analogues (Fig. S25 and Table S6), for instance, the phosphorescence lifetime of Ph-Ac was 3.29 s at 425 nm, while it increased to 5.76 s at 420 nm for 1,2-PhAc. It may be attributed to the increased ratios of (π, π*) character in the T_1_ excited states by the extended π-conjugation system with annulation strategy, and confirmed by the increased^3^ (π, π*) ratios from 62 to 76% in the Ph–X analogues to 66–99% in the 1,2-PhX series from theory calculations (Fig. S26). The decreased or similar SOC values observed upon annulation indicate that the enhanced (π, π*) character contributes to the prolonged phosphorescence lifetime (Figs. S27–S31 and Tables S7–S11). Moreover, the rigid structures of fused rings can effectively suppress the possible molecular motions as nonradiative transitions, thereby benefiting the persistent phosphorescence emission. Among these, 1,2-PhAc exhibits the highest (π, π*) contribution (99%) in the T_1_ state, leading to the longest phosphorescence lifetime up to 5.76 s at 77 K. Additionally, the T_1_ state of 1,2-PhAc exhibits a high percentage of LE character (72.72%) (Fig. 2e), the pronounced difference in excited-state characteristics of S_1_ and T_1_ state results in the large Δ*E*_ST_ (0.46 eV) as isolated state, calculated by the PL and phosphorescene spectra of 1,2-PhAc in THF solution at 77 K (Fig. S32).

When these luminogens aggregated into crystalline states at room temperature, only partial Ph-X and 1,2-PhX series (X =  Ac, PTZO, and PTZOO) exhibited afterglow visible to the naked eye, with duration times of 0.1–2.0 s (Fig. S33). The afterglow colors changed from green (Ph-Ac, Ph-PTZO, and Ph-PTZOO crystals) to orange (1,2-PhAc, 1,2-PhPTZO, and 1,2-PhPTZOO crystals) by annulation strategy, accompanied by the red-shifted delayed emission wavelengths (1 ms delay) from 505 nm (Ph-Ac) to 590 nm (1,2-PhAc), from 525 nm (Ph-PTZO) to 545 nm (1,2-PhPTZO), and from 530 nm (Ph-PTZOO) to 580 nm (1,2-PhPTZOO), respectively (Fig. 2f and Table S3), as well as the red-shifted or similar absorption and PL spectra (Fig. S34). The trend of emission properties at aggregated states was similar to that in solution as isolated states, mainly due to the planarized conformations of the 1,2-PhX series with extended conjugation, together with the more compact molecular packing in the single crystals (Figs. S35–S39 and Tables S12, S13). Among these, the 1,2-PhAc crystal exhibited the longest delayed-emission lifetime of 445.25 ms (Figs. 2g, S40, and Table S14) with a high PLQY of 46.76% (Table S3), which was mainly due to the highest (π, π*) contribution of excited triplet states and the isolated dimeric π–π stacking in the single crystal as optimized aggregated structures.

Moreover, the afterglow property of the 1,2-PhAc crystal demonstrated the temperature-sensitive property. At low temperature (220 K), it exhibited green afterglow with emission wavelength (1 ms delay) located at 545 nm (Figs. S41 and S42), while the afterglow color changed to orange as the temperature gradually increased to 380 K, with the emission wavelength red-shifted to 590 nm (Fig. 2h, i). For comparison, the *N*-phenyl-substituted analog Ph-Ac crystal exhibited the maintained green afterglow under the same conditions (Fig. S43), which was insensitive to temperature changes. Subsequently, according to the monotonic increase in emission intensity of the 1,2-PhAc crystal with decreasing temperature from 380 to 220 K, and the exponential decay observed in time-resolved emission spectroscopy (TRES) measurements (Fig. S44), the dynamic afterglow of the 1,2-PhAc crystal may be phosphorescence from varied triplet excited states with different energy levels. It can be partially proved by the theory calculation, the IC process of 1,2-PhAc trimer can be suppressed by the enlarged T_n_-T_1_ gap and stronger intermolecular interactions, compared to that of Ph-Ac trimer (Fig. S45 and Table S15).

### Photophysical properties of naphthyl-based fused-ring systems

**Fig. 3:**
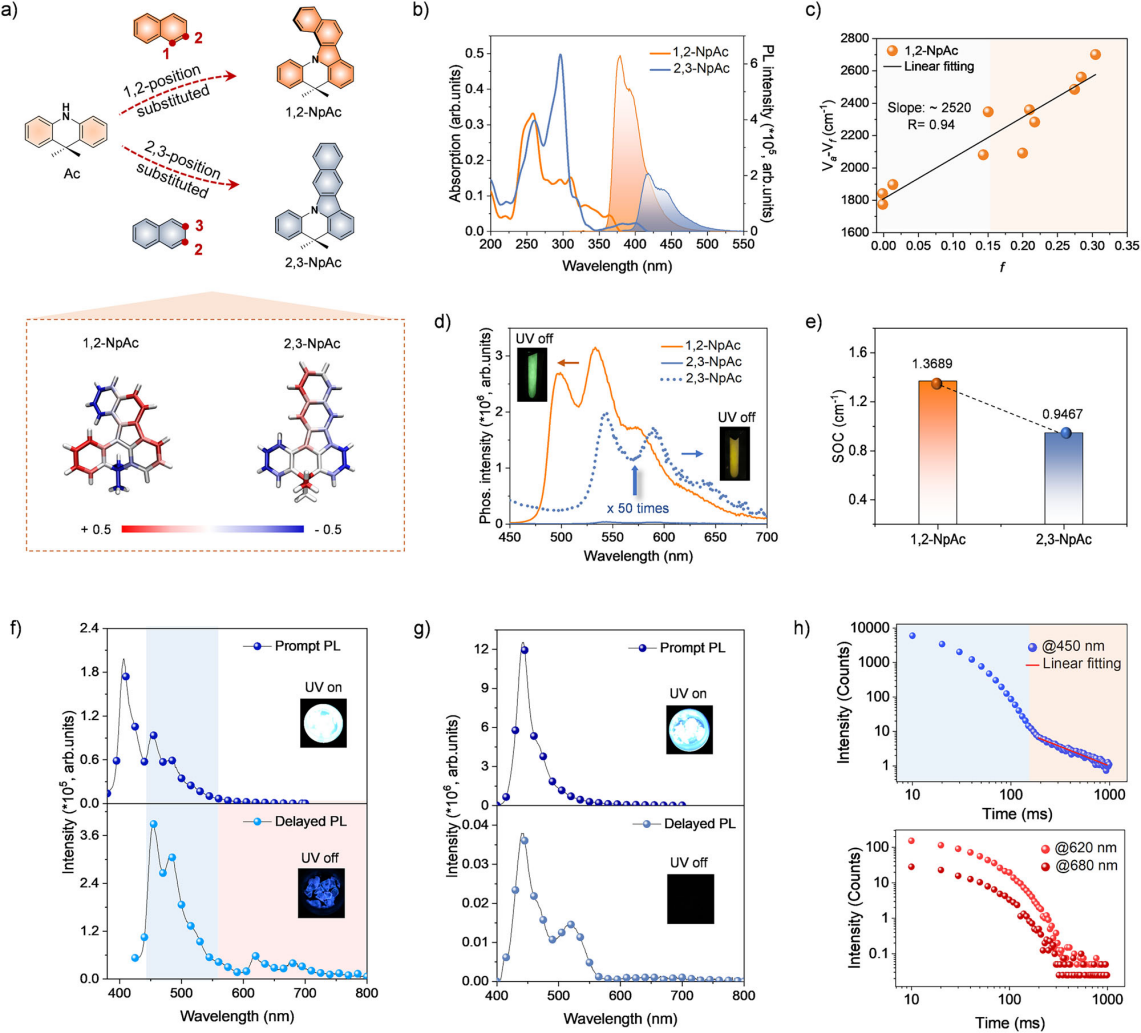
Characterization of naphthyl-based fused-ring systems. **a** Molecular structures of 1,2-NpAc and 2,3-NpAc and the corresponding planarity schematic of 1,2-NpAc and 2,3-NpAc (the redder/bluer color, the larger the distance of the atom below/above the fitting plane). **b** Absorption and photoluminescence spectra of 1,2-NpAc and 2,3-NpAc in THF solution (10 μM) at room temperature. **c** The solvatochromic Lippert–Mataga models of 1,2-NpAc, where the *x*-axis denotes solvent orientational polarizability, and the *y*-axis represents the Stokes shift. **d** Phosphorescence spectra of 1,2-NpAc and 2,3-NpAc in THF solution (10 μM) at 77 K. **e** The summarized SOC values between T_n_ and S_1_ for 1,2-NpAc and 2,3-NpAc in the isolated state (the geometry structure is from single-crystal data). Prompt and delayed (1 ms) photoluminescence (PL) spectra of **f** 1,2-NpAc and **g** 2,3-NpAc crystals, along with the corresponding photographs. **h** Time-resolved emission decay kinetics of 1,2-NpAc crystal from TRES.

Based on the preliminary investigation of the 1,2-PhX series with modulated electronic properties, 1,2-PhAc is identified as a promising afterglow material exhibiting pronounced temperature responsiveness, highlighting the critical role of the Ac moiety and the annulation strategy. Furthermore, naphthyl with the extended π-conjugation, compared to phenyl, was incorporated and fused to Ac moieties at 1,2- and 2,3-position to construct the two isomers of 1,2-NpAc and 2,3-NpAc (Fig. 3a). The more twisted configuration was formed in 1,2-NpAc with the larger dihedral angles between adjacent phenyl moieties (corresponding to θ_1_, θ_2_, and θ_3_, respectively), which were 51.61°, 39.04°, and 9.10° (Fig. S46), compared to 31.87°, 29.67°, and 2.22° in 2,3-NpAc, respectively. Their conformational differences were further assessed via planarity analysis using atomic color mapping, based on the molecular planarity parameter (MPP) and the span of deviation from plane (SDP)^45^. 1,2-NpAc exhibits more intense red and blue regionson the red–white–blue scale, with higher MPP (0.7259) and SDP (3.6470) values, compared to those of 2,3-NpAc (MPP: 0.5221 and SDP: 2.7921), reflecting greater deviations from planarity in 1,2-NpAc. Such conformational differences between the isomers of 1,2-NpAc and 2,3-NpAc led to significant variations in their photophysical properties. As shown in Fig. 3b, 1,2-NpAc exhibited a blue-shifted absorption spectrum, compared to that of 2,3-NpAc in diluted THF solution (10 µM) at room temperature, along with the larger HOMO-LUMO energy gap (Fig. S47). Also, the PL spectrum of 1,2-NpAc blue-shifted compared to that of 2,3-NpAc, with the maximum emission wavelengths of 405 nm (1,2-NpAc) and 450 nm (2,3-NpAc), respectively. Increasing solvent polarity (from *n*-hexane to acetonitrile) resulted in gradual red shifts of 12 nm (1,2-NpAc) and 16 nm (2,3-NpAc), respectively, along with progressive spectral broadening in their PL spectra. These spectra evolved from well-resolved vibronic structures to featureless bands, closely resembling the behavior observed for 1,2-PhAc. The derived excited-state dipole moments, ranging from 8.0 D to 9.9 D for 1,2-NpAc and from 6.8 D to 11.8 D for 2,3-NpAc, further confirm the partial CT character of these excited states (Figs. 3c, S48, and Table S16).

At 77 K, phosphorescence emission can be detected in 1,2-NpAc and 2,3-NpAc solutions with green and yellow emission, accompanied by the maximum phosphorescence emission wavelength of 575 nm (1,2-NpAc) and 640 nm (2,3-NpAc), respectively. As shown in Fig. 3d, 1,2-NpAc demonstrated the much higher phosphorescence intensity and longer lifetimes (2.72 s at 500 nm, 2.71 s at 535 nm, 2.71 s at 575 nm) than those of 2,3-NpAc (1.89 s at 538 nm, 1.60 s at 585 nm, 1.65 s at 640 nm) (Fig. S49 and Table S17). Since the molecular motions as nonradiative transitions have been efficiently suppressed at low temperature (77 K), their difference in afterglow property was mainly related to the varied ISC processes by the modulated molecular configurations. Through the investigation of the possible ISC processes between S_1_ and T_n_ states (S_1_ ± 0.3 eV) by theory calculation (PBE0 functional with def2svp basis)^46^, the sum of SOC constants was calculated to be 1.3689 cm^−1^ (1,2-NpAc) and 0.9467 cm^−1^ (2,3-NpAc), respectively (Figs. 3e, S50, and Table S18). The higher SOC constants of 1,2-NpAc can be attributed to the twisted molecular configuration with reduced molecular orbital symmetry, which promotes orbital mixing and increases electron cloud overlap^47,48^, thereby facilitating the ISC processes to improve the phosphorescence property of 1,2-NpAc as isolated states (Fig. S51). Additionally, the delayed emission spectrum of 1,2-NpAc in epoxy resin at room temperature closely resembles that recorded in dilute THF solution at 77 K, featuring emission peaks at 500, 535, and 575 nm (Fig. S52), respectively. As the temperature increased, the emission wavelengths showed negligible changes, and the corresponding intensities and lifetimes decreased monotonically, consistent with phosphorescence emission (Table S19).

At crystalline states, only 1,2-NpAc demonstrated bright blue afterglow at room temperature, while nearly no afterglow can be observed in 2,3-NpAc (Fig. 3f, g). The corresponding delayed emission spectra (delay 1 ms) of the 1,2-NpAc crystal display dominant emission peaks at 450 and 485 nm, accompanied by weaker emissions at long-wavelengths of 620 and 680 nm, respectively. The mechanisms of these emissions at different wavelengths can be determined by TRES decay curves. The short-wavelength afterglow emissions (at 450 and 485 nm) exhibited an initial exponential decay, followed by the emergence of a linear component, indicating the involvement of multiple excited-state processes (Fig. 3h and S53). In contrast, the long-wavelength emissions (620 and 680 nm) exhibit purely exponential decay, characteristic of a single phosphorescence pathway. These distinct decay behaviors demonstrate that different emission mechanisms govern the short- and long-wavelength regions. Comparison with background signals confirms that the decay traces originate from the sample rather than instrumental noise (Fig. S54).

**Fig. 4:**
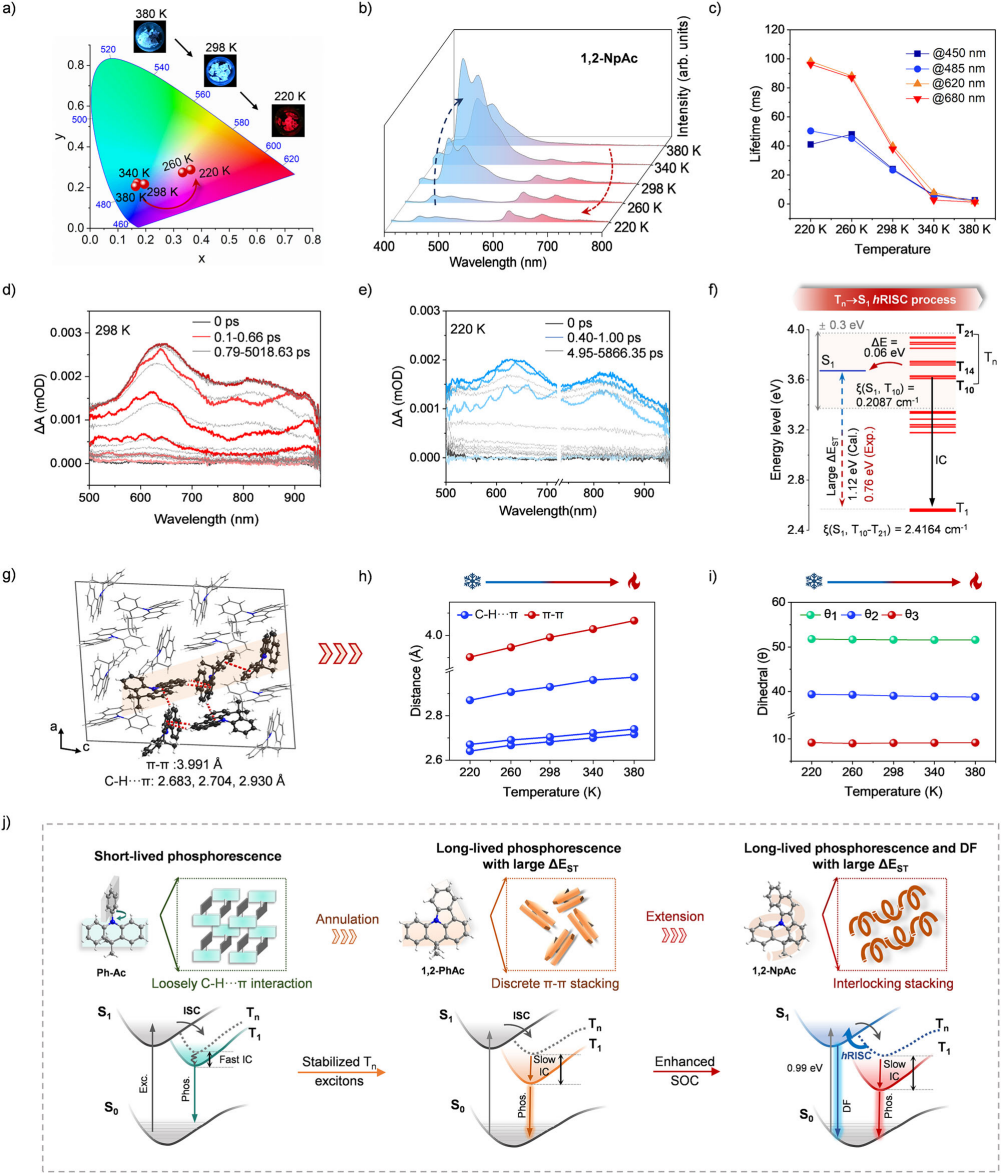
Mechanism of naphthyl-based fused-ring systems. **a** The CIE 1931 chromaticity diagram, **b** delayed emission spectra (1 ms delay) and **c** delayed emission lifetime of 1,2-NpAc crystals across the temperature range of 220–380 K. TA spectroscopy of 1,2-NpAc crystal at **d** 298 K and **e** 220 K, respectively. **f** The energy levels of 1,2-NpAc trimer and corresponding SOC values. **g** The molecular packing and intermolecular interactions in 1,2-NpAc crystals. The changes of **h** intermolecular interactions and **i** molecular conformations in 1,2-NpAc crystal across the temperature range of 220–380 K. **j** Schematic diagram of the optimized molecular structures from Ph-Ac to 1,2-PhAc, then to 1,2-NpAc by annulation strategy, the corresponding molecular packing in Ph-Ac, 1,2-PhAc and 1,2-NpAc crystals, and the afterglow emission changing from weak phosphorescence (Ph-Ac) to bright phosphorescence (1,2-PhAc), then to both bright DF and phosphorescence (1,2-NpAc) with large energy gaps.

Accompanied by the temperatures decreasing from room temperature to 220 K, the blue-to-red afterglow variations have been observed (Fig. 4a), accompanied by CIE coordinates changing from (0.16, 0.20) to (0.36, 0.29). It is mainly due to the different temperature sensitivities of short-wavelength (450 and 485 nm) and long-wavelength (620 and 680 nm). As shown in Fig. 4b, the emission intensity at short wavelengths (450 and 485 nm) increases significantly as the temperature rises from 220 to 340 K, and slightly decreases with further heating to 380 K, suggesting the presence of thermally activated processes. The prompt PL and delayed emission spectra show overlapping emission bands at 450 and 485 nm (Fig. S55), indicating that these features originate from the same emissive singlet state, which is consistent with the characteristics of DF. While the emission intensities at long wavelengths (620 and 680 nm) continuously decreased with the increased temperature, confirming the phosphorescence emission. Temperature-dependent lifetime measurements also provide strong evidence for the distinct mechanisms underlying the short- and long-wavelength delayed emissions in 1,2-NpAc. The data show that as the temperature rises from 220 to 380 K, the long-wavelength emission (620 and 680 nm) undergoes severe quenching, with its lifetime plummeting from 98.22 and 96.22 ms to just 1.43 and 1.21 ms. This behavior is also characteristic of phosphorescence, which is highly susceptible to thermally activated nonradiative decays. In contrast, the short-wavelength afterglow (450 and 480 nm) exhibits weaker temperature dependence, with lifetimes decreasing modestly from 41.04 and 50.34 ms to 2.64 and 1.86 ms, respectively (Figs. 4c, S56, and Table S20). This behavior is consistent with the possible up-conversion pathways, where thermally activated RISC competes with nonradiative decay, partially offsetting the expected lifetime shortening.

Thus, high-contrast dynamic afterglow is achieved through the distinct temperature-sensitive properties of phosphorescence and DF. Phosphorescence dominates at low temperatures (220–260 K), whereas DF prevails at room temperature and above (298–380 K). Accordingly, the dominated emission peaks exhibit a temperature-dependent shift of up to 170 nm, from 450 nm at 298 K to 620 nm at 220 K, corresponding to an energy gap of 0.76 eV. This represents the largest spectral separation between DF and phosphorescence reported to date in temperature-modulated afterglow materials.

Systematic photophysical investigations in both isolated and crystalline states reveal that the high-contrast, temperature-dependent afterglow color change of 1,2-NpAc arises from the combined effects of intrinsic excited-state characteristics and electronic coupling in the crystalline state. In the isolated state, 1,2-NpAc exhibits only phosphorescence. Its pronounced (π, π*) character by naphthyl-fused conjugation leads to a relatively large singlet–triplet energy gap (~0.38 eV) (Fig. S57), while the twisted π-conjugated structure enhances SOC, enabling potential T_n_-mediated transitions from S_1_ → T_n_ → T_1_ → S_0_. This establishes an intrinsic basis for efficient triplet exciton utilization.

Upon aggregation into the crystalline state, the 1,2-NpAc crystal exhibits dual-emission afterglow consisting of both DF and phosphorescence. Quantitatively, the energy gap between DF and phosphorescence is measured to be 0.76 eV, substantially larger than 0.3 eV. This observation suggests that efficient RISC is unlikely to occur directly between T_1_ and S_1_ *via* the conventional pathway. Instead, these processes are likely mediated by T_n_ states, which serve as intermediate “staging posts” in the upconversion pathway. The efficiency of these transitions is strongly governed by molecular packing and intermolecular interactions in the crystalline state (Table S21). Single-crystal structural analysis reveals the presence of multiple short-range interactions, including C–H···π interactions with distances ranging from 2.683 to 2.930 Å and π–π interactions with a centroid-to-centroid distance of 3.991 Å. Such noncovalent interactions effectively restrict molecular vibrations and suppress nonradiative decays, particularly the IC process from T_n_ to T_1_. Concurrently, intermolecular electronic coupling arising from compact molecular packing enhances SOC (Fig. S58 and Table S22), thereby facilitating *h*RISC from T_n_ to S_1_. This T_n_ → S_1_ upconversion process competes effectively with T_n_ → T_1_ conversion, ultimately giving rise to dual delayed emission: DF arising from the T_n_ → S_1_ → S_0_ pathway and phosphorescence originating from the T_n_ → T_1_ → S_0_ pathway. π–π coupling in the crystal further enlarges the energy gap between delayed fluorescence and phosphorescence by lowering the T_1_ energy level, thereby enabling the blue-red afterglow switching with a significant color change.

Femtosecond and nanosecond time-resolved transient absorption (TA) spectroscopy were employed to investigate the excited-state dynamics of the 1,2-NpAc crystal across multiple timescales. Following photoexcitation (*λ*_ex_ = 365 nm) at 220 K and 298 K, two primary excited-state absorption (ESA) bands are observed at ~600–700 nm and ~800–900 nm (Fig. 4d, e). Notably, at 298 K, the ESA peak around 780 nm became significantly more pronounced within 0.1–0.66 ps, indicating that temperature affects the excited-state processes and promotes additional population redistribution processes. This complexity is further underscored by nanosecond TA measurements (Fig. S59), which track the subsequent population evolution. The data reveal a clear spectral cascade: the ESA features at approximately 450 and 600 nm emerge promptly, followed by the rise of a broad 700–800 nm band within 1.5–2.5 ns. This sequential spectral evolution spanning the ultrafast to nanosecond timescale indicates that the excited-state relaxation proceeds through multiple pathways, accompanied by population redistribution among different excited states. Such a multichannel excited-state landscape provides the possibility for triplet excitons to undergo both RISC to S_1_ state to generate DF and radiative decay to the ground state to produce phosphorescence (Tables S18–S23).

Furthermore, the excited-state dynamics in DF and phosphorescence emission with large ∆*E*_ST_ in the 1,2-NpAc crystal were investigated by theoretical calculations. From the calculation data of 1,2-NpAc trimer extracted from the crystal structure, the HLCT-type excited state results in small energy gaps between T_n_ and S_1_, owing to the higher CT contribution in both states. Notably, the energy gap between T_10_ and S_1_ is only 0.06 eV, accompanied by a large SOC value of 0.2087 cm^−1^ (Fig. 4f) (T_10_ is selected from the lowest triplet state satisfying S_1_–T_n_ < 0.3 eV to enable efficient ISC). Although the T_n_ → T_1_ IC process is generally efficient, its rate is governed by both the nonadiabatic vibronic coupling and the T_n_–T_1_ energy gap. In the crystalline state, the rigid π-conjugated structure and strong intermolecular interactions effectively restrict molecular motions, thereby weakening vibronic coupling, while the large T_10_–T_1_ gap (~1.06 eV by calculation) further suppresses IC process. Meanwhile, the small T_10_–S_1_ gap facilitates *h*RISC, allowing T_n_ states to repopulate S_1_. This balance suppresses nonradiative decay of T_n_ excitons while promoting T_n_-mediated DF-type afterglow via the T_n_ → S_1_ pathway. Additionally, the possible changes upon temperature change in the crystal structure were determined by in situ X-ray single-crystal diffraction across the temperature range of 220–380 K. As the temperature increased, the crystal volume of 1,2-NpAc slightly expanded from 6980.74 Å^3^ (220 K) to 7148.00 Å^3^ (380 K). The degree of expansion is 2.4%, lower than that of 1,2-PhAc (2.5%) and Ph-Ac (3.3%) crystals (Fig. S60 and Table S23). Accordingly, the intermolecular π–π stacking distance in 1,2-NpAc crystals slightly increased from 3.902 to 4.066 Å (Fig. 4g, h), while the C–H···π interactions slightly increased from 2.641, 2.671, and 2.870 Å to 2.717, 2.740 and 2.974 Å, respectively (Fig. S61). The molecular conformation was almost maintained with the ignorable changes of dihedral angles among phenyl moieties (θ_1_, θ_2_, and θ_3_, which were labeled in Fig. S42) from 51.77° to 51.60°, 39.38° to 38.78°, and 9.16° to 9.17°, respectively (Fig. 4i). Thus, emission modulation due to temperature-induced changes in molecular packing can be discounted, as the crystal structure exhibits only negligible variations across the studied temperature range. These subtle changes from low temperature to high temperature were mainly due to the rigid molecular structure with extended conjugation and the staggered arrangement of 1,2-NpAc with the twisted molecular conformation, which can suppress the possible molecular motions during the heating process as nonradiative decays by strong intra/intermolecular interactions, ensuring the bright afterglow emission at high temperature. Additionally, the possibility of defect-related emission was excluded by the similar excitation spectra obtained for emissions at 450, 485, 620, and 680 nm (Fig. S62). Collectively, these spectroscopic tests, together with the analyses of temperature-dependent exciton dynamics, crystal structures, and theoretical calculations, provide strong support for T_n_-mediated DF and phosphorescence mechanism.

Overall, as the π-conjugation extends from Ph-Ac to 1,2-PhAc and further to 1,2-NpAc through the annulation strategy, the temperature-dependent shift in delayed emission wavelength progressively increases, from 15 nm (Ph-Ac) to 45 nm (1,2-PhAc) and ultimately to 170 nm (1,2-NpAc), across the temperature range of 220–380 K. This trend is primarily attributed to the T_n_-mediated dual-emission mechanism in the 1,2-NpAc crystal, which features a large energy gap (0.76 eV) between the two emission channels and exhibits distinguished temperature sensitivity (Fig. 4j). Upon structural optimization from Ph-Ac to 1,2-PhAc, the annulation strategy enables extended π-conjugation and a rigid molecular structure, which, in conjunction with the resulting close stacking in the crystalline state, helps suppress nonradiative decay of excited triplet states. The further extended conjugation and twisted conformation of 1,2-NpAc promote favorable molecular packing with efficient electronic coupling. This combined effect, arising from both molecular conformation and intermolecular interactions, not only widens the energy gap between T_n_ and T_1_ to suppress IC but also enhances SOC to facilitate the *h*RISC process from T_n_ to S_1_. As a result, efficient dual emission is achieved in the crystalline state, proceeding via T_n_ → S_1_ → S_0_ and T_n_ → T_1_ → S_0_ transitions, respectively. Moreover, these two T_n_-related pathways can be modulated by temperature as “control knobs”, and the T_n_ → S_1_ → S_0_ transitions as DF were dominant at room temperature and high temperature (298–380 K), while T_n_ → T_1_ → S_0_ transitions as phosphorescence occur at low temperature (260–220 K). Thus, 1,2-NpAc crystal with a high-contrast temperature-responsive afterglow color shift from blue to red with a record change (∆*λ* = 170 nm) of emission wavelengths.

With the full investigation of T_n_ states with modulated transition pathways, three key factors have been summarized: (1) the formation of HLCT-excited states by the tunable electronic properties, which facilitates the population of T_n_ states; (2) the construction of rigid π-conjugated structure with twisted molecular conformations by tuable substitution positions, which suppresses nonradiative decay of excited triplet states and promotes the preferred molecular packing; (3) the presence of π–π electronic coupling in the crystalline state. These factors collectively enhance SOC between T_n_ and S_1_, and increase the energy gap between T_n_ and T_1_, thereby enabling two distinct transition pathways, T_n_ → S_1_ → S_0_ and T_n_ → T_1_ → S_0_. This results in dual emission comprising DF and phosphorescence, with a significant difference in emission wavelengths.

### Application

**Fig. 5:**
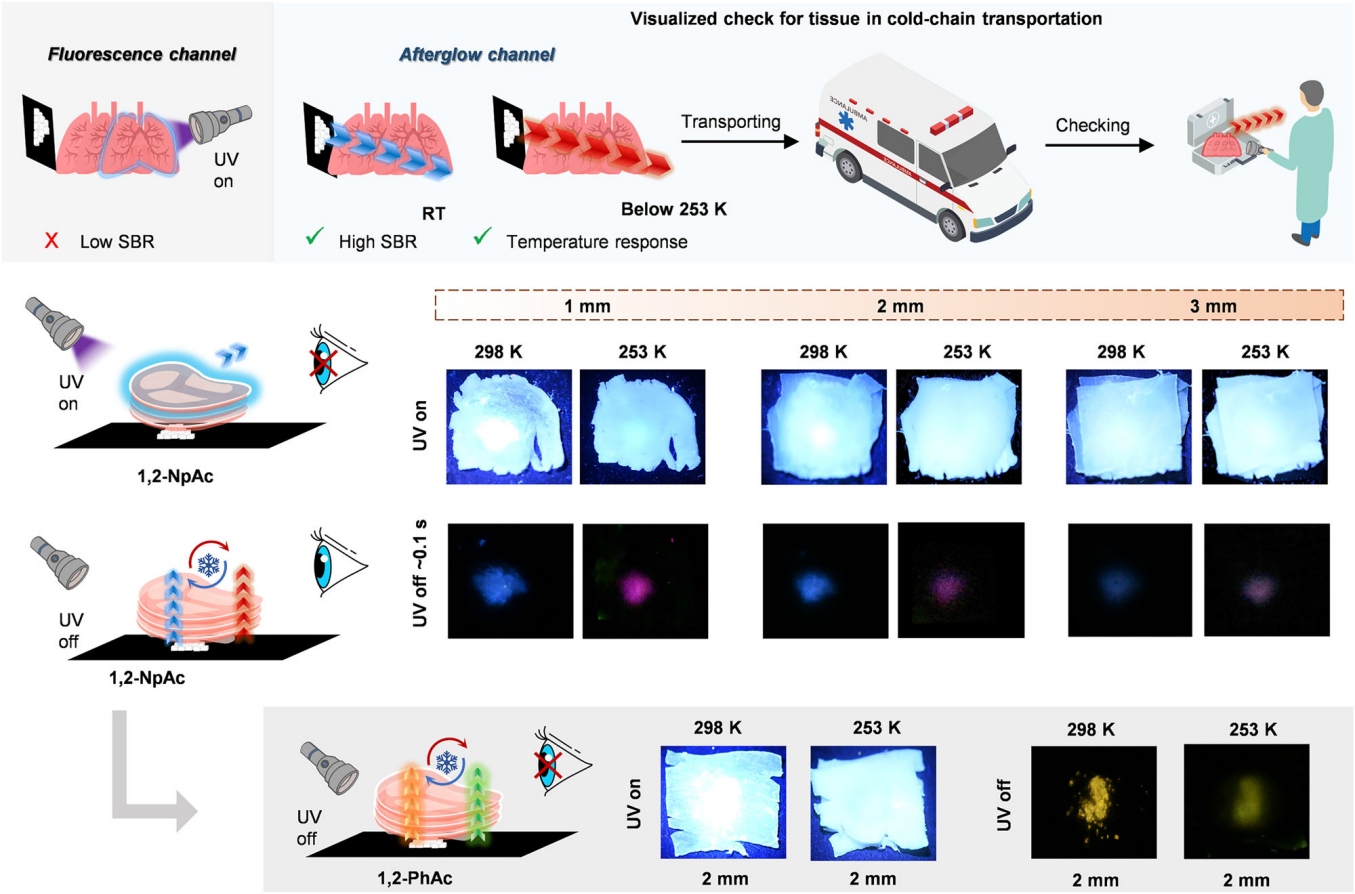
Application of 1,2-NpAc crystal in real-time temperature visualization. Schematics of biological tissue penetration depth experiments conducted in fluorescence and afterglow channels, along with visual inspection for tissue tracking during cold-chain transportation. The photographs of 1,2-NpAc crystals placed beneath chicken breast tissue slices with thicknesses of 1, 2, and 3 mm, and 1,2-PhAc crystals placed beneath 2 mm thick tissue slices in fluorescence and afterglow channels. (SBR: signal-to-background ratio).

Based on the temperature-dependent afterglow variation with high-contrast and persistent emission, the 1,2-NpAc crystal was applied in temperature-responsive pattern displays, time-temperature dual-mode anti-counterfeiting, and real-time temperature monitoring. Generally, biological tissues are susceptible to temperature fluctuations, with even minor changes potentially affecting their functions and leading to possible putrefaction. Although cold-chain systems provide controlled temperature throughout transportation, the precise real-time temperature visual detection within biological tissues remains a challenge due to the possible background interference of the conventional fluorescence probes. Afterglow with time-resolved characteristics offers higher signal-to-background ratios (SBR) in bioimaging^49,50^, showing potential for accurate temperature monitoring and imaging under low-temperature conditions. As shown in Fig. 5, 1,2-NpAc crystals were placed beneath chicken breast tissue slices with thicknesses of 1, 2, and 3 mm. In fluorescence mode, the emission signal was masked by the strong autofluorescence of biological tissue at all thicknesses.

Once afterglow mode was employed, only the blue afterglow of the 1,2-NpAc crystal could be observed, effectively shielding interference from the autofluorescence of biological tissues. As the temperature decreased to 253 K (253 K is commonly used as the standard transportation temperature for biological tissues to maintain structural and biochemical stability), this blue afterglow gradually shifted to red. Such a pronounced color transition allowed clear visual detection of temperature variations through up to 3 mm of chicken breast tissue slices, thereby demonstrating the potential for reliable temperature monitoring within deep biological tissues. To further validate the advantages of this high-contrast afterglow color change in temperature variation, the 1,2-PhAc crystal was incorporated as the reference, which exhibited orange-green afterglow-response with the temperature variations from 298 to 253 K. Although the sample position remained detectable at both room and low temperatures, the minimal difference in afterglow emission color limited the accuracy of temperature discrimination, even though the thickness of chicken breast tissue decreased to 2 mm. Only 1,2-NpAc crystal with high constant temperature-modulated afterglow facilitated reliable, real-time visual temperature detection, which exhibited great potential in real-time temperature visualization in cold-chain logistics and other environments requiring remote, noninvasive thermal monitoring. Besides, various patterns and signals with the time-temperature dual-mode anti-counterfeiting have been realized by 1,2-PhAc and 1,2-NpAc, significantly improving information security and encryption diversity (Figs. S63 and S64).

## Discussion

In summary, the transition pathways of T_n_ states with high energy levels have been regulated by the rational modulation of heterocyclic systems bearing fused-ring structures, together with temperature variations. In the low temperature (220K–260K), the T_n_ → T_1_ → S_0_ transitions of 1,2-NpAc crystal as the dominant pathway resulted in the bright red phosphorescence emission, while it converted to T_n_ → S_1_ → S_0_ transitions for the temperature in the 298–380 K region, leading to the DF-type afterglow with blue emission. A large energy gap of 0.76 eV between DF and phosphorescence emission has been achieved, corresponding to a record emission wavelength shift of 170 nm. This is attributed to the balanced regulation of T_n_-mediated transition pathways through modulation of the electronic properties, molecular conformation, and intermolecular interactions in the crystalline state. These factors collectively enhance SOC between T_n_ and S_1_ while increasing the energy gap between T_n_ and T₁, thereby enabling two distinct transition pathways, T_n_ → S_1_ → S_0_ and T_n_ → T_1_ → S_0_, with distinct temperature sensitivities. This work demonstrated a precise and reversible external control mechanism over T_n_ transition pathways, offering an elegant solution to resolving the key challenge in T_n_ manipulation and unlocking their untapped potential for photonic applications.

## Methods

### Reagents

Unless otherwise noted, all reagents used in the experiments were purchased from Sinopharm Chemical Co., Ltd. The potassium tert-butoxide (purity: 95%) was purchased from Aladdin Scientific. The phenoxazine (purity: 98%), phenothiazine (purity: 98%), 9,9-dimethyl-9,10-dihydroacridine (purity: 98%), 2-bromoiodobenzene (purity: 98%), 1-bromo-2-iodonaphthalene (purity: 95%), 2,3-dibromonaphthalene (purity: 98%) and *trans*-dichlorobis (tricyclohexylphosphine)palladium (II) (purity: 98%) were purchased from Bidepharm. Palladium acetate (purity: 99%) and tri-*tert*-butylphosphine tetrafluoroborate (purity: 98%) were purchased from Energy Chemical.

### Instruments

^1^H and ^13^C NMR spectra were characterized on a Bruker Avance III HD 400 MHz or Bruker AVANCE NEO 400 MHz. Mass spectra were recorded on a Shimadzu GCMS-QP2020 mass spectrophotometer. Elemental analyses were conducted on a UNICUBE. Photoluminescence and phosphorescence spectra in aggregated and solution states were determined on a FLS980 spectrometer. UV–vis absorption spectra were measured on a Shimadzu UF5700 spectrometer. Lifetimes and quantum yields were measured with the FLS980 spectrometer. The single-crystal X-ray diffraction data were collected on an XtaLAB Synergy Custom and a Bruker D8. The transient absorption was measured using a Dalian Chuangrui instrument (TA100-DZ) and Ultrafast Systems (HELIOS). All the structures were resolved and analyzed with the assistance of Olex2 software. High-performance liquid chromatography (HPLC) was conducted on LaboACE LC-500. The photos were taken by Nikon Z9.

### Theoretical calculation

TD-DFT/DFT calculations were performed on the Gaussian 09 program (Revision D01) and Gaussian 16, Revision C.011^46^. The excitation energies in the n-th singlet (S_n_) and n-th triplet (T_n_) states of monomer and trimer were obtained using the PBE0 functional with def2svp basis, based on the structure extracted from single crystal diffraction data. The spin-orbital coupling (SOC) values were performed through the same functional and basis using the PySOC package with Python 2.7. The noncovalent interaction (NCI) analysis, interaction region indicator (IRI) and electrostatic potential analysis were performed through Multiwfn 3.8^42^ and VMD^43^.

### Crystal growth

The Ph-PTZO, 1,2-PhPOZ, 1,2-PhPTZ, 1,2-PhAc, 1,2-PhPTZO, 1,2-PhTZOO, and 1,2-NpAc crystal cultured through slow solvent evaporation in a mixed solvent of dichloromethane and methanol with a volume ratio of 1:5. The 2,3-NpAc crystal was cultured through slow solvent evaporation in a mixed solvent of diethyl ether and methanol with a ratio of 1:2 and stored at 4 °C.

### Preparation of 1,2-PhAc@epoxy resin and 1,2-NpAc@epoxy resin

1 mg of the emitter (1,2-PhAc or 1,2-NpAc) and 1 g of diphenylolpropane diglycidyl ether were weighed into a separate sample bottle of 5 mL. The emitters and resin mixture was heated and stirred constantly at 100 °C until they dissolved and mixed evenly, then cooled down to 25 °C. The curing agents diethylenetriamine were then added to each sample bottle, and stirring continued for 5 min to obtain a viscous prepolymer. Then, the injected mixture was placed into a mold (0.6 cm in diameter and 0.3 cm in height) and heated at 100 °C for 12 h. After solidification and cooling, the samples were demolded to obtain 1,2-PhAc@epoxy resin and 1,2-NpAc@epoxy resin.

### Reporting summary

Further information on research design is available in the Nature Portfolio Reporting Summary linked to this article.

## Supplementary information

Supplementary Information

Description of Additional Supplementary Files

Supplementary Data 1

Reporting Summary

Transparent Peer Review File

## Source data

Source Data

## Data Availability

All data are available from the corresponding author upon request. The authors declare that the data supporting the findings of this study are available within the article and its Supplementary Information and Source data files. Source data are available. Coordinate files for DFT experiments are available in Supplementary Data 1. Deposition numbers 2491655 (for Ph-PTZO), 2491658 (for 1,2-PhPOZ), 2491666 (for 1,2-PhPTZ), 2491716 (for 1,2-PhAc), 2491717 (for 1,2-PhPTZO), 2491719 (for 1,2-PhPTZOO), 2491721 (for 1,2-NpAc), and 2491722 (for 2,3-NpAc) contain the supplementary crystallographic data for this paper. These data are provided free of charge by the joint Cambridge Crystallographic Data Centre and Fachinformationszentrum Karlsruhe Access Structures service. Source data are provided with this paper.
