# Supplementary material for: High energy triplet-state manipulation via temperature-responsive twisted hetero-annulation systems
Source: Nat Commun. 2026 May 25;17:6790. doi: 10.1038/s41467-026-73715-8 (PMC13385895; doi:10.1038/s41467-026-73715-8)
Supplement: Supplementary file 1 — Supplementary Information [file 41467_2026_73715_MOESM1_ESM.pdf]

Supplementary Information

Guigui Ye[1], Yan Gao[1], Wentao Yuan[1], Juqing Gu[1], Jiaqiang Wang[1], Yujie Yang[2], Qianqian Li[1], Zhen Li[1]*

[1]Hubei Key Lab on Organic and Polymeric Opto-Electronic Materials, Department of Chemistry, Wuhan University, Wuhan, China

[2]School of Sports Medicine, Wuhan Sports University, Wuhan, China

Contents

1. Supplementary Methods 5

2. Supplementary Discussion 9

2.1 NMR data and HPLC spectra 9
2.2 Photophysical property of luminogens 17
2.3 Crystal data and theoretical calculation of luminogens 31
2.4 Photophysical property of 1,2-NpAc and 2,3-NpAc 42

3. Supplementary References 55

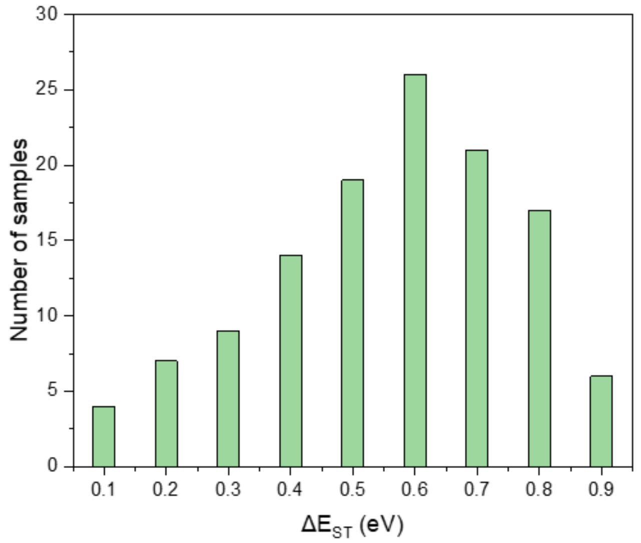

Figure S1. The frequency statistics of energy level in HLCT-emitters in previous work, with detailed information summarized in Table S1. Distribution of energy level collected from n = 123 independent samples. Histogram bin width: 0.1 eV.

Table S1. Energy gap between S_1 and T_1 (ΔE_{ST}) of HLCT-emitters in previous work

Compounds	ΔE_{ST}	Ref.
PPI-2TPA, PPI-2NPA	0.56, 0.58 eV	*J. Mater. Chem. C*, **2017**, *5*, 5402-5410
TPA-DFCP	0.37 eV	*J. Mater. Chem. C*, **2020**, *8*, 14117-14124
PyPPA, PyPPAC	0.87, 0.81 eV	*ACS Appl. Mater. Interfaces* **2020**, *12*,16715–16725
2TPA-PPI	0.68 eV	*Adv. Funct. Mater.* **2021**, *31*, 2100704
Spiro-TPA， Spiro-2P-BT-TPA	0.77, 0.87 eV	*Chem. Eng. J.* **2021**, *418*, 129366
2BuCz-CNCz	0.72 eV	*Angew. Chem. Int. Ed.* **2021**, *60*, 22241–22247
DPM, TDPM	0.56, 0.59 eV	*Dyes Pigm.* **2021**, *193*, 109482
DTPPI, CTPPI, and CBPI	0.65, 0.71, 0.55 eV	*Adv. Funct. Mater.* **2022**, *32*, 2112969
TTT-TPA-H, TTT-TPA-Ome, TTT-TPA-tBu	0.51, 0.41, 0.46 eV	*Adv. Funct. Mater.* **2022**, *32*, 2113183
2Na-CzCN	0.74 eV	*ACS Appl. Mater. Interfaces* **2022**, *14*,10627–10636
m-TAC, *p*-TAC	0.15, 0.48 eV	*Dyes Pigm.* **2022**, *204*, 110391
(R)-BBT2TPA	0.46 eV	*Adv. Funct. Mater.* **2022**, *32*, 2201512
FIP-CZ, FIP-tBuCZ, FIP-DPA, and FIP-tBuDPA	0.6 0.5, 0.55, 0.47 eV	*Adv. Mater.* **2023**, *35*, 2305310
PXZBAO, PXZBTO, PXZBPO	0.11, 0.14, 0.47 eV	*ACS Appl. Mater. Interfaces* **2023**, *15*,15758–15767
3BPy-mDCz, 3BPy-mDTA, 3BPy-mDMAC and 3BPy-mDPT	0.22, 0.20, 0.11, 0.14 eV	*J. Mater. Chem. C*, **2023**, *11*, 16368-16376
DCZ2F, DTPA2F	0.88, 0.66 eV	*J. Mater. Chem. C***,** **2023**, *11*, 6347-6353
PIS, CNPIS	0.56, 0.56 eV	*Mater. Chem. Front.*, **2023**,*7*, 85-95
TrBPI, 4PyBPI, 2PyBPI, and 2PyTPI	0.56, 0.67, 0.6, 0.86 eV	*J. Mater. Chem. C*, **2023**, *11*, 14776-14786
tBOSi, tBOSiCz	0.26, 0.21 eV	*Adv. Optical Mater.* **2023**, *11*, 2300195

BCzPPM, BCzPSP, IDCz-DBPM, IDCz-BPSP	0.38, 0.33, 0.39, 0.49 eV	*Dyes Pigm.* **2023**, *219*, 111586
DCBCPPI	0.93 eV	*J. Mater. Chem. C* **2023**, *11*, 4456-4465
m-PCZPBO, PCZPBO, 3-PCZPBO, and DTPCZPBO	0.81, 0.70, 0.76, 0.64 eV	*Chem. Eng. J.* **2023**, *472*, 144950
DPAvlBPI	0.55 eV	*J. Lumin.* **2024**, *267*, 120391
CZFPY, CZFPI, TPAFPI	0.64, 0.75, 0.55 eV	*Chem. Eng. J.* **2024**, *480*,148107
BCZ-6P,*p*Ph-6P,oPh-6P	0.62, 0.68, 0.70 eV	*J. Mater. Chem. C*, **2024**, *12*, 11085-11093
BDP-C-Cz,BDP-N-Cz	0.50, 0.55 eV	*Energy Environ. Mater.* **2024**, *7*, e12597
3,6-mPPICNC3, 3,6-pPPICNC3, 2,7-mPPICNC3 and 2,7-pPPICNC3	0.63, 0.67, 0.56, 0.54 eV	*Chem. Sci.*, **2024**, *15*, 11053-11064
PPIS, PPISCN	0.52, 0.7 eV	*Adv. Optical Mater.* **2024**, *12*, 2301413
2-*t*Bu*spo*Cz-2pTRZ，2-tBuspoCz-Me3pTRZ，2-tBuspoCz-2p:TAPC，2-tBuspoCz-Me3p:TAPC	0.37, 0.40, 0.17, 0.04 eV	*ACS Materials Lett.* **2024**, *10*, 4738–4747
2-tBuspoCz-TRZ，10-tBuspoCz-TRZ，2-tBuspoCz-TRZ:TAPC，10-tBuspoCz-TRZ:TAPC	0.30, 0.50, 0.04, 0.03 eV	*Adv. Mater.* **2024**, *36*, 2313656
Me-PT, MeO-PT, MTM-PT, TfMe-PT, TfMeO-PT and TfMTM-Pttal	0.48, 0.52, 0.49, 0.61, 0.53, 0.53 eV	*J. Mater. Chem. C*, **2024**, *12*, 14129-14138
PPT and PPPT	0.64, 0.73 eV	*J. Mater. Chem. C*, **2024**, *12*, 3881-3887
PdCz-2CN/mPdCz-2CN	0.47, 0.34 eV	*Adv. Funct. Mater.* **2024**, *35*, 2422822
mPTPH	0.6 eV	*Chem. Asian J.* **2024**, *19*, e202400957
0.06 wt % NCmO/PMMA	0.43 eV	*Angew. Chem. Int. Ed.* **2024**, *136*, e202411588
pPh-7P, pPh-7M, and pPh-6M	0.65, 0.38, 0.42 eV	*Chem. Eur. J.* **2024**, *30*, e202401078
Silole-1DPA-TRZ，Silole-1Cz-TRZ	0.23, 0.32 eV	*Adv. Funct. Mater.* **2024**, *34*, 2410250
TPI, pCTPI, and mCTPI	0.78 eV	*Dyes Pigm.* **2024**, *230*, 112349
BTPO-DPA, BTPO-2DPA	0.36, 0.22 eV	*Chem Asian J.* **2024**, *19*, e202400925
NFIP-CZ, NFIP-tBuCZ, NFIP-DPA and NFIP-tBuDPA	0.66, 0.49, 0.54, 0.48 eV	*Angew. Chem. Int. Ed.* **2025**, *64*, e202414905
DtCzB-SFN，DtCzB-DFN	0.09, 0.12 eV	*Chem. Eng. J.* **2025**, *504*, 158909
PdCz-2CN，mPdCz-2CN	0.47, 0.34 eV	*Adv. Funct. Mater.* **2025**, *35*, 2422822
ICz-BO	0.35 eV	*Angew. Chem. Int. Ed.* **2025**, *64*, e202421520
o-PIOXZ, m-PIOXZ, and m-CZOXZ	0.62, 0.76, 0.77 eV	*Chem. Sci.*, **2025**, *16*, 5252-5259
BO-N	0.46 eV	*Angew. Chem. Int. Ed.* **2025**, *64*, e202505328
2tBuCz-APO,2TPA-APO	0.60, 0.27 eV	*Dyes Pigm.* **2025**, *235*, 112587
Silole-2,5DPA-TRZ and Silole-2,5Cz-TRZ	0.54, 0.20 eV	*J. Mater. Chem. C*, **2025**, *13*, 10053-10061
PICZ4F, PI3CZ4F	0.55, 0.47 eV	*Mater. Chem. Front.*, **2025**, *9*, 55-64
4Ph-TPA-BTD, 3Ph-TPA-BTD	0.59, 0.70 eV	*Dyes Pigm.* **2025**, *241*, 112905
TPAPBO, POXPBO	0.63, 0.38 eV	*Dyes Pigm.* **2025**, *241*, 112905

Table S2. Wavelength difference and energy gap between DF and phosphorescence in single-component systems

Compounds	λ_{DF}(nm)	λ_{Phos}(nm)	Energy gap (eV)	Ref.
CB-I	438	490	0.30	*Carbon* **2023**, *208*, 365–373
ACD-AA @PAM	440	495	0.31	*Chem. Eng. J.* **2023**, 463, 142506
PI2@PAM	440	500	0.56	*Angew. Chem. Int. Ed.* **2024**, *63*, e202410974
TCAG of CDs@Paper	500	560	0.26	*Adv. Mater.* **2024**, *36*, 2403775
3-PhB-0.001%	420	483	0.38	*Chem. Eur. J.* **2024**, *30*, e202303834
5-PhB-0.001%	410	473	0.40	*Chem. Mater.* **2024**, *36*, 3000−3012
FluNa-0.005 wt%-$Al_2(SO_4)_3$	483	549	0.31	*Angew. Chem. Int. Ed.* **2023**, *62*, e202217616
FNCDs	530	598	0.27	*Adv. Funct. Mater.* **2024**, *34*, 2405669
Acf@PVA	523	598	0.30	*Angew. Chem. Int. Ed.* **2023**, *62*, e202304020
TCPB@BO	450	530	0.54	*Chem. Eng. J.* **2025**, *516*, 164264
BrBF2-PhB	465	550	0.41	*J. Am. Chem. Soc.* **2024**, *146*, 24871−24883
IB-BF2-IB	660	760	0.25	*Angew. Chem. Int. Ed.* **2024**, e202418097
IbCzA-0.1 %-PVA	410	480	0.44	*Angew. Chem. Int. Ed.* **2022**, *61*, e202201820
BA@CE	450	560	0.54	*Angew. Chem. Int. Ed.* **2025**, *64*, e202421036
ICZ-p1-0.5 wt.%-PMMA	435	525	0.49	*Adv. Funct. Mater.* **2024**, *34*, 2402428
NPYC4-Py	473	557	0.40	*Angew. Chem. Int. Ed.* **2024**, e202417868

The energy gap was calculated using the equation $\Delta E = 1240/\lambda_{DF}-1240/\lambda_{Phos}$.

1. Supplementary Methods

1.1 Materials

All starting chemicals were purchased from commercial sources and used without further purification. Toluene was distilled under an argon atmosphere with Na-K alloy before use. Column chromatography was carried out on silica gel with 200-300 mesh.

1.3 Synthetic procedures

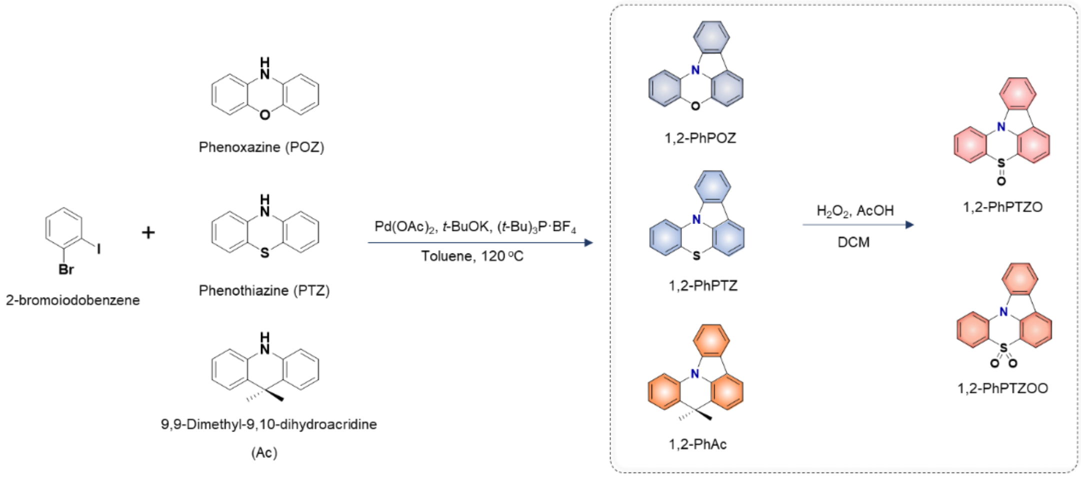

Figure S2. Synthetic routes of compound 1,2-PhPOZ, 1,2-PhPTZ, 1,2-PhAc, 1,2-PhPTZO and 1,2-PhPTZOO.

The general synthetic route of 1,2-PhPOZ, 1,2-PhPTZ and 1,2-PhAc.

Phenoxazine, phenothiazine or 9,9-dimethyl-9,10-dihydroacridine (3.0 mmol, 1.5 equiv.), 2-bromoiodobenzene (2.0 mmol, 1 equiv.), potassium *tert*-butoxide (6.0 mmol, 3 equiv.), palladium acetate (0.2 mmol, 0.1 equiv.) and tri-*tert*-butylphosphine tetrafluoroborate (0.6 mmol, 0.3 equiv.) were dissolved in toluene (10 mL) in a Schlenk tube. The resultant mixture was refluxed at 120 °C for 12 hours under nitrogen atmosphere, then extracted with dichloromethane. The combined organic extracts were dried over anhydrous Na_2SO_4 and concentrated by rotary evaporation. The crude product was purified by column chromatography on silica gel using petroleum ether and dichloromethane as eluents.

Compound 1,2-PhPOZ. A white solid (218.9 mg, 42.58 %). ^{1}H NMR (400 MHz, $CDCl_3$) δ (ppm): 8.02 (d, J = 7.8 Hz, 1H, ArH), 7.87 (d, J = 8.4 Hz, 1H, ArH), 7.69-7.62 (m, 1H, ArH), 7.55-7.47 (m, 1H, ArH), 7.43 (d, J = 7.9 Hz, 1H, ArH), 7.29 (t, J = 7.5 Hz, 1H, ArH), 7.02 (t, J = 7.9 Hz, 2H, ArH), 6.99-6.89 (m, 2H, ArH), 6.70 (d, J = 7.8 Hz, 1H, ArH). ^{13}C NMR (100 MHz, $CDCl_3$) δ (ppm): 145.58, 142.71, 136.65, 128.98, 128.85, 126.53, 125.12, 124.24, 123.99, 123.01, 122.93, 121.42, 120.58, 117.55, 114.33, 113.89, 112.21, 108.59. MS (EI, m/z): $[M]^+$ calcd for $C_{18}H_{11}NO$: 257.08, Found: 257.15. Anal. Cacld for $C_{18}H_{11}NO$: C, 84.03, H, 4.31, N, 5.44, Found: C, 84.18, H, 4.10, N, 5.34.

Compound 1,2-PhPTZ. A white solid (222.7 mg, 40.78 %). ^{1}H NMR (400 MHz, $CDCl_3$) δ (ppm): 8.07-7.99 (m, 2H, ArH), 7.88-7.80 (m, 1H, ArH), 7.63-7.65 (m, 1H, ArH), 7.49-7.53 (m, 1H, ArH), 7.34-7.30 (m, 1H, ArH), 7.22-7.11 (m, 3H, ArH), 7.05-6.95 (m, 2H, ArH). ^{13}C NMR (100 MHz, $CDCl_3$) δ (ppm): 138.62, 138.24, 136.93, 128.21, 127.59, 126.38, 126.04, 124.37, 123.76, 122.77, 121.89, 121.51, 121.14, 120.89, 117.44, 117.06, 115.33, 114.41. MS (EI, m/z): $[M]^+$ calcd for $C_{18}H_{11}NS$: 273.06, Found: 273.10. Anal. Cacld for $C_{18}H_{11}NS$: C, 79.09, H, 4.06, N, 5.12, S, 11.73, Found: C, 79.02, H, 4.14, N, 5.19, S, 11.76.

Compound 1,2-PhAc. A white solid (292.5 mg, 51.65 %). ^{1}H NMR (400 MHz, $CDCl_3$) δ (ppm): 8.19-8.06 (m, 3H, ArH), 7.89 (d, J = 7.6 Hz, 1H, ArH), 7.63 (d, J = 7.9, 1H, ArH), 7.54 (t, J = 8.5 Hz, 1H, ArH), 7.47 (d, J = 7.4 Hz, 1H, ArH), 7.35 (t, J = 7.6 Hz, 3H, ArH), 7.20-7.14 (m, 1H, ArH), 1.74 (s, 6H, $-CH_3$). ^{13}C NMR (100 MHz, $CDCl_3$) δ (ppm): 138.20, 136.06, 135.95, 134.36, 129.44, 127.26, 127.06, 126.27, 126.18, 123.20, 122.35, 121.90, 121.55, 121.01, 120.74, 117.40, 113.92, 113.62, 36.77, 32.53. MS (EI, m/z): $[M]^+$ calcd for $C_{21}H_{17}N$: 283.14, Found: 283.25. Anal. Cacld for $C_{21}H_{17}N$: C, 89.01, H, 6.05, N, 4.94, Found: C, 88.81, H, 5.97, N, 4.99.

The synthetic route of 1,2-PhPTZO and 1,2-PhPTZOO

1,2-PhPTZ (1.0 mmol, 273.4 mg) was dissolved in dichloromethane (10 mL), acetic acid (5 mL), and H_2O_2 (0.5 mL), and the mixture was stirred at 60 °C for 8 hours. The reaction mixture was then extracted with dichloromethane and purified by column chromatography. Elution with dichloromethane/petroleum ether (2:1, *v/v*) first afforded 1,2-PhPTZOO ($R_f \approx 0.5$). Increasing the eluent polarity to dichloromethane/ethyl acetate (10:1, *v/v*) subsequently yielded 1,2-PhPTZO ($R_f \approx 0.3$).

Compound 1,2-PhPTZO. A white solid (143.9 mg, 49.80 %). ^1H NMR (400 MHz, $CDCl_3$) δ (ppm): 8.43 (d, *J* = 8.5Hz, 1H, ArH), 8.33-8.22 (m, 2H, ArH), 8.18 (d, *J* = 7.7 Hz, 2H, ArH), 8.08 (d, *J* = 7.7 Hz, 1H, ArH), 7.71-7.76 (m, 1H, ArH), 7.68-7.59 (m, 2H, ArH), 7.45 (m, 2H, ArH). ^{13}C NMR (100 MHz, $CDCl_3$) δ (ppm): 138.82, 135.07, 134.01, 133.31, 133.24, 127.73, 127.46, 126.10, 125.18, 124.38, 124.04, 123.62, 123.09, 121.52, 115.83, 114.84. MS (EI, m/z): $[M]^+$ calcd for $C_{18}H_{11}NOS$: 289.06, Found: 289.15. Anal. Cacld for $C_{18}H_{11}NOS$: C, 74.72, H, 3.83, N, 4.84, O: 5.53, S: 11.08, Found: C, 74.41, H, 3.81, N, 4.74, O, 5.90, S, 11.29.

Compound 1,2-PhPTZOO. A white solid (129.1 mg, 44.65 %). ^1H NMR (400 MHz, $CDCl_3$) δ (ppm): 8.37-8.30 (m, 2H, ArH), 8.28 (d, *J* = 7.6 Hz, 1H, ArH), 8.16 (t, *J* = 8.2 Hz, 3H, ArH), 7.77-7.70 (m, 1H, ArH), 7.63 (t, *J* = 7.8 Hz, 2H, ArH), 7.49-7.44 (m, 2H. ArH). ^{13}C NMR (100 MHz, $CDCl_3$) δ (ppm): 138.85, 136.33, 135.45, 133.34, 128.09, 127.16, 125.67, 125.31, 124.36, 124.31, 123.51, 123.43, 121.68, 120.27, 115.67, 114.76. MS (EI, m/z): $[M]^+$ calcd for $C_{18}H_{11}NO_2S$: 305.05, Found: 305.10. Anal. Cacld for $C_{18}H_{11}NO_2S$: C, 70.80, H, 3.63, N, 4.59, O, 10.48, S, 10.50, Found: C, 71.00, H, 3.31, N, 4.41, O, 10.76, S, 10.41.

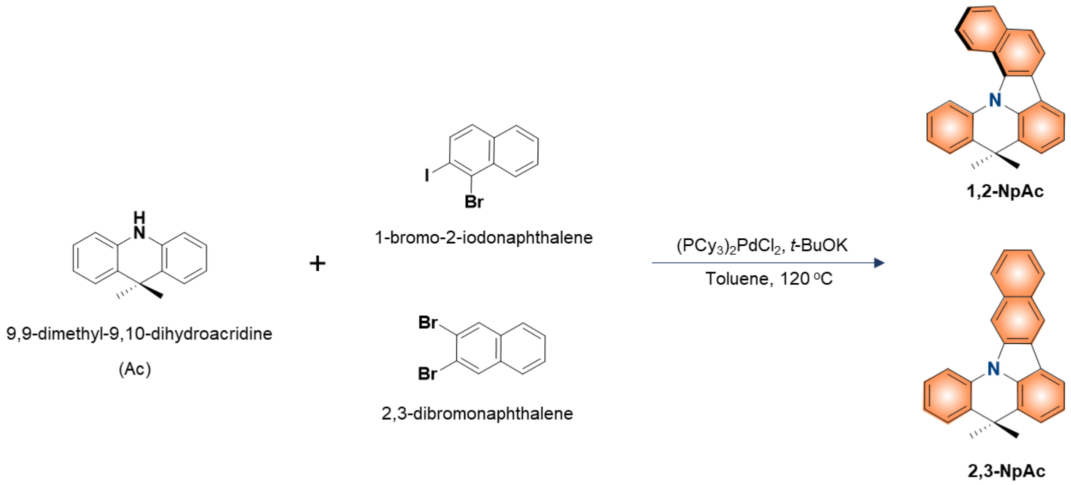

Figure S3. Synthetic routes of compound 1,2-NpAc and 2,3-NpAc.

The synthetic route of 1,2-NpAc and 2,3-NpAc

9,9-Dimethyl-9,10-dihydroacridine (3.0 mmol, 1.5 equiv.), 1-bromo-2-iodonaphthalene/2,3-dibromonaphthalene (2.0 mmol, 1 equiv.), potassium *tert*-butoxide (6.0 mmol, 3 equiv.) and *trans*-dichlorobis (tricyclohexylphosphine)palladium (II) (0.2 mmol, 0.1 equiv.) were dissolved in toluene (10 mL) in a Schlenk tube. The resultant mixture was refluxed at 120 °C for 12 hours under nitrogen atmosphere, then extracted with dichloromethane. The combined organic extracts were dried over anhydrous Na_2SO_4 and concentrated by rotary evaporation. The crude product was purified by column chromatography on silica gel with petroleum ether/dichloromethane (100:1, v/v) as the eluent.

Compound 1,2-NpAc. A white solid (192.8 mg, 38.85 %). ^1H NMR (400 MHz, $CDCl_3$) δ (ppm): 8.53 – 8.39 (m, 1H, ArH), 8.16 (d, *J*

= 8.4 Hz, 1H, ArH), 8.08 – 7.97 (m, 1H, ArH), 7.92 (d, *J* = 7.6 Hz, 1H, ArH), 7.84 (d, *J* = 8.4 Hz, 1H, ArH), 7.74 (d, *J* = 7.5 Hz, 1H, ArH), 7.62 (d, *J* = 7.4 Hz, 1H, ArH), 7.54 (m, 2H, ArH), 7.48 (d, *J* = 7.4 Hz, 1H, ArH), 7.41 (t, *J* = 7.5, 1H, ArH), 7.28 – 7.14 (m, 3H, ArH), 2.04 (s, 3H, $-CH_3$), 1.58 (s, 3H, $-CH_3$). ^{13}C NMR (100 MHz, $CDCl_3$) δ (ppm): 140.35, 137.55, 135.61, 135.07, 133.41, 130.99, 129.15, 126.12, 125.27, 125.02, 124.97, 124.82, 124.62, 123.96, 123.77, 123.70, 123.23, 123.18, 119.79, 119.41, 117.42, 116.76, 37.93, 32.17. MS (EI, m/z): $[M]^+$ calcd for $C_{25}H_{19}N$: 333.15, Found: 333.20. Anal. Cacld for $C_{25}H_{19}N$: C, 90.06, H, 5.74, N, 4.20, Found: C, 89.68, H, 5.54, N, 4.22.

Compound 2,3-NpAc. A white solid (188.7 mg, 41.24 %). ^{1}H NMR (400 MHz, $CDCl_3$) δ (ppm): 8.57 (d, *J* = 3.6 Hz, 1H, ArH), 8.48 (d, *J* = 3.7 Hz, 1H, ArH), 8.28 (d, *J* = 8.1 Hz, 1H, ArH), 8.09-7.97 (m, 3H, ArH), 7.65 (d, *J* = 7.9 Hz, 1H, ArH), 7.59-7.30 (m, 6H), 7.22-7.18 (m, 1H, ArH), 1.76 (s, 6H, $-CH_3$). ^{13}C NMR (100 MHz, $CDCl_3$) δ (ppm): 129.12, 128.73, 128.14, 127.87, 127.57, 127.20, 127.20, 125.55, 123.90, 123.11, 122.93, 122.48, 121.84, 119.18, 118.07, 113.69, 109.67, 109.59, 36.74, 32.42. MS (EI, m/z): $[M]^+$ calcd for $C_{25}H_{19}N$: 333.15, Found: 333.20. Anal. Cacld for $C_{25}H_{19}N$: C, 90.06, H, 5.74, N, 4.20, Found: C, 89.69, H, 5.67, N, 4.23.

2. Supplementary Discussion

2.1 NMR data and HPLC spectra

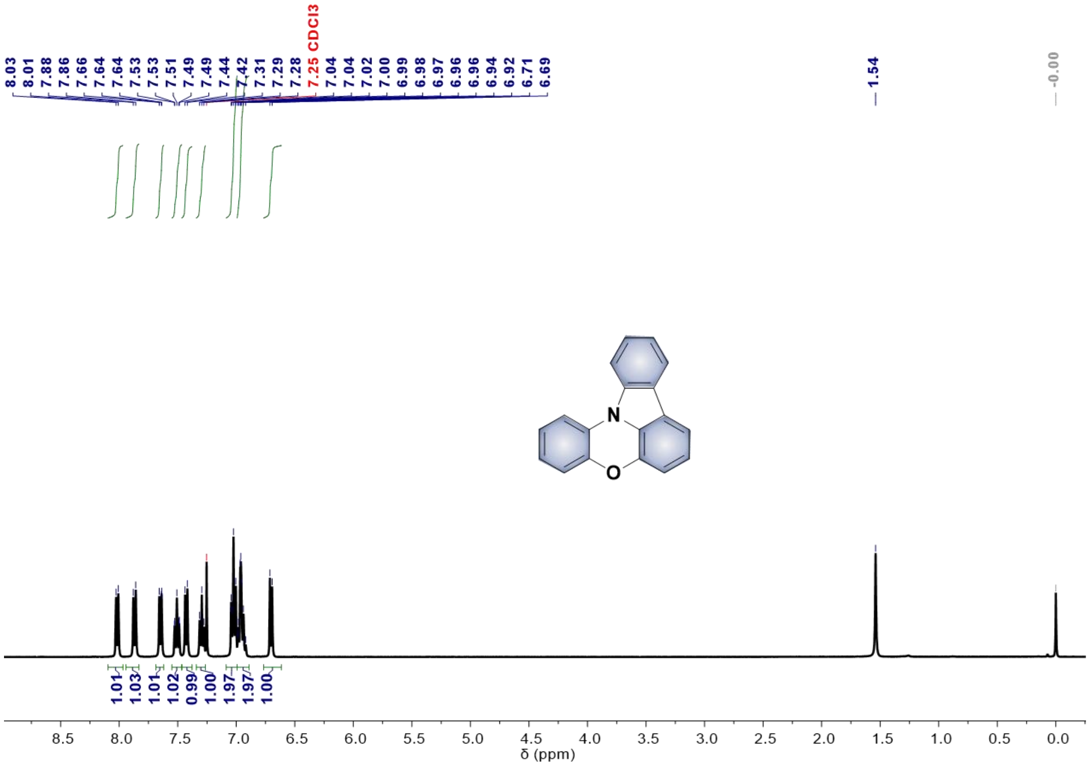

Figure S4. ^{1}H NMR（400 MHz) spectrum of 1,2-PhPOZ in $CDCl_3$.

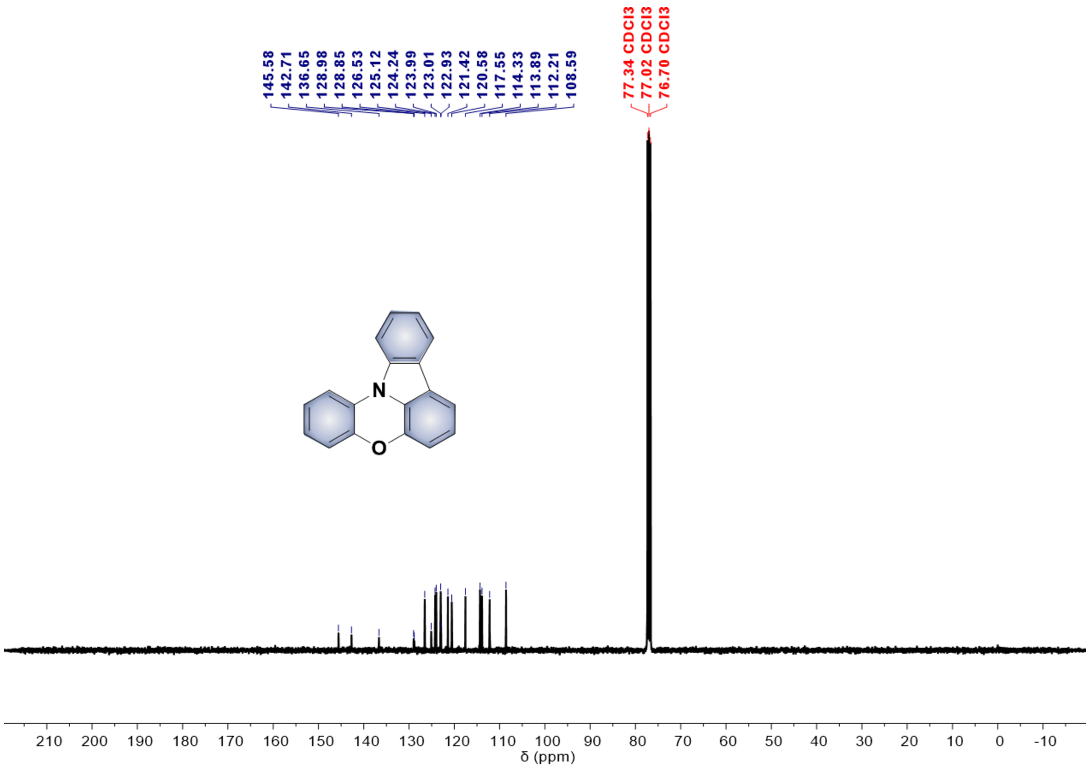

Figure S5. ^{13}C NMR（100 MHz) spectrum of 1,2-PhPOZ in $CDCl_3$.

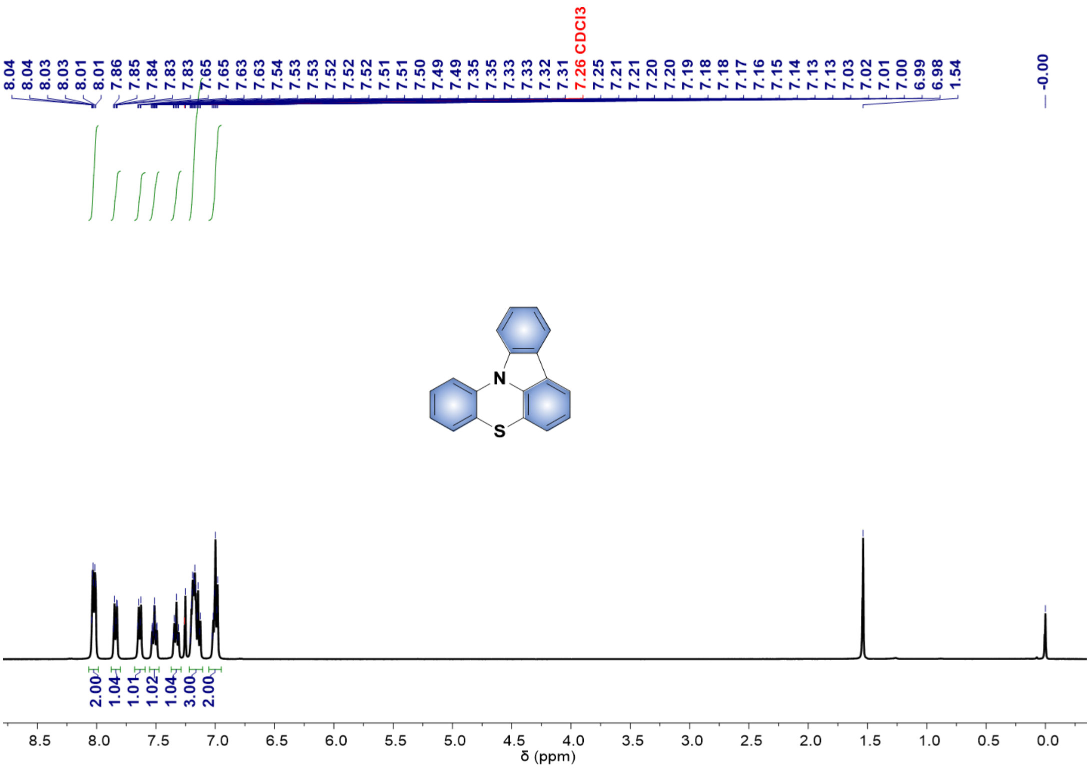

Figure S6. ^1H NMR（400 MHz) spectrum of 1,2-PhPTZ in $CDCl_3$.

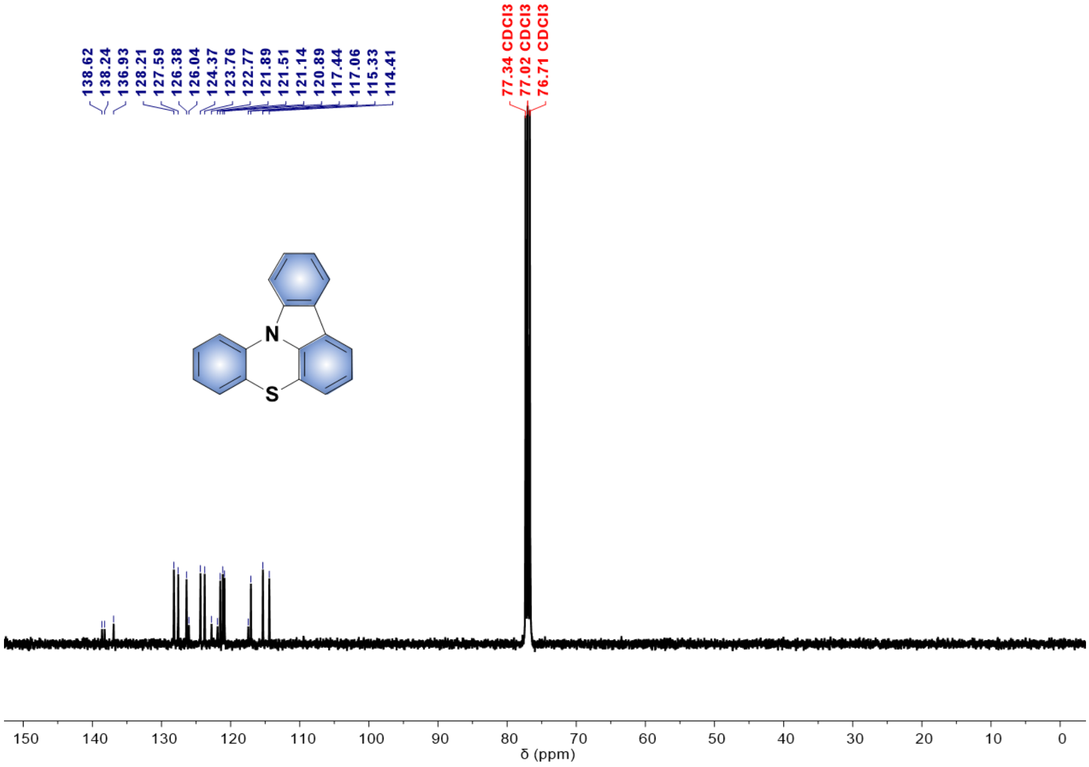

Figure S7. ^{13}C NMR（100 MHz) spectrum of 1,2-PhPTZ in $CDCl_3$.

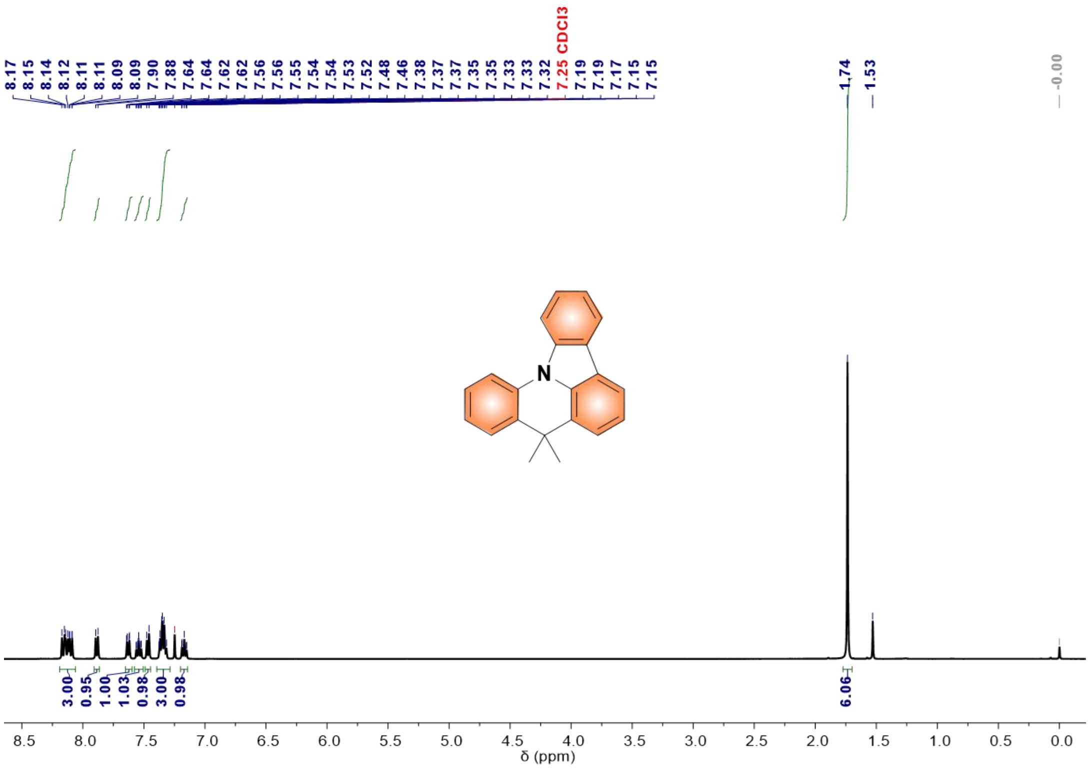

Figure S8. ^{1}H NMR（400 MHz) spectrum of 1,2-PhAc in $CDCl_3$.

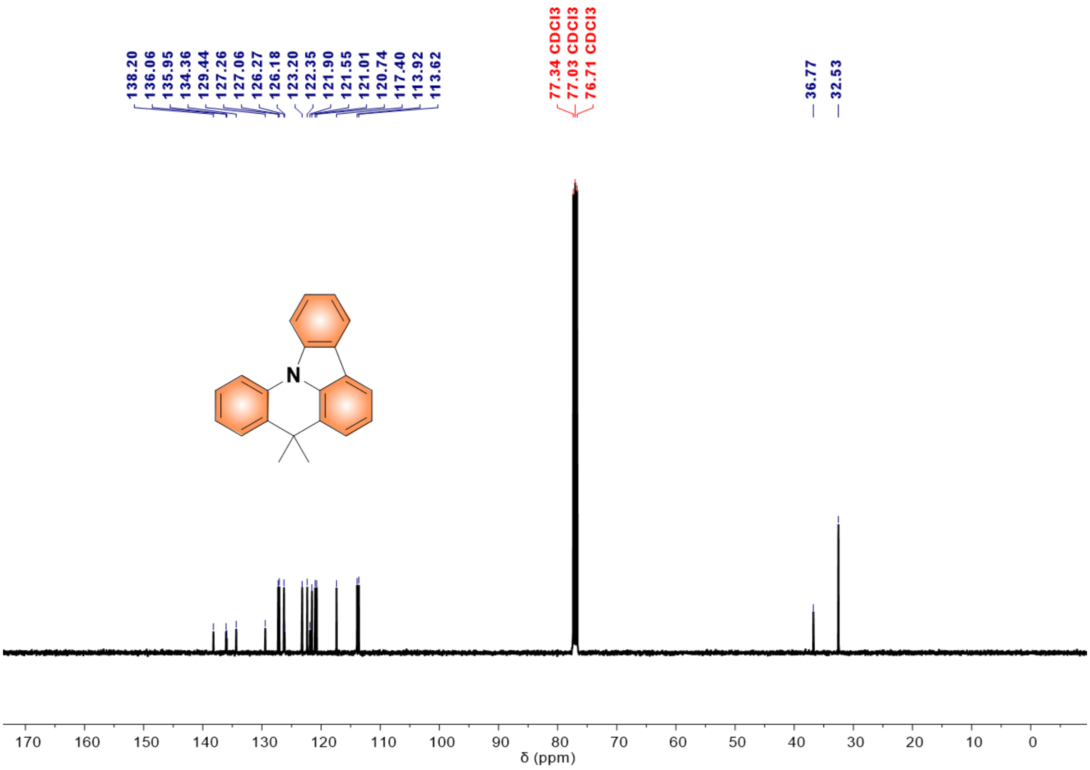

Figure S9. ^{13}C NMR（100 MHz) spectrum of 1,2-PhAc in $CDCl_3$.

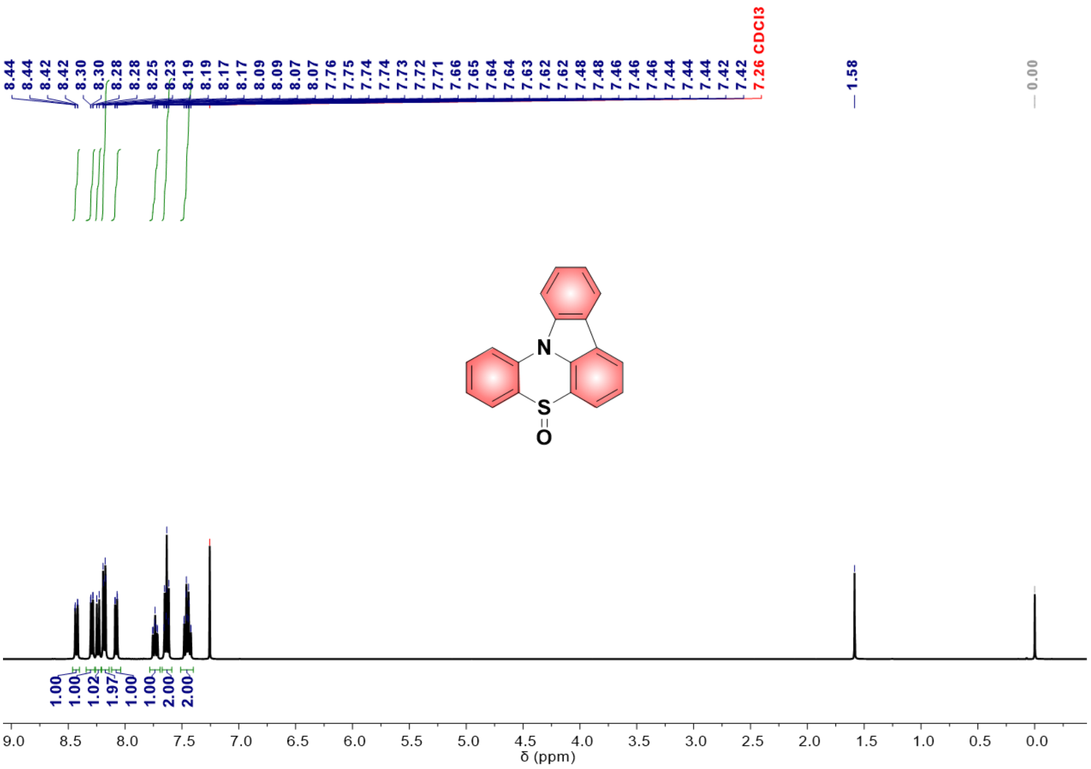

Figure S10. ^{1}H NMR（400 MHz) spectrum of 1,2-PhPTZO in $CDCl_3$.

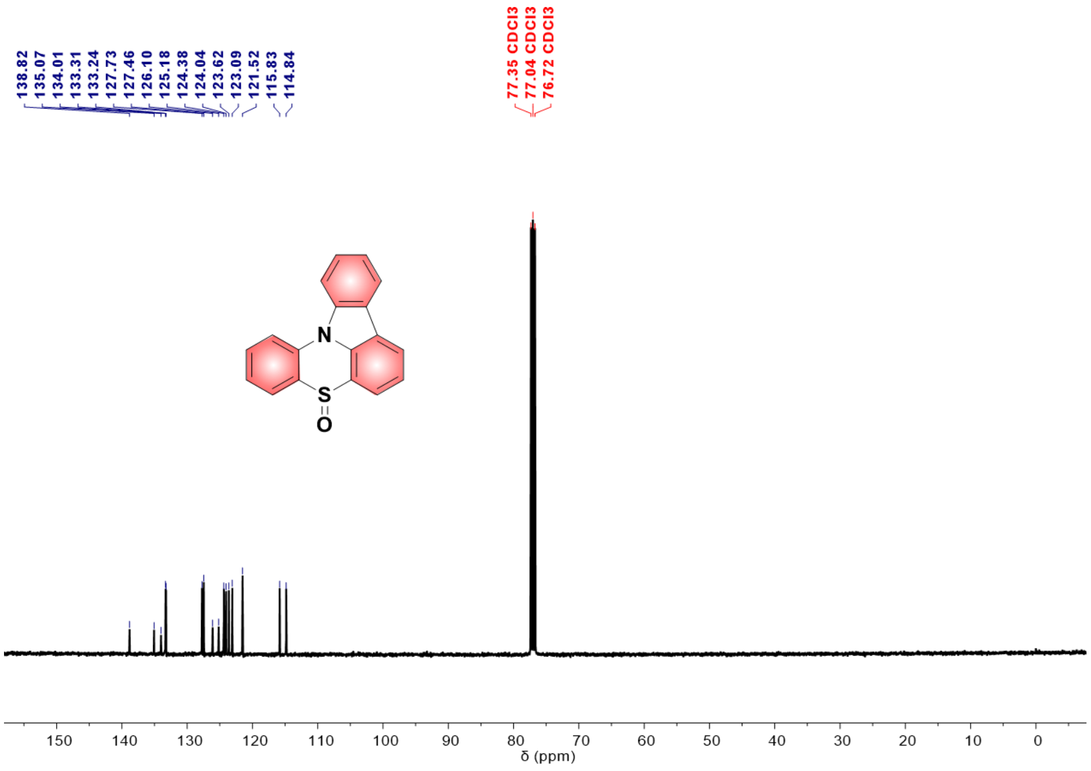

Figure S11. ^{13}C NMR（100 MHz) spectrum of 1,2-PhPTZO in $CDCl_3$.

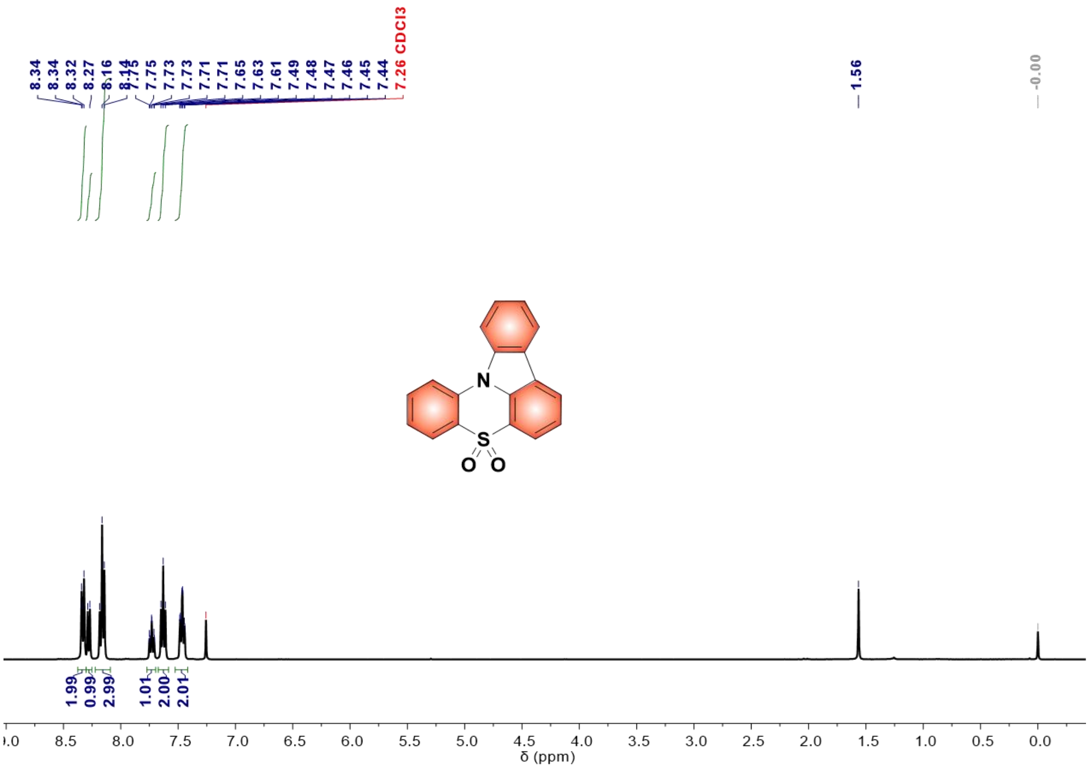

Figure S12. ^{1}H NMR（400 MHz) spectrum of 1,2-PhPTZOO in $CDCl_3$.

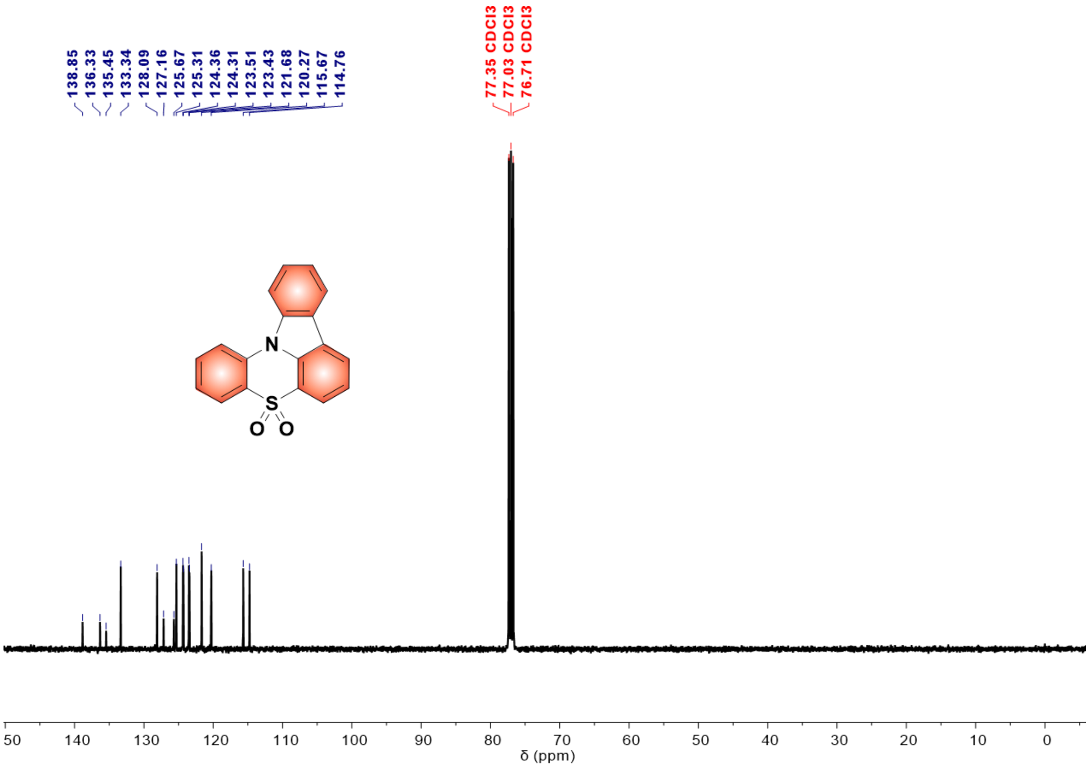

Figure S13. ^{13}C NMR（100 MHz) spectrum of 1,2-PhPTZOO in $CDCl_3$.

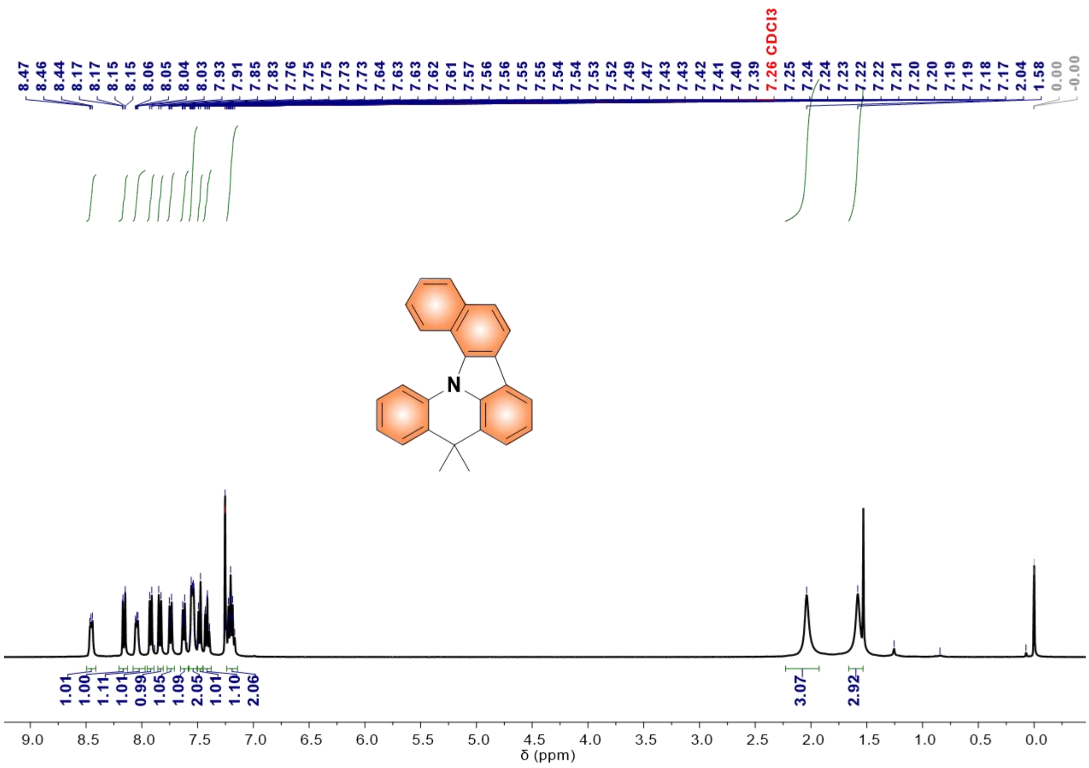

Figure S14. ^{1}H NMR（400 MHz) spectrum of 1,2-NpAc in $CDCl_3$.

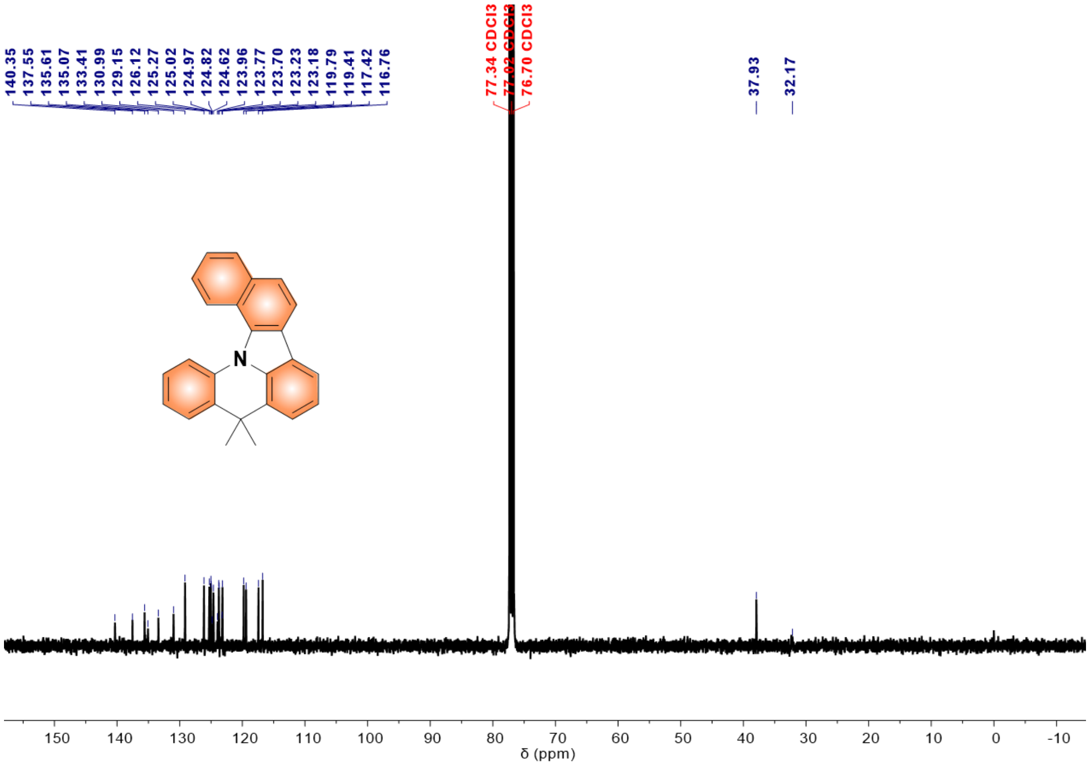

Figure S15. ^{13}C NMR（100 MHz) spectrum of 1,2-NpAc in $CDCl_3$.

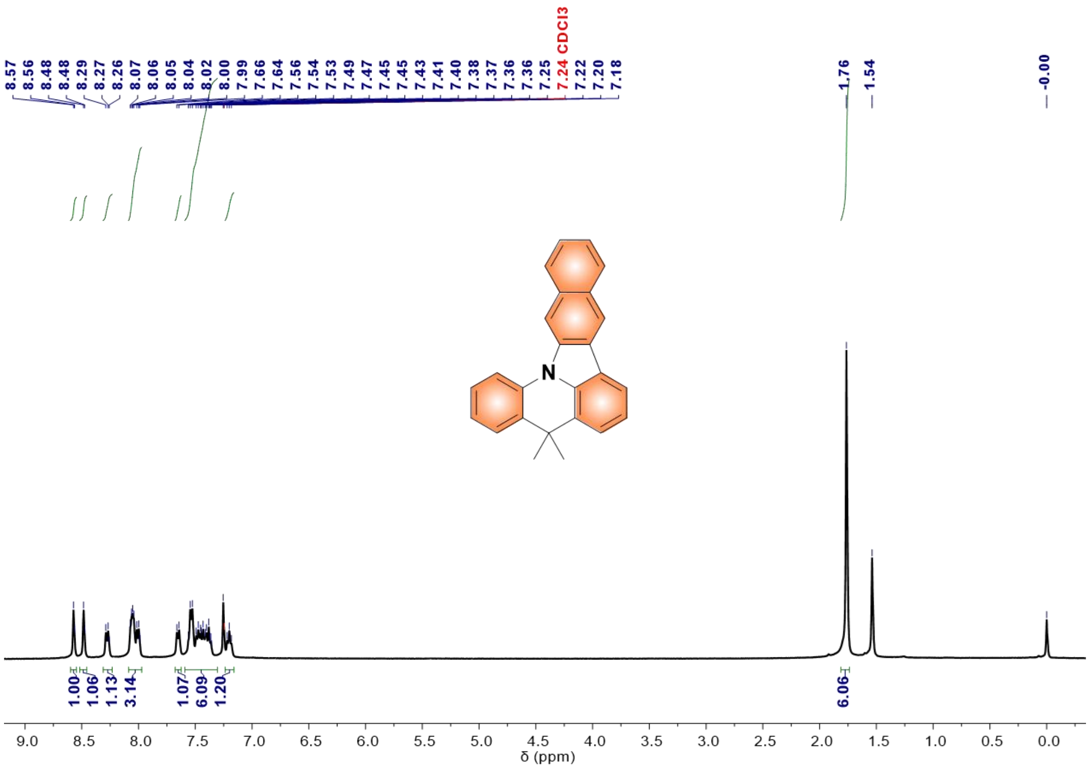

Figure S16. ^{1}H NMR（400 MHz) spectrum of 2,3-NpAc in $CDCl_3$.

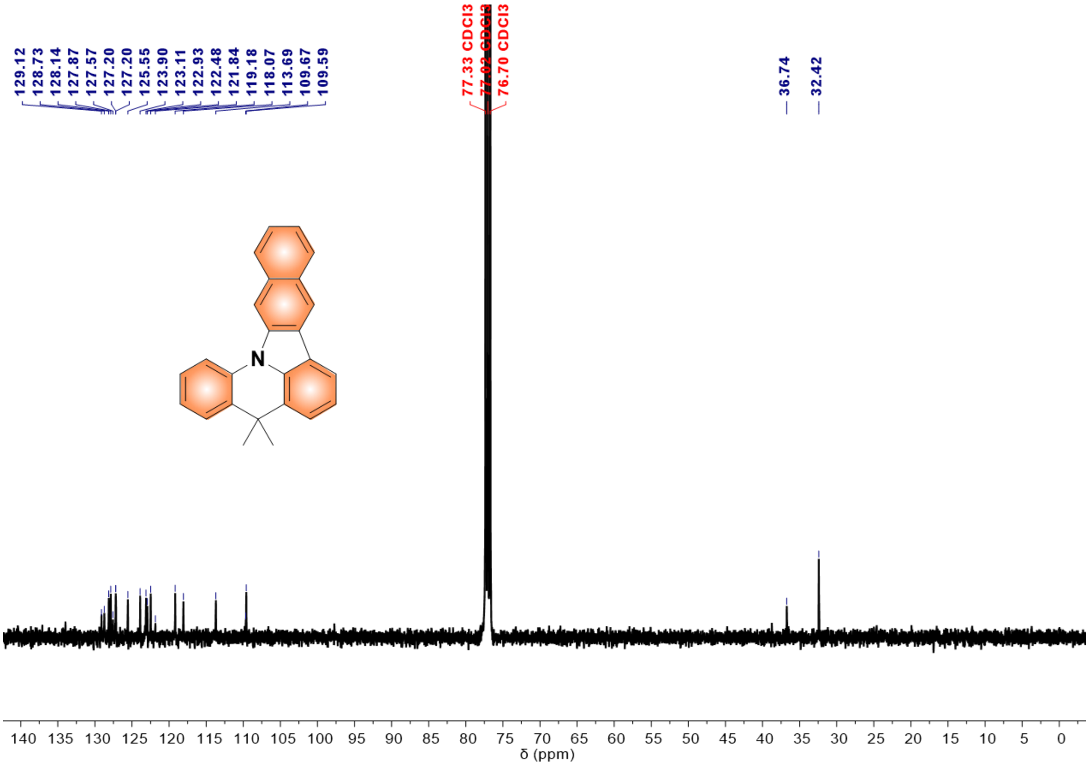

Figure S17. ^{13}C NMR（100 MHz) spectrum of 2,3-NpAc in $CDCl_3$.

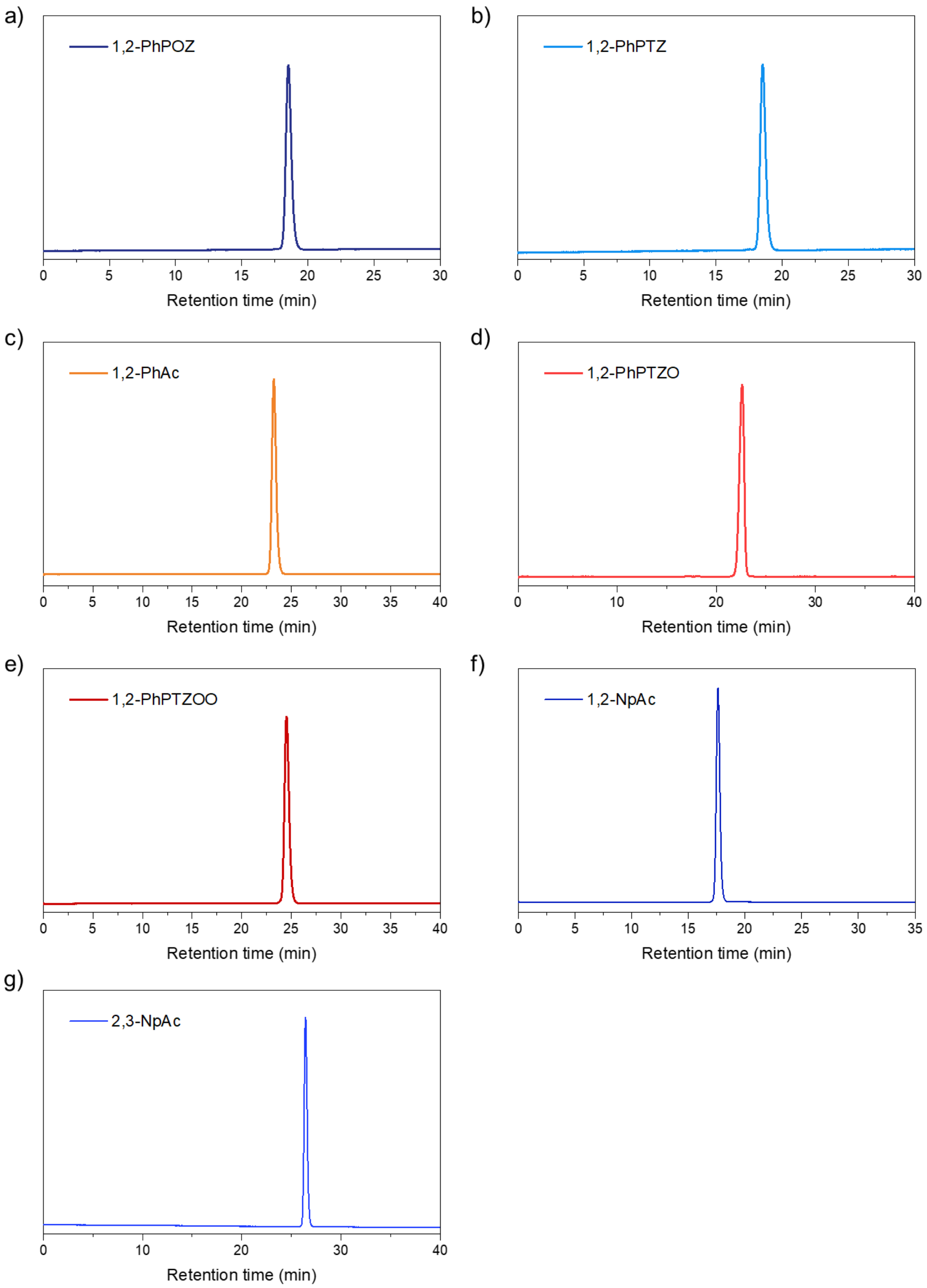

Figure S18. HPLC curves of 1,2-PhPOZ, 1,2-PhPTZ, 1,2-PhAc, 1,2-PhPTZO, 1,2-PhPTZOO, 1,2-NpAc, and 2,3-NpAc.

2.2 Photophysical property of luminogens

Table S3. Photophysical property of luminogens

Compound	λ_{abs} [a] (nm)	λ_F [a] (nm)	Φ_{PL} [a] (%)	λ_P [b] (nm)	λ_F[d] (nm)	λ_P [d] (nm)	τ_P [d] (ms)	Φ_{PL} [d] (%)
Ph-POZ	322	407	7.22	486	415	530	1.41	9.64
1,2-PhPOZ	370	405	42.84	470	435	610	0.89	42.60
Ph-PTZ	328	460	5.75	535	465	540	3.12	4.62
1,2-PhPTZ	358	415	8.75	515	460	530	5.95	7.45
Ph-Ac	285	355	7.59	425	350	505	157.70	10.92
1,2-PhAc	351	384	50.28	450	400	590	445.25	46.76
Ph-PTZO	335	370	7.17	410	390	525	24.52	0.85
1,2-PhPTZO	355	374	13.50	460	435	545	15.72	4.50
Ph-PTZOO	330	370	8.72	407	375	530	119.32	4.26
1,2-PhPTZOO	350	375	12.85	465	392	580	93.80	5.15

[a] The maximum absorption wavelengths of UV-vis absorption and maximum emission wavelengths of PL spectra in THF solution (10 μM) at room temperature, [b] The maximum emission wavelengths of phosphorescence spectra in THF solution (10 μM) at 77 K, [c] The maximum emission wavelengths of PL and phosphorescence spectra, phosphorescence lifetimes and PLQYs (including fluorescence and phosphorescence) in crystal state at room temperature. All the spectral and photophysical properties were obtained in air.

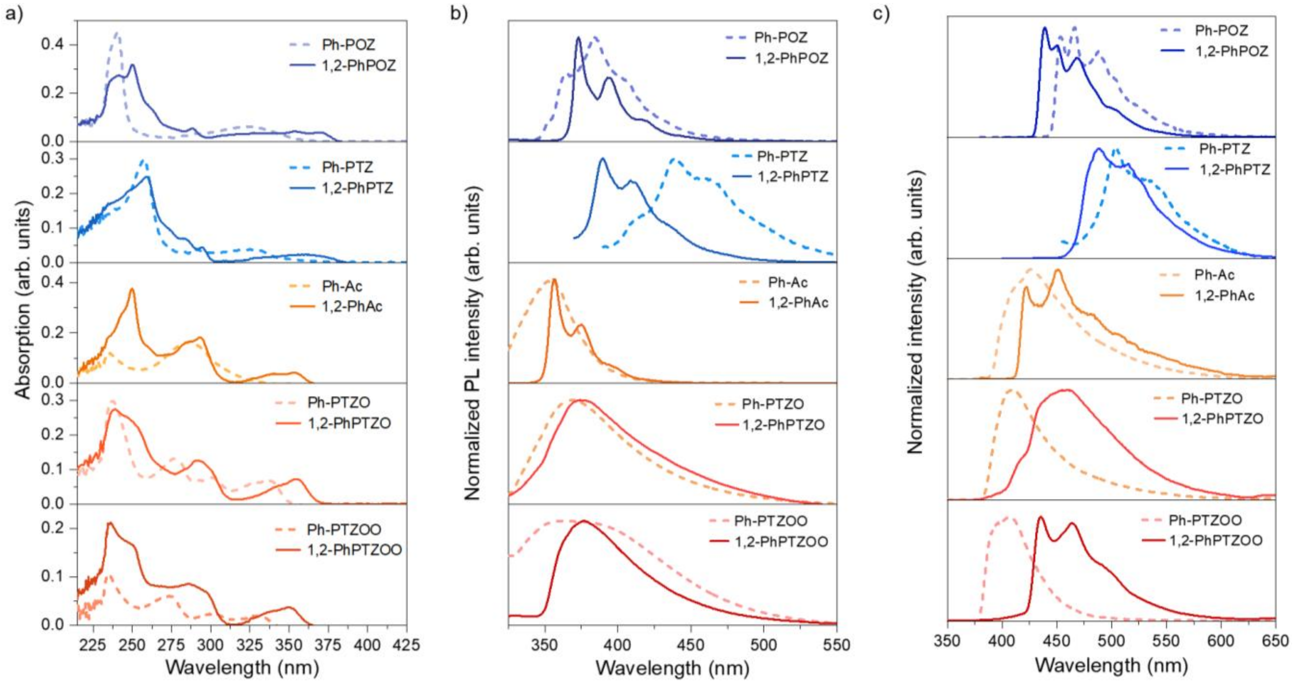

Figure S19. a) Absorption and b) PL spectra of Ph-POZ, Ph-PTZ, Ph-Ac, Ph-PTZO, Ph-PTZOO, 1,2-PhPOZ, 1,2-PhPTZ, 1,2-PhAc, 1,2-PhPTZO and 1,2-PhPTZOO (10 μM) in THF solution at room temperature, c) Phosphorescence spectra of Ph-POZ, Ph-PTZ, Ph-Ac, Ph-PTZO, Ph-PTZOO, 1,2-PhPOZ, 1,2-PhPTZ, 1,2-PhAc, 1,2-PhPTZO and 1,2-PhPTZOO (10 μM) in THF solution at 77 K.

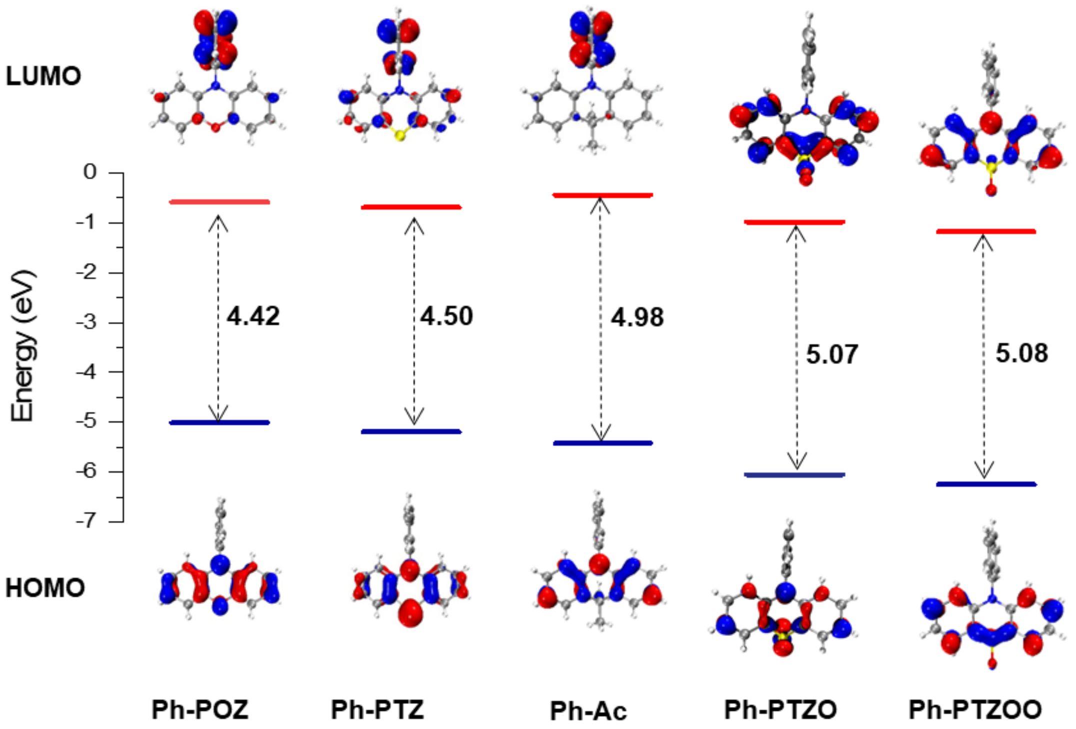

Figure S20. Molecular orbitals of Ph-POZ, Ph-PTZ, Ph-Ac, Ph-PTZO and Ph-PTZOO (The geometries of molecular configurations were extracted from single crystals), and the corresponding energy levels.

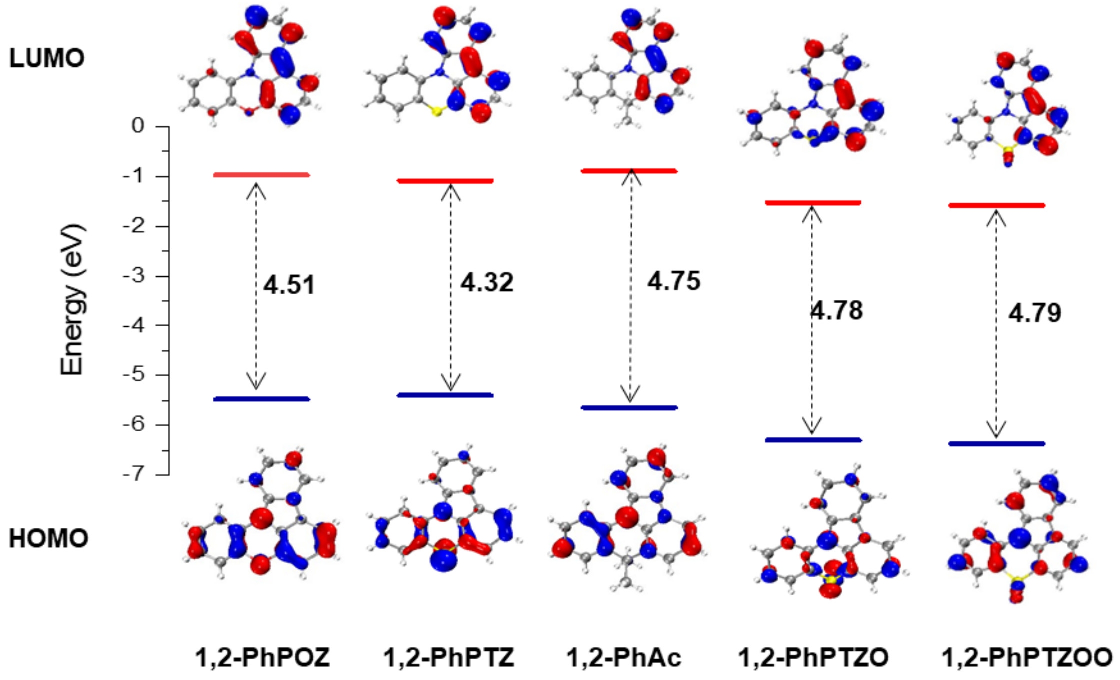

Figure S21. Molecular orbitals of 1,2-PhPOZ, 1,2-PhPTZ, 1,2-PhAc, 1,2-PhPTZO and 1,2-PhPTZOO (The geometries of molecular configurations were extracted from single crystals), and the corresponding energy levels.

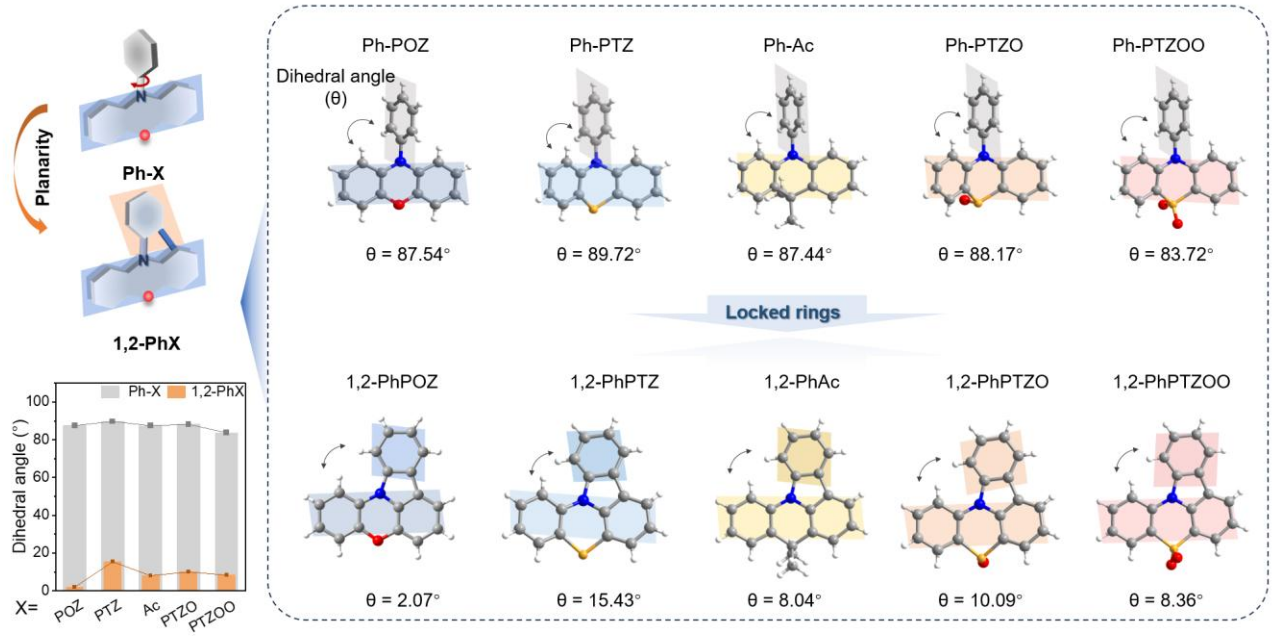

Figure S22. Molecular conformations and dihedral angles between phenyl and *aza*-polycyclic aromatic hydrocarbons in Ph-POZ, Ph-PTZ, Ph-Ac, Ph-PTZO, Ph-PTZOO, 1,2-PhPOZ, 1,2-PhPTZ, 1,2-PhAc, 1,2-PhPTZO and 1,2-PhPTZOO.

Solvatochromic effect investigation:

The influence of solvent environment on the optical property of our compounds can be used to probe the excited-state feature using the Lippert-Mataga equation[1-3], which is a model that describes the interactions between the solvent and the dipole moment of solute:

$$hc(V_a - V_f) = hc(v_a^0 - v_f^0) + \frac{2(\mu_e - \mu_g)^2}{a^3} f(\varepsilon, n) \qquad (1)$$

where $\boldsymbol{h}$ is Planck constant, c is the speed of light, $\boldsymbol{f}$ **(ε, n)** is the orientational polarizability of the solvents, $\mathbf{v_a}$ and v_f are absorption and emission peaks, $(\boldsymbol{v_a^0} - \boldsymbol{v_f^0})$ corresponds to the Stokes shifts when $\boldsymbol{f}$ **(ε, n)** is zero, $\boldsymbol{\mu_e}$ is the excited state dipole moment, and $\boldsymbol{\mu_g}$ is the ground-state dipole moment, $\boldsymbol{a}$ is the solvent cavity (Onsager) radius derived from the Avogadro number ($\boldsymbol{N_A}$), molecular weight ($\boldsymbol{M}$) and density ($\boldsymbol{d}$ selected from single crystal), $\boldsymbol{\varepsilon}$ and $\boldsymbol{n}$ are the solvent dielectric and the solvent refractive index, respectively. Further, $\boldsymbol{f}$ **(ε, n)** and $\boldsymbol{a}$ can be calculated in Equations 2 and 3, respectively. (***f*****(ε,n)**, calculated from the static dielectric constants (ε) and refractive indices (**n**) at 298 K (equation 2), following the treatment described by Reichardt (Solvents and Solvent Effects in Organic Chemistry).)

$$f(\varepsilon, n) = \frac{\varepsilon - 1}{2\varepsilon + 1} - \frac{n^2 - 1}{2n^2 + 1} \qquad (2)$$

$$a^3 = \frac{3M}{4N_A \pi d} \qquad (3)$$

$$\mu_e = \Delta\mu + \mu_g \qquad (4)$$

According to the reported article[4], the ground state dipole moment μ_g was computed by geometry optimization. Thus, μ_e can be calculated in Equations 4.

Table S4. Calculation of f (ε, n) in various solvents

Solvent	ε	n	f(ε, n)
Cyclohexane	1.88	1.375	-0.002
n-hexane	2.02	1.426	-0.001
Toluene	2.38	1.497	0.013
Chlorobenzene	5.62	1.524	0.143
Chloroform	6.02	1.372	0.150
Ethyl acetate	8.93	1.424	0.199
THF	4.89	1.446	0.210
Dichloromethane	7.58	1.407	0.217
DMF	36.71	1.430	0.274
Acetone	20.56	1.359	0.284
Acetonitrile	35.94	1.344	0.304

Table S5. Solvatochromic effect investigation

Solvent	f (ε, n)	ν_a-ν_f (cm^{-1})				
		1,2-PhPOZ	1,2-PhPTZ	1,2-PhAc	1,2-PhPTZO	1,2-PhPTZOO
Cyclohexane	-0.002	1637.722	3285.129	1894.284	851.3273	968.773
n-hexane	-0.001	1646.316	3362.936	1823.362	851.3273	890.529
Toluene	0.013	1809.651	3464.909	1954.064	1057.027	1174.168
Chlorobenzene	0.143	2107.426	3552.849	2059.354	1129.484	1235.178
Chloroform	0.150	2262.82	3739.36	2367.424	1280.236	1471.158
Ethyl acetate	0.199	2236.257	3661.116	2162.372	1294.41	1397.516
THF	0.210	2335.669	3836.575	2448.362	1431.046	1904.762
Dichloromethane	0.217	2274.552	3620.961	2299.43	1351.92	1471.158
DMF	0.274	2738.152	4045.629	2702.15	1502.347	2046.23
Acetone	0.284	2738.152	4009.516	2716.833	2127.347	2128.096
Acetonitrile	0.304	2798.079	4188.199	2783.088	2012.822	2210.433
a (Å)		4.34	4.24	4.43	4.30	4.35
$\Delta\mu = (\mu_e - \mu_g)^a$		6.7 D	5.4 D	6.5 D	6.5 D	7.1 D
$\Delta\mu = (\mu_e - \mu_g)^b$		-	3.6 / 6.4 D	4.3 / 7.7 D	4.6 / 8.2 D	4.8 / 8.7 D
μ_g		1.2 D	1.3 D	1.0 D	4.6 D	5.9 D
μ_e^a		7.9 D	6.7 D	7.5 D	11.1 D	13.0 D
μ_e^b		-	4.9 / 7.7 D	5.3 / 8.7 D	9.2 / 12.8 D	10.7 / 14.6 D

[a] Using single-linear fitting; [b] Using two-section linear fitting.

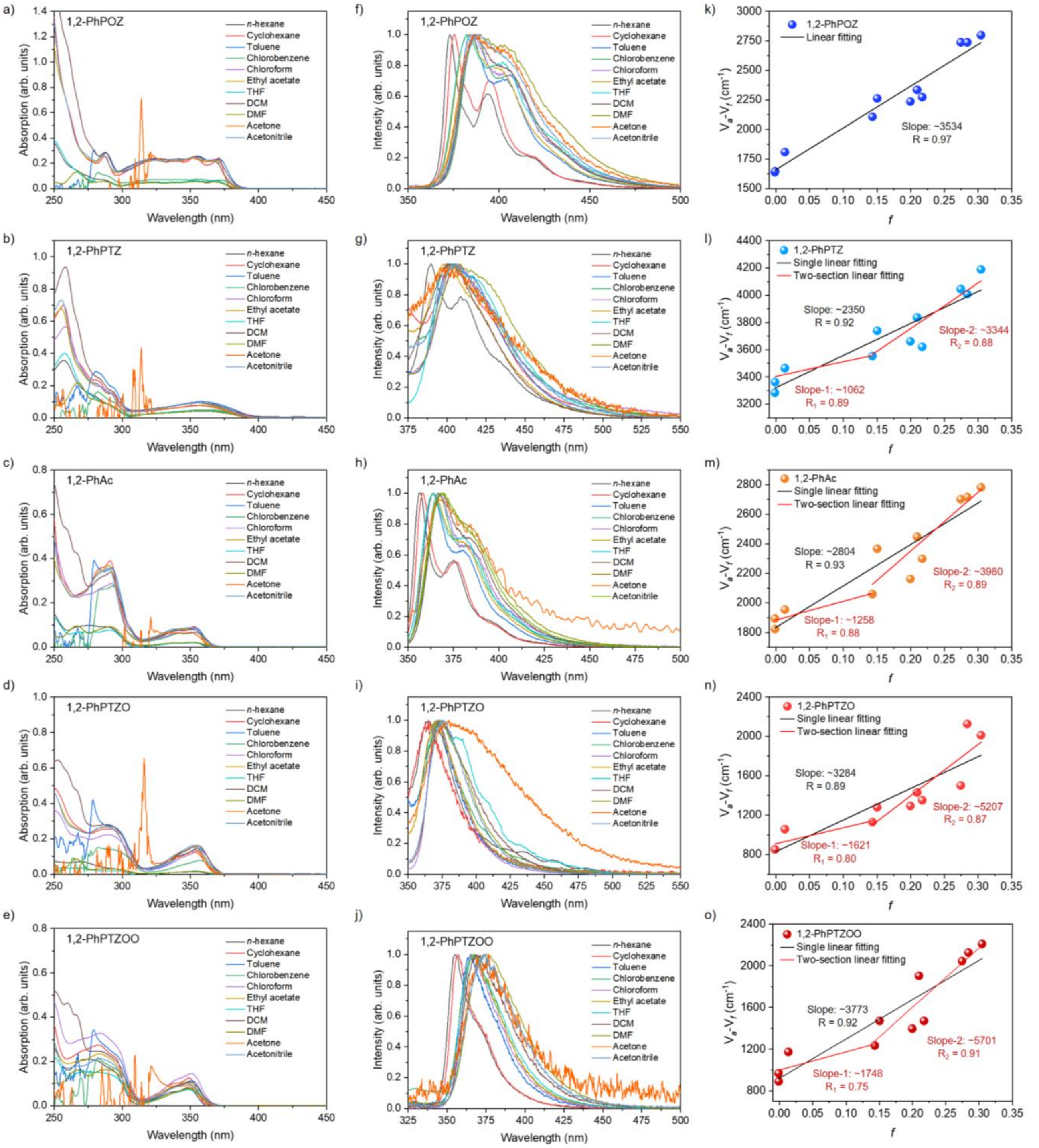

Figure S23. a-e) Absorption and f-j) PL spectra of 1,2-PhPOZ, 1,2-PhPTZ, 1,2-PhAc, 1,2-PhPTZO and 1,2-PhPTZOO (10 μM) in different solution at room temperature and k-o) The solvatochromic Lippert–Mataga models of 1,2-PhPOZ, 1,2-PhPTZ, 1,2-PhAc, 1,2-PhPTZO and 1,2-PhPTZOO (V_a-V_f = $(1/\lambda_{abs} - 1/\lambda_{PL}) * 10^7$).(THF: tetrahydrofuran; DCM: dichloromethane; DMF: *N,N*-Dimethylformamide)

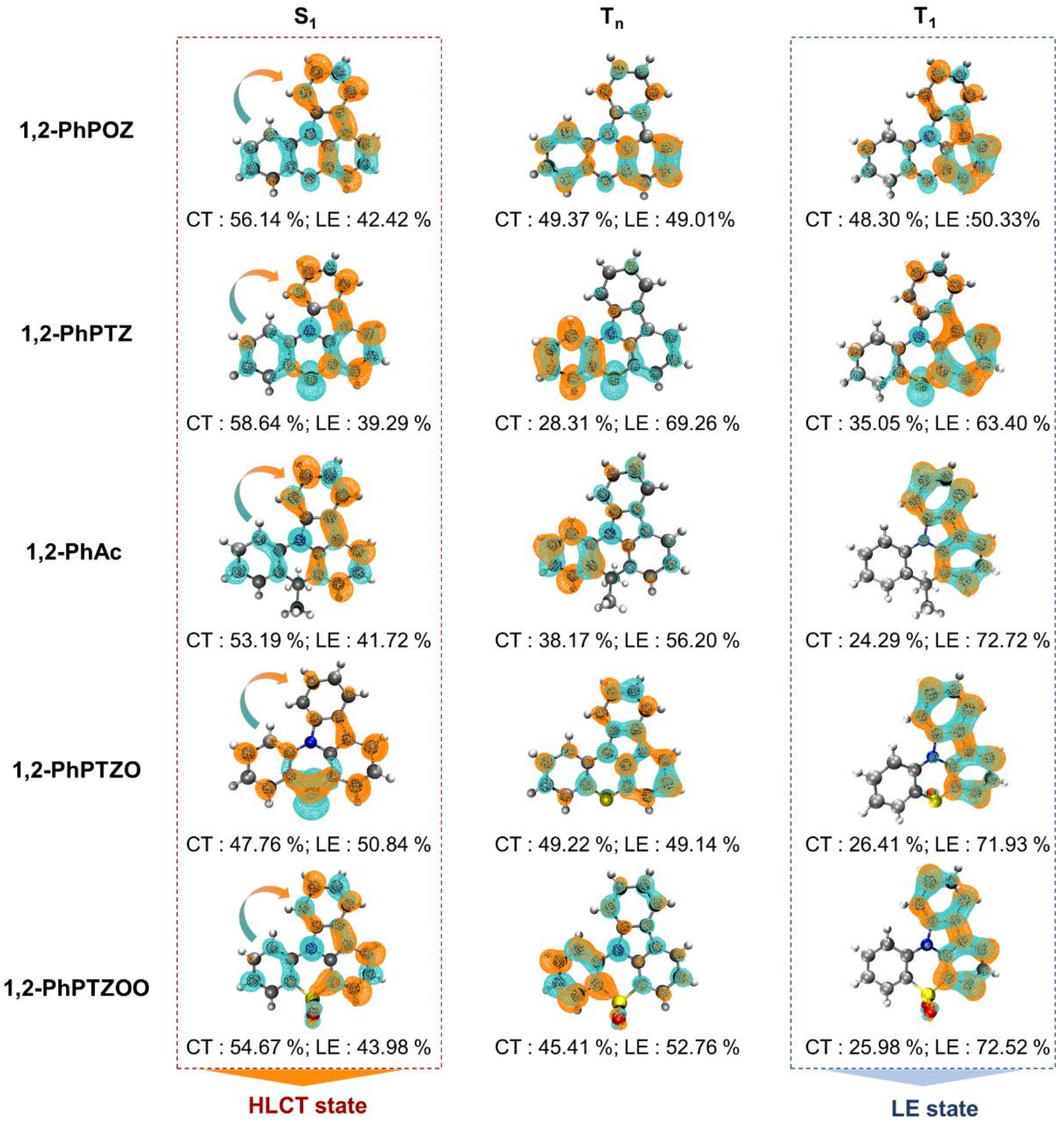

Figure S24. Isosurface plots (isovalue = 0.0020) of electron-hole distributions in the S_1, T_n and T_1 states of 1,2-PhPOZ, 1,2-PhPTZ, 1,2-PhAc, 1,2-PhPTZO and 1,2-PhPTZOO (The geometries of molecular orbitals were extracted from single crystals, CT: Charge Transfer, LE: Locally Excited, HLCT: Hybridized Local and Charge-transfer)

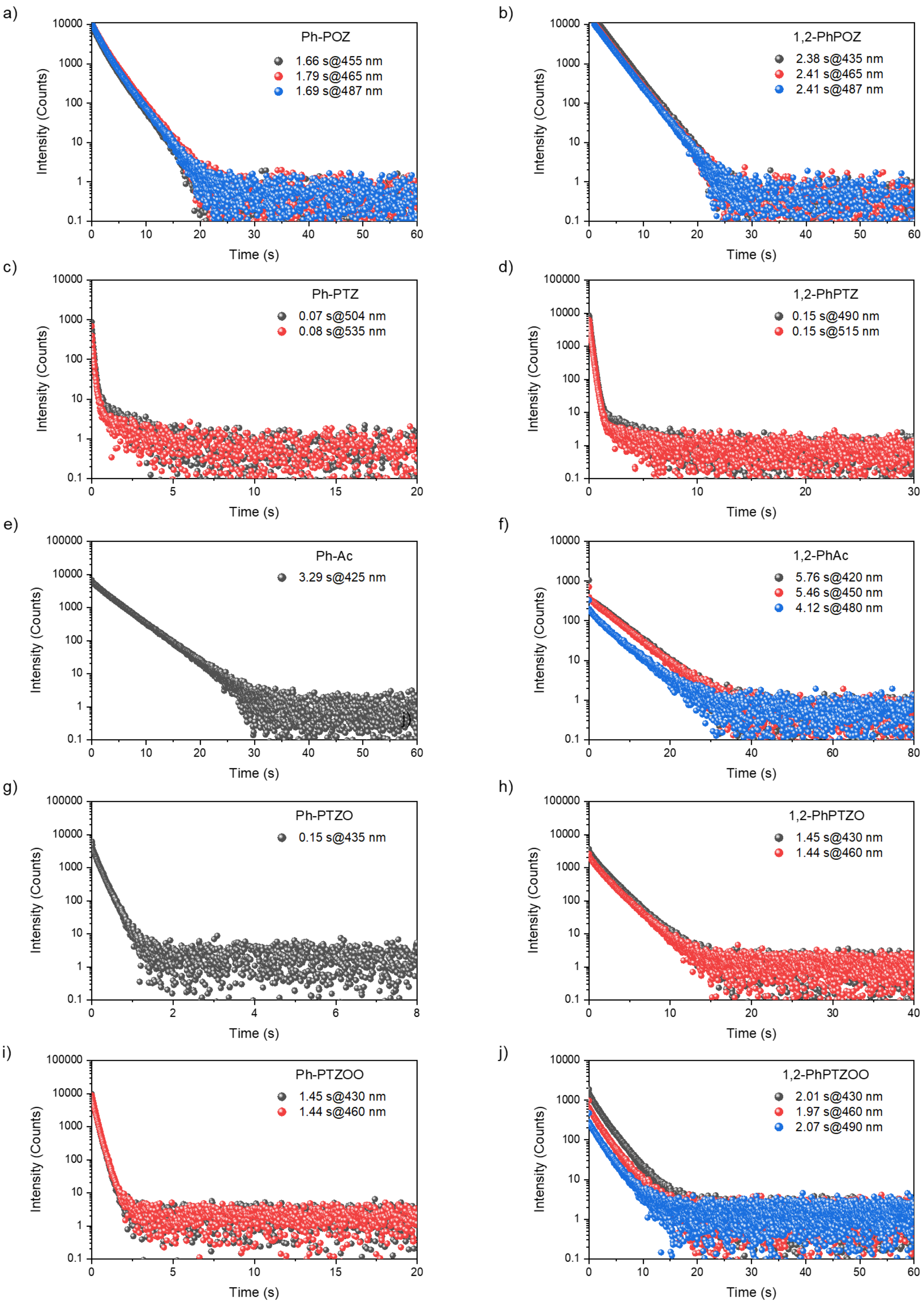

Figure S25. The phosphorescence decays of a) Ph-POZ, b) 1,2-PhPOZ, c) Ph-PTZ, d) 1,2-PhPTZ, e) Ph-Ac, f) 1,2-PhAc, g) Ph-PTZO, h) 1,2-PhPTZO, i) Ph-PTZOO, and j) 1,2-PhPTZOO (10 μM) in THF solution at 77 K.

Table S6. Exponential fitting parameters of the delayed emission decay in THF at 77 K

Sample	τ_1 (s)	Rel.%	τ (s)	χ^2
Ph-POZ@455 nm	1.66	100	1.66	0.999
Ph-POZ@465 nm	1.79	100	1.79	0.999
Ph-POZ@487 nm	1.69	100	1.69	0.999
1,2-PhPOZ@435 nm	2.38	100	2.38	0.999
1,2-PhPOZ@465 nm	2.41	100	2.41	0.998
1,2-PhPOZ@487 nm	2.41	100	2.41	0.999
Ph-PTZ@504 nm	0.07	100	0.07	0.988
Ph-PTZ@535 nm	0.08	100	0.08	0.989
1,2-PhPTZ@490 nm	0.15	100	0.15	0.998
1,2-PhPTZ@515 nm	0.15	100	0.15	0.999
Ph-Ac@425 nm	3.29	100	3.29	0.999
1,2-PhAc@420 nm	5.18	100	5.18	0.998
1,2-PhAc@450 nm	5.46	100	5.46	0.987
1,2-PhAc@480 nm	4.12	100	4.12	0.980
Ph-PTZO@435 nm	0.15	100	0.15	0.999
1,2-PhPTZO@430 nm	1.45	100	1.45	0.997
1,2-PhPTZO@460 nm	1.44	100	1.44	0.997
Ph-PTZOO@430 nm	1.45	100	1.45	0.999
Ph-PTZOO@460 nm	1.44	100	1.44	0.999
1,2-PhPTZOO@430 nm	2.01	100	2.01	0.996
1,2-PhPTZOO@460 nm	1.97	100	1.97	0.998
1,2-PhPTZOO@490 nm	2.07	100	2.07	0.998

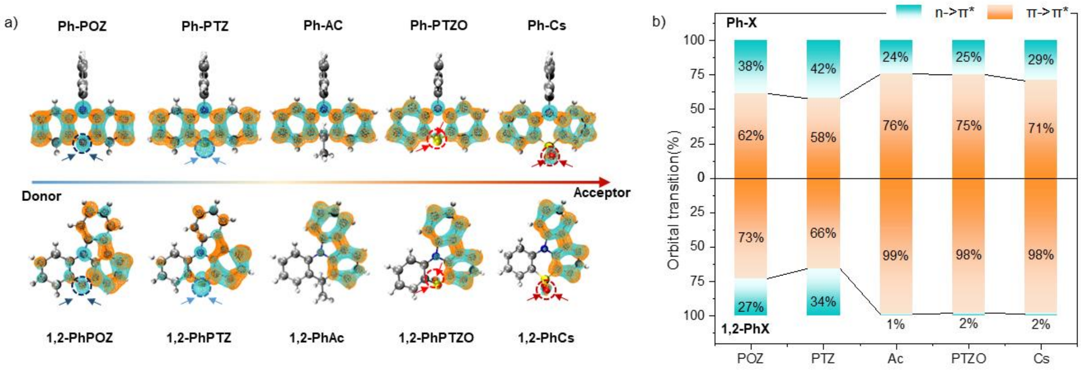

Figure S26. a) Isosurface plots (isovalue = 0.0020) of electron-hole distributions in the excited triplet states of Ph-POZ, Ph-PTZ, Ph-Ac, Ph-PTZO, Ph-PTZOO, 1,2-PhPOZ, 1,2-PhPTZ, 1,2-PhAc, 1,2-PhPTZO and 1,2-PhPTZOO (The geometries of molecular configurations were extracted from single crystals). b) The comparison of the proportions of (π, π*) character in the excited triplet state.

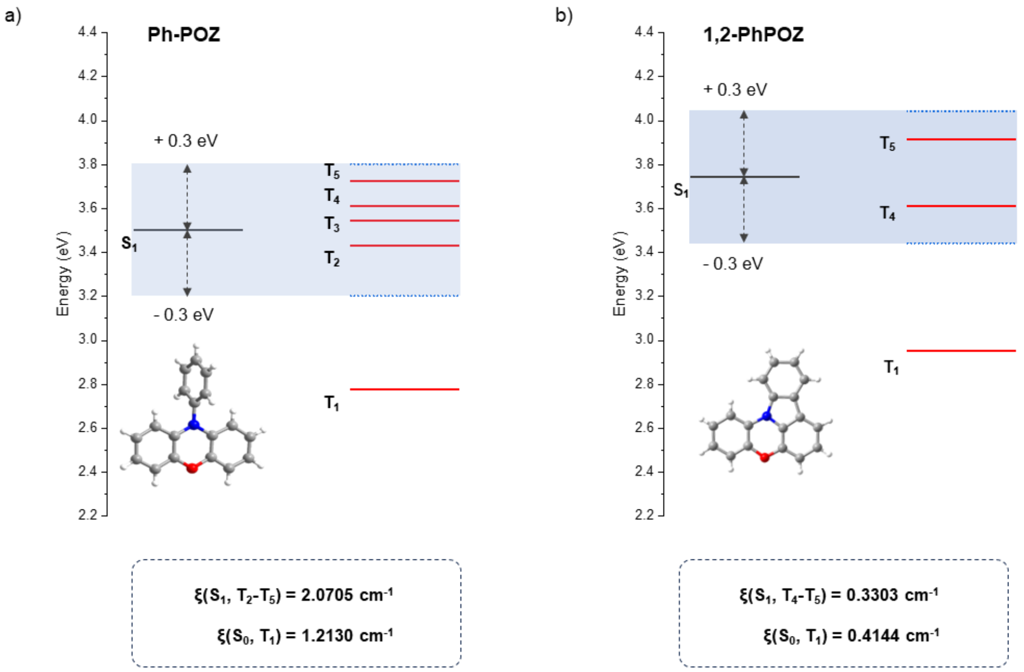

Figure S27. Calculated spin-orbit coupling (SOC) constants and energy gaps of a) Ph-POZ and b) 1,2-PhPOZ for the intersystem crossing process. (The molecular geometries were extracted from the corresponding single crystals)

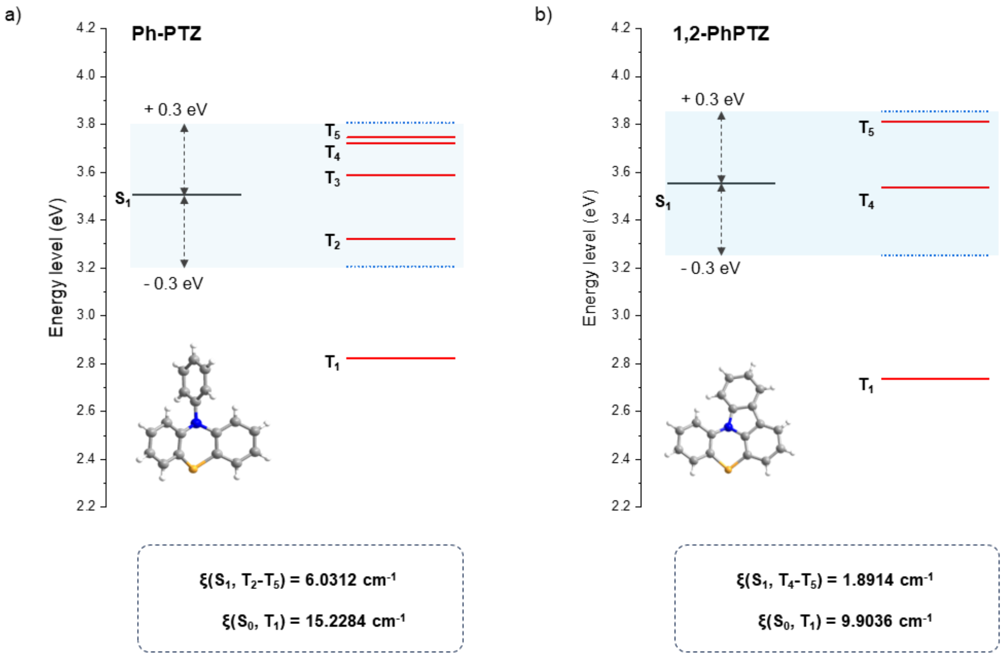

Figure S28. Calculated spin-orbit coupling (SOC) constants and energy gaps of a) Ph-PTZ and b) 1,2-PhPTZ for the intersystem crossing process. (The molecular geometries were extracted from the corresponding single crystals)

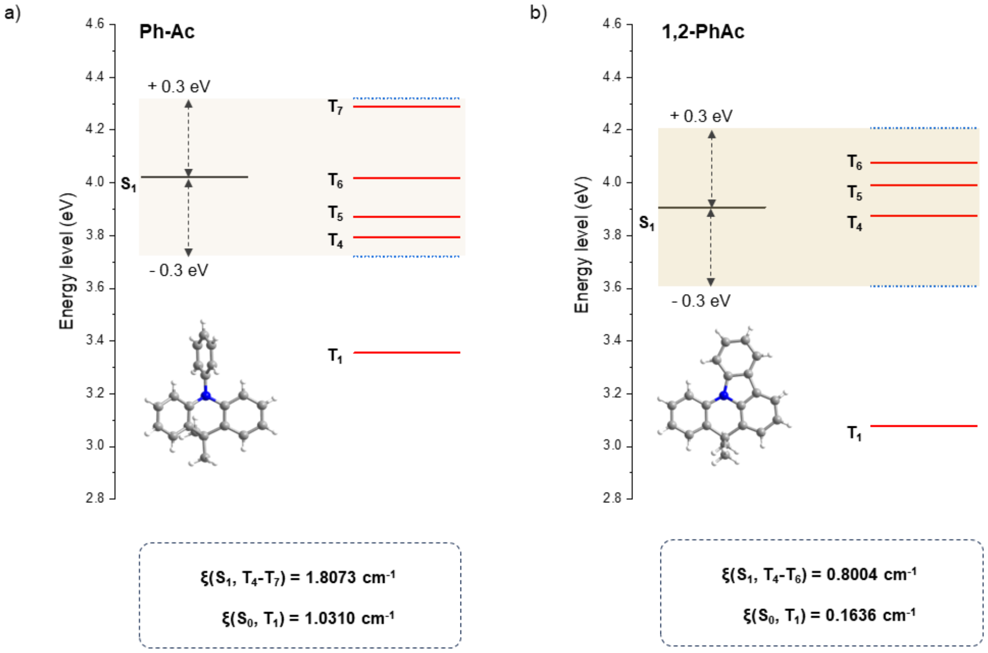

Figure S29. Calculated spin-orbit coupling (SOC) constants and energy gaps of a) Ph-Ac and b) 1,2-PhAc for the intersystem crossing process. (The molecular geometries were extracted from the corresponding single crystals)

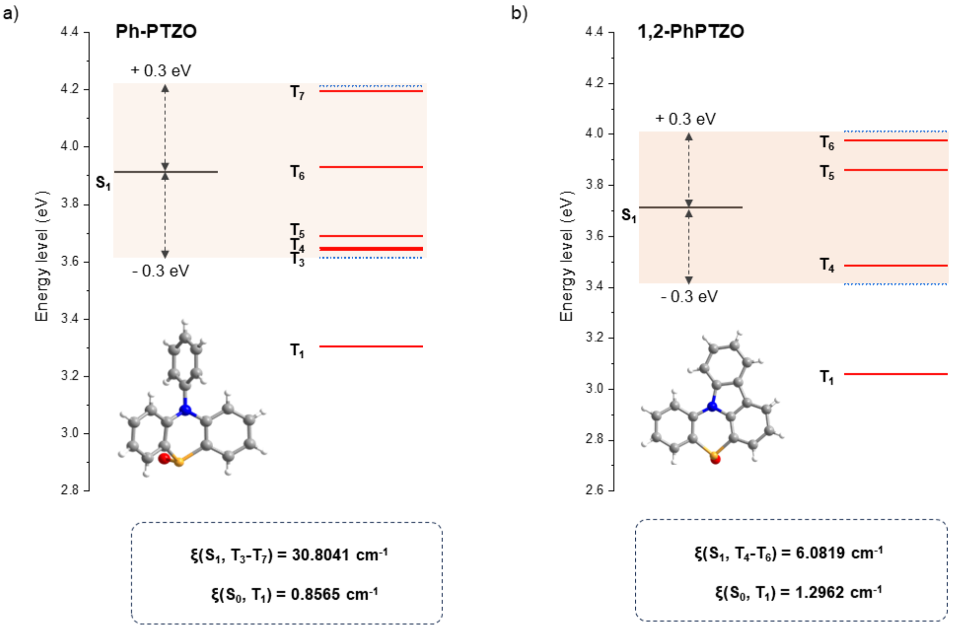

Figure S30. Calculated spin-orbit coupling (SOC) constants and energy gaps of a) Ph-PTZO and b) 1,2-PhPTZO for the intersystem crossing process. (The molecular geometries were extracted from the corresponding single crystals)

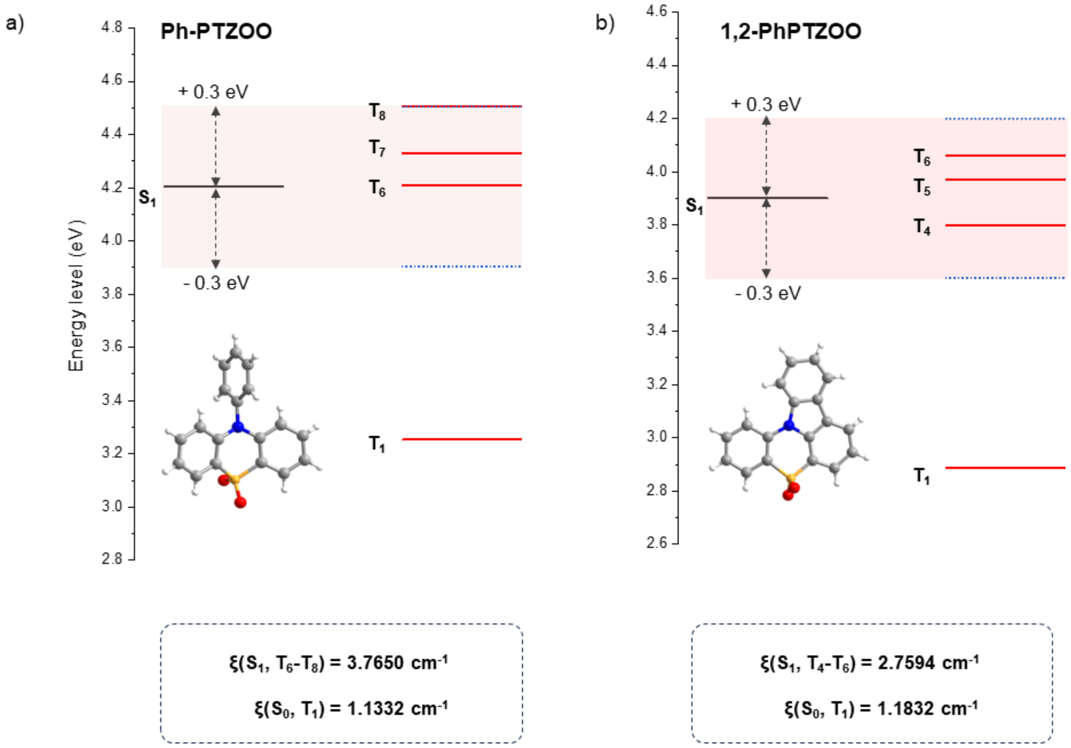

Figure S31. Calculated spin-orbit coupling (SOC) constants and energy gaps of a) Ph-PTZOO and b) 1,2-PhPTZOO for the intersystem crossing process. (The molecular geometries were extracted from the corresponding single crystals)

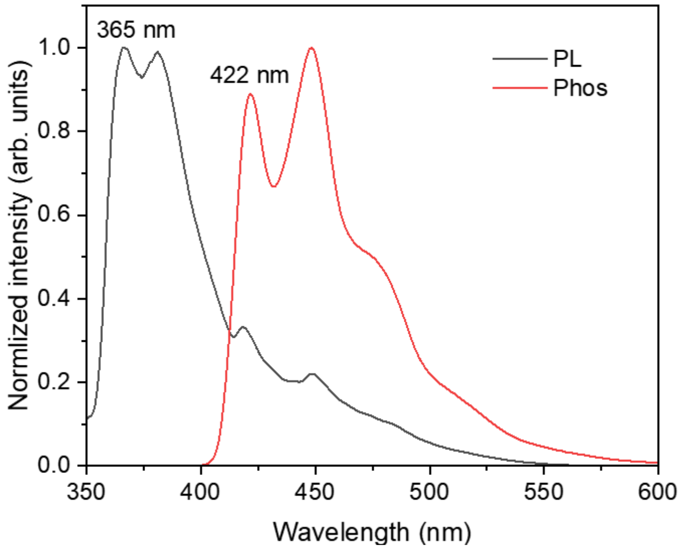

Figure S32. The photoluminescence (PL) and phosphorescence (Phos) spectra of 1,2-PhAc in THF solution at 77 K.

Table S7. The energy level and SOC of Ph-POZ and 1,2-PhPOZ.

Ph-POZ				1,2-PhPOZ			
	n-th	Energy level (eV)	ξ (S_1, T_n)		n-th	Energy level (eV)	ξ (S_1, T_n)
S_n	1	3.5024		S_n	1	3.7433	
T_n	1	2.7776		T_n	1	2.9552	
	2	3.4300	0.0138		2	3.0954	
	3	3.5433	0.4497		3	3.2185	
	4	3.6121	1.4119		4	3.6140	0.03148
	5	3.7272	0.1950		5	3.9159	0.29886
	6	3.8208			6	4.1698	

Table S8. The energy level and SOC of Ph-PTZ and 1,2-PhPTZ.

Ph-PTZ				1,2-PhPTZ			
	n-th	Energy level (eV)	ξ (S_1, T_n)		n-th	Energy level (eV)	ξ (S_1, T_n)
S_n	1	3.5068		S_n	1	3.5537	
T_n	1	2.8232		T_n	1	2.7374	
	2	3.3215	4.9381		2	3.0315	
	3	3.5865	0.2202		3	3.1914	
	4	3.7202	0.7252		4	3.5358	1.4164
	5	3.7450	0.1478		5	3.8136	0.4750
	6	3.8650			6	4.1075	

Table S9. The energy level and SOC of Ph-Ac and 1,2-PhAc.

Ph-Ac				1,2-PhAc			
	n-th	Energy level (eV)	ξ (S_1, T_n)		n-th	Energy level (eV)	ξ (S_1, T_n)
S_n	1	4.0215		S_n	1	3.9071	
T_n	1	3.3546		T_n	1	3.0775	
	2	3.6497			2	3.2526	
	3	3.7112			3	3.3291	
	4	3.7928	0.1953		4	3.8768	0.2857
	5	3.8723	0.6470		5	3.9911	0.2681
	6	4.0197	0.6389		6	4.0750	0.2467
	7	4.2905	0.3261		7	4.4363	
	8	4.4157					

Table S10. The energy level and SOC of Ph-PTZO and 1,2-PhPTZO.

Ph-PTZO				1,2-PhPTZO			
	n-th	Energy level (eV)	ξ (S_1, T_n)		n-th	Energy level (eV)	ξ (S_1, T_n)
S_n	1	3.9143		S_n	1	3.7136	
T_n	1	3.3053		T_n	1	3.0617	
	2	3.5035			2	3.3509	
	3	3.6402	5.9007		3	3.4023	
	4	3.6475	3.2634		4	3.4837	0.9602
	5	3.6914	4.7770		5	3.8604	0.9449
	6	3.9305	2.0542		6	3.9769	4.1768
	7	4.1958	14.8087		7	4.0696	
	8	4.2242					

Table S11. The energy level and SOC of Ph-PTZOO and 1,2-PhPTZOO.

Ph-PTZOO				1,2-PhPTZOO			
	n-th	Energy level (eV)	ξ (S_1, T_n)		n-th	Energy level (eV)	ξ (S_1, T_n)
S_n	1	4.2042		S_n	1	3.9004	
T_n	1	3.2530		T_n	1	2.8874	
	2	3.5064			2	3.3195	
	3	3.5951			3	3.3601	
	4	3.6162			4	3.7983	2.2513
	5	3.8736			5	3.9697	0.1303
	6	4.2091	2.5244		6	4.0613	0.3779
	7	4.3289	0.7864		7	4.2869	
	8	4.5069	0.4542				
	9	4.5358					

2.3 Crystal data and theoretical calculation of luminogens

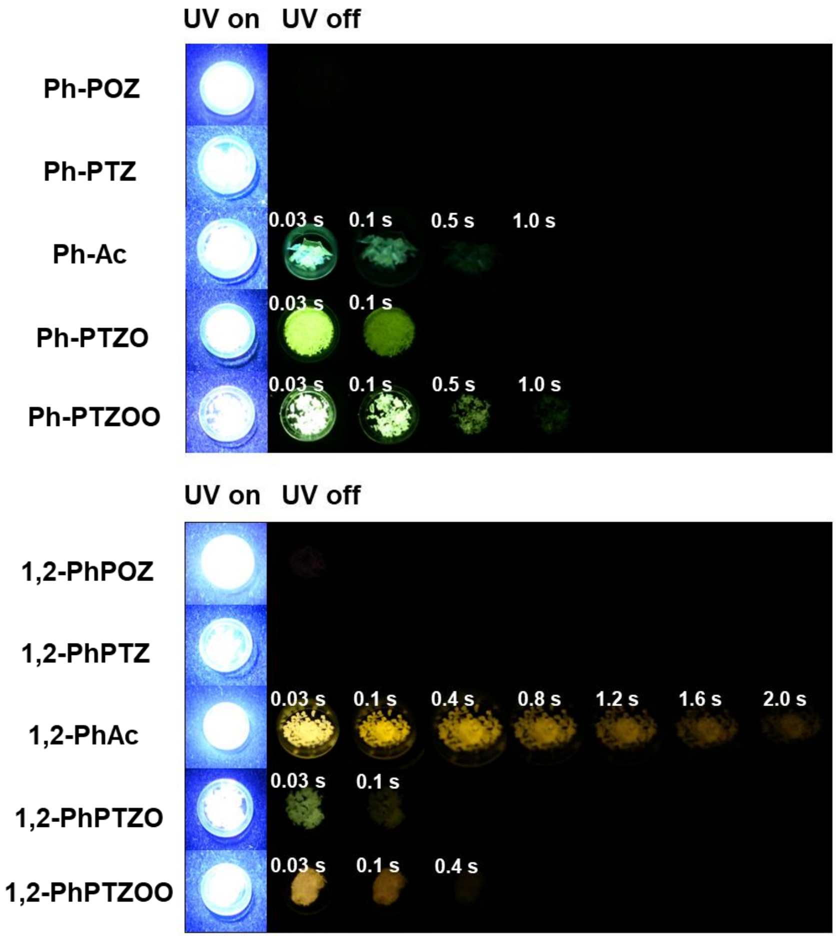

Figure S33. The photographs of Ph-POZ, Ph-PTZ, Ph-Ac, Ph-PTZO, Ph-PTZOO, 1,2-PhPOZ, 1,2-PhPTZ, 1,2-PhAc, 1,2-PhPTZO and 1,2-PhPTZOO crystals. The experiment was repeated independently 3 times with similar results.

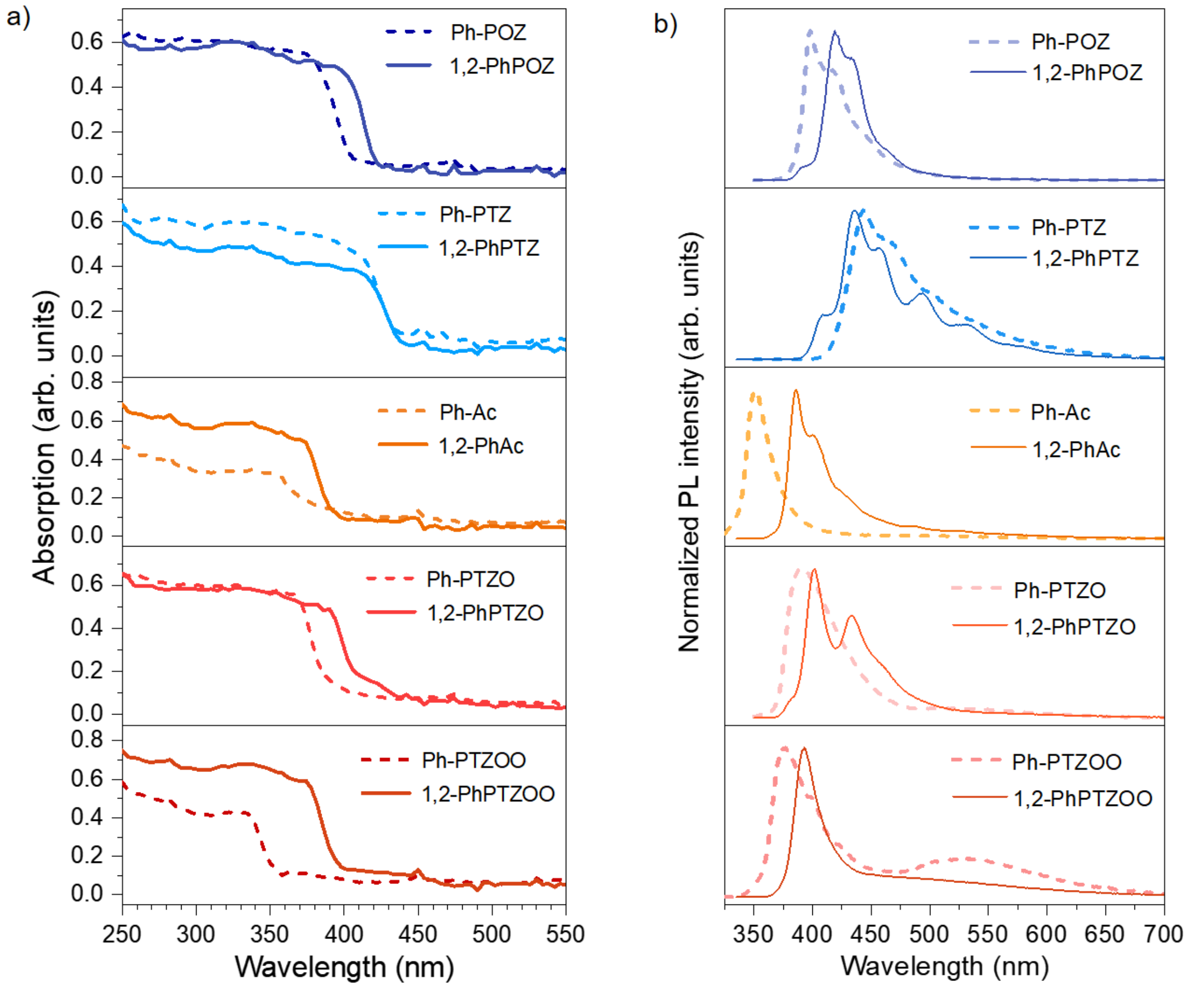

Figure S34. The a) absorption and b) PL spectra of Ph-POZ, Ph-PTZ, Ph-Ac, Ph-PTZO, Ph-PTZOO, 1,2-PhPOZ, 1,2-PhPTZ, 1,2-PhAc, 1,2-PhPTZO and 1,2-PhPTZOO crystals.

The crystal data of Ph-POZ, Ph-PTZ, Ph-Ac, and Ph-PTZOO could be obtained by the Cambridge Crystallographic Data Centre (CCDC), the CCDC number is 1022191, 2022480, 2042373 and 1519540, respectively.

Table S12. Crystal data of Ph-PTZO, 1,2-PhPOZ and 1,2-PhPTZ

Name	Ph-PTZO	1,2-PhPOZ	1,2-PhPTZ
Formula	$C_{18}H_{13}NOS$	$C_{18}H_{11}NO$	$C_{18}H_{11}NS$
Crystal system	Triclinic	Monoclinic	Orthorhombic
Space Group	P-1	P2(1)/n	Pbca
Cell Length (Å)	a = 7.976(4)<br>b = 8.007(3)<br>c = 12.741(6)	a = 15.232(2)<br>b = 4.980(6)<br>c = 16.677(2)	a = 16.298(4)<br>b = 7.733(19)<br>c = 20.826(6)
Cell Angles (°)	α = 101.300(2)<br>β = 97.465(2)<br>γ = 113.129(2)	α = 90<br>β = 102.945(12)<br>γ = 90	α = 90<br>β = 90<br>γ = 90
Cell Volume ($Å^3$)	714.21(6)	1232.9(3)	2624.77(13)
z	2	4	8
Density (g/cm^3)	1.355	1.386	1.383
F (000)	304	536	1136
h_{max}, k_{max}, l_{max}	$-10 \leq h \leq 11$,<br>$-9 \leq k \leq 11$,<br>$-16 \leq l \leq 18$	$-17 \leq h \leq 18$,<br>$-5 \leq k \leq 5$,<br>$-20 \leq l \leq 20$	$-20 \leq h \leq 14$,<br>$-9 \leq k \leq 9$,<br>$-25 \leq l \leq 25$
CCDC number	2491655	2491658	2491666

Table S13. Crystal data of 1,2-PhAc 1,2-PhPTZO and 1,2-PhPTZOO

Name	1,2-PhAc	1,2-PhPTZO	1,2-PhPTZOO
Formula	$C_{21}H_{17}N$	$C_{18}H_{11}NOS$	$C_{18}H_{11}NO_2S$
Crystal system	Orthorhombic	Orthorhombic	Monoclinic
Space Group	P212121	Pna21	P21/c
Cell Length (Å)	a = 8.916(3)	a = 9.234(4)	a = 7.675(5)
	b = 11.458(4)	b = 10.853(4)	b = 21.547(12)
	c = 14.441(5)	c = 13.381(6)	c = 16.974(10)
Cell Angles (°)	α = 90	α = 90	α = 90
	β = 90	β = 90	β = 99.428(6)
	γ = 90	γ = 90	γ = 90
Cell Volume ($Å^3$)	1475.36(9)	1341.06(10)	2769.00(3)
z	4	4	4
Density (g/cm^3)	1.276	1.433	1.470
F (000)	600	6004	1268
h_{max}, k_{max}, l_{max}	$-11 \le h \le 11$, $-14 \le k \le 14$, $-18 \le l \le 18$	$-11 \le h \le 11$, $-13 \le k \le 13$, $-16 \le l \le 13$	$-9 \le h \le 8$, $-26 \le k \le 24$, $-20 \le l \le 21$
CCDC number	2491716	2491717	2491719

Table S14. Multiexponential fitting parameters of the delayed emission decay in crystal state

Sample	τ_1 (ms)	Rel.%	τ_2 (ms)	Rel.%	τ_3 (ms)	Rel.%	τ (ms)	χ^2
Ph-POZ@492 nm	0.09	9.96	2.43	90.04	-	-	2.19	0.969
Ph-POZ@530 nm	0.15	30.53	1.96	69.47	-	-	1.41	0.923
1,2-PhPOZ@490 nm	0.15	15.68	2.45	54.67	23.31	29.64	8.27	1.099
1,2-PhPOZ@610 nm	0.01	0.28	3.51	56.87	19.37	23.76	0.89	1.155
Ph-PTZ@540 nm	1.75	60.42	5.21	39.58	-	-	3.12	0.997
1,2-PhPTZ@462 nm	4.77	90.77	19.56	9.23	-	-	6.14	1.016
1,2-PhPTZ@496 nm	3.45	60.27	8.92	39.73	-	-	5.62	0.989
1,2-PhPTZ@530 nm	3.02	51.75	9.08	48.25	-	-	5.95	1.041
Ph-Ac@505 nm	36.92	49.81	277.56	50.19	-	-	157.70	1.032
1,2-PhAc@590 nm	158.21	768.54	52.97	47.03	-	-	445.25	1.065
Ph-PTZO@525 nm	18.62	48.10	79.97	20.03			24.52	0.997
1,2-PhPTZO@495 nm	19.33	80.43	161.98	19.57	-	-	47.25	1.057
1,2-PhPTZO@535 nm	10.45	73.92	30.66	26.08	-	-	15.72	0.964
Ph-PTZOO@530 nm	35.84	330.6	71.68	28.32	-	-	119.32	1.031
1,2-PhPTZOO@580 nm	16.71	153.04	43.45	56.55	-	-	93.80	1.075

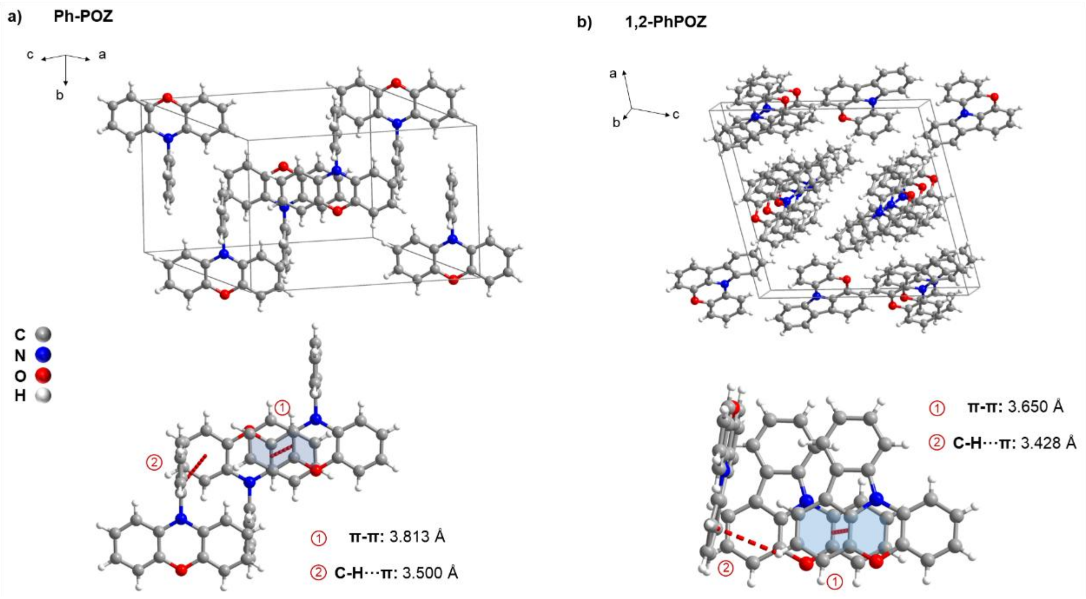

Figure S35. Packing modes and intermolecular interactions of Ph-POZ and 1,2-PhPOZ crystals.

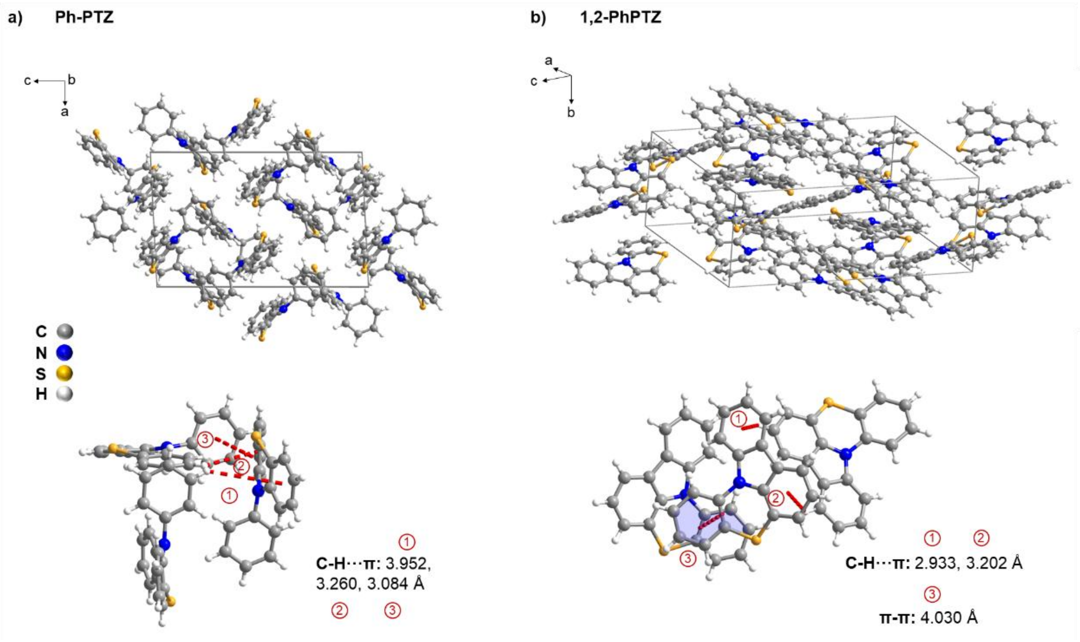

Figure S36. Packing modes and intermolecular interactions of Ph-PTZ and 1,2-PhPTZ crystals.

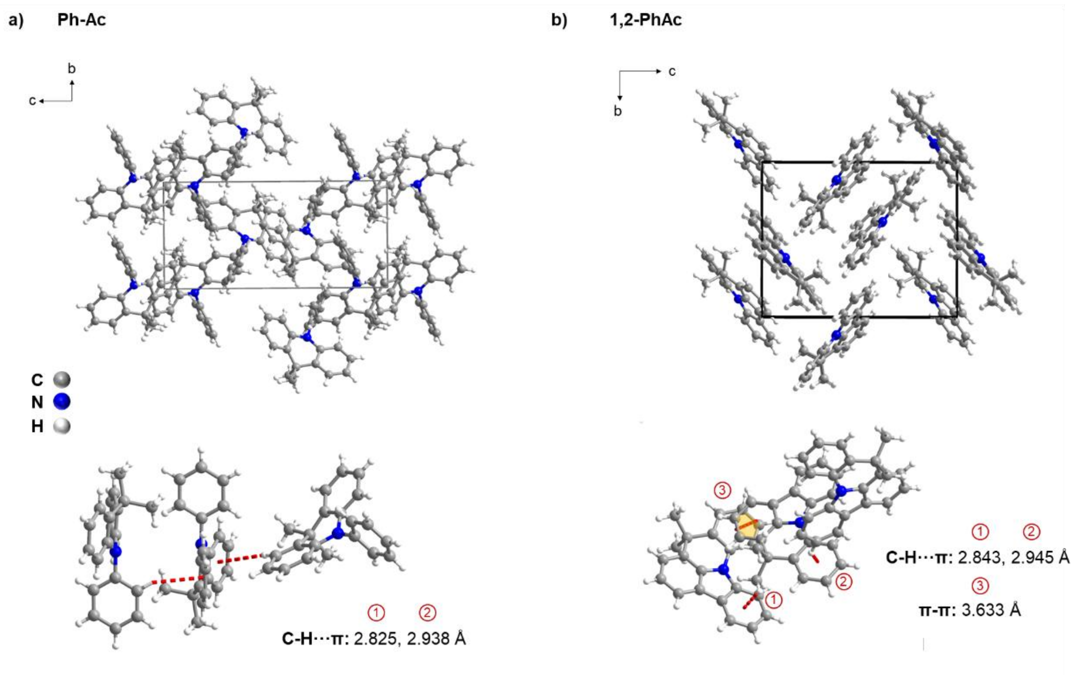

Figure S37. Packing modes and intermolecular interactions of Ph-Ac and 1,2-PhAc crystals.

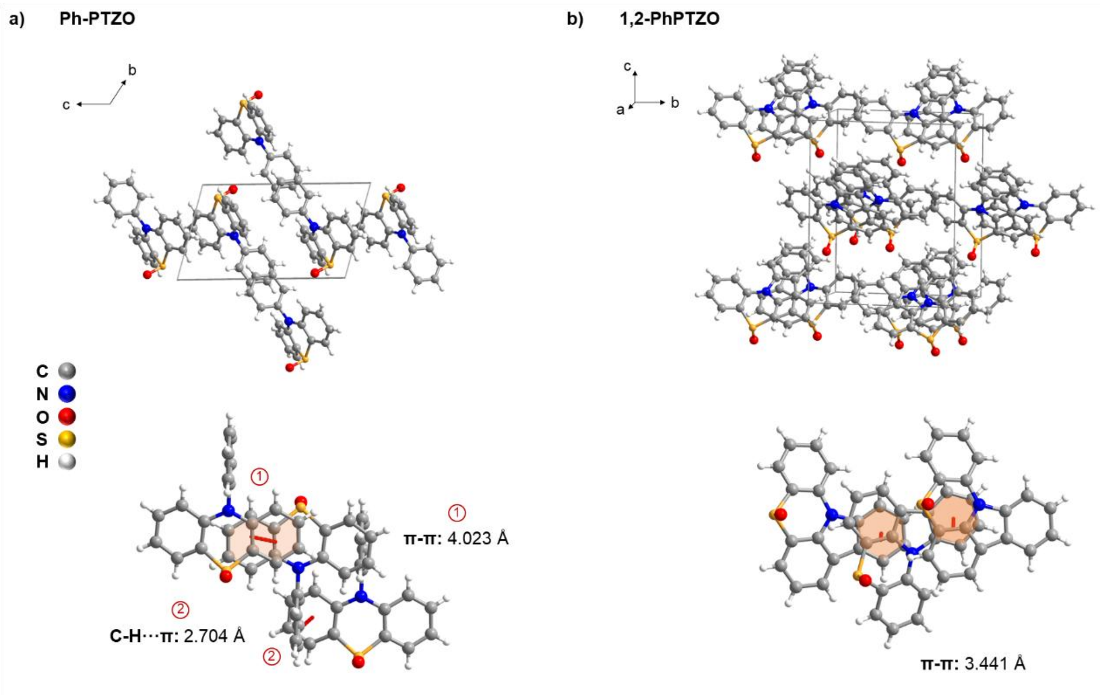

Figure S38. Packing modes and intermolecular interactions of Ph-PTZO and 1,2-PhPTZO crystals.

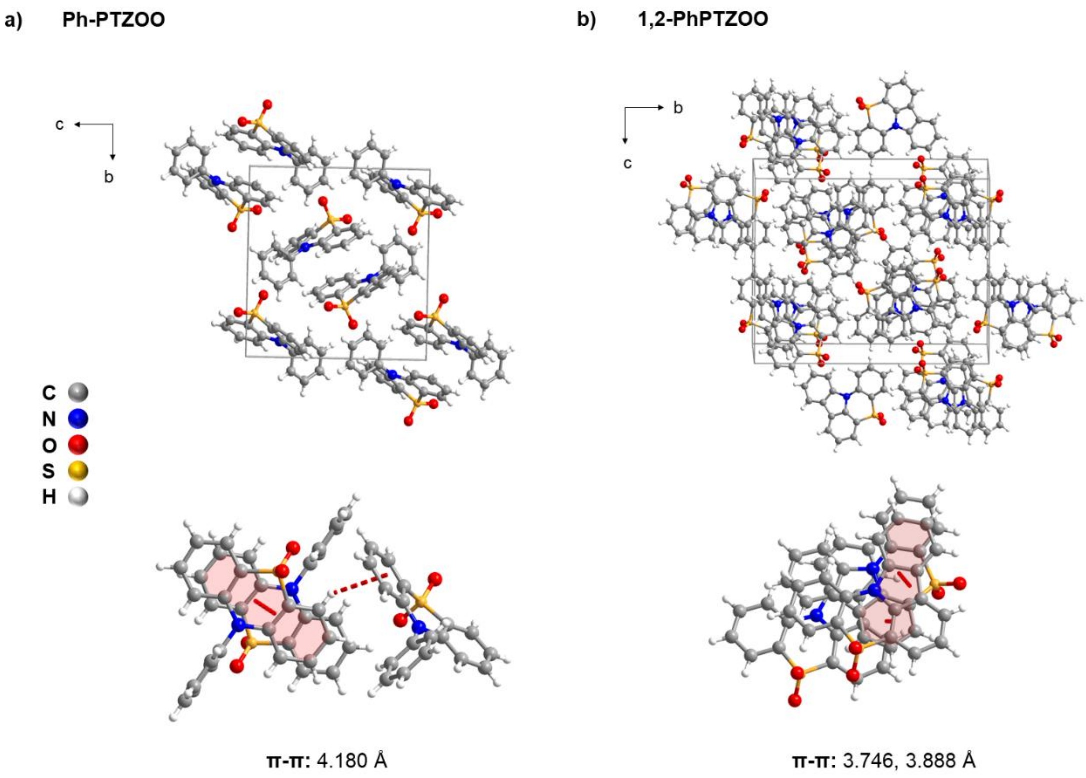

Figure S39. Packing modes and intermolecular interactions of Ph-PTZOO and 1,2-PhPTZOO crystals.

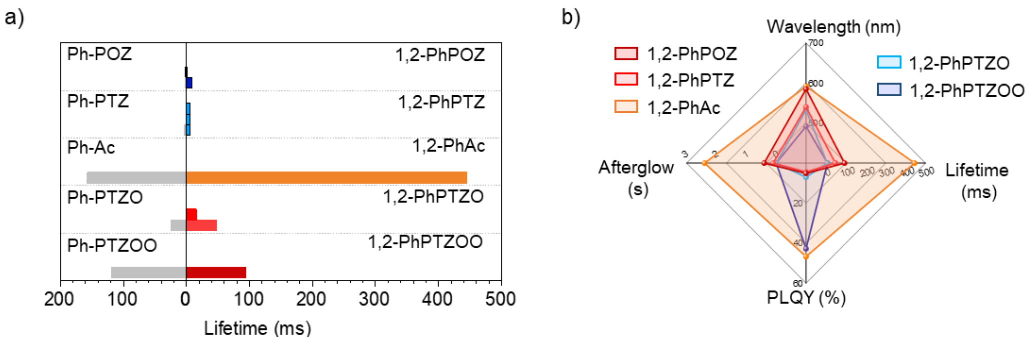

Figure S40. a) Delayed emission lifetimes of Ph-POZ, Ph-PTZ, Ph-Ac, Ph-PTZO, Ph-PTZOO 1,2-PhPOZ, 1,2-PhPTZ, 1,2-PhAc, 1,2-PhPTZO and 1,2-PhPTZOO crystals. b) Radar plot comparing the emission properties of 1,2-PhPOZ, 1,2-PhPTZ, 1,2-PhAc, 1,2-PhPTZO and 1,2-PhPTZOO crystals

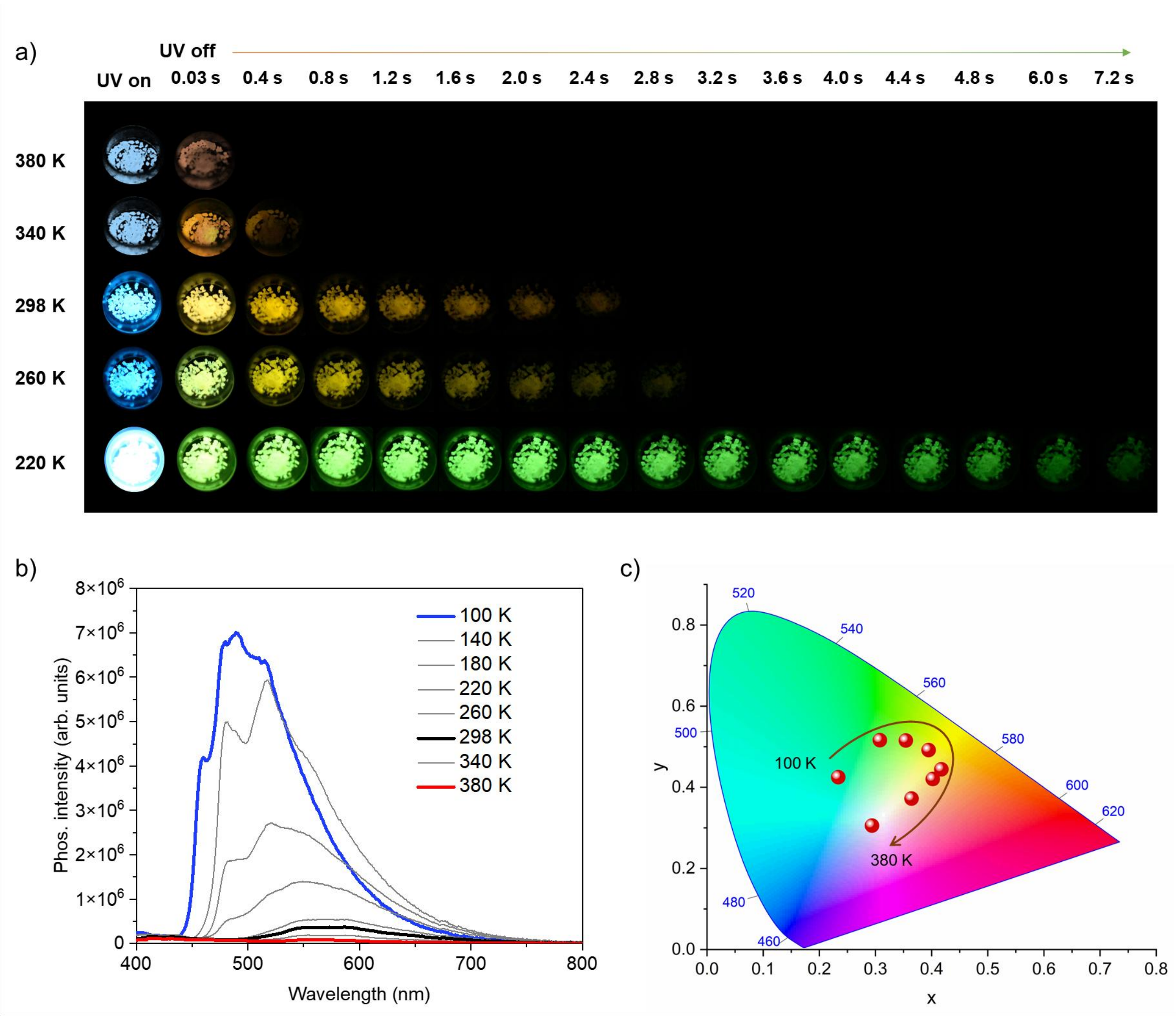

Figure S41. a) Afterglow photos of 1,2-PhAc crystals across 220 K–380 K (temperature is limited by the low-temperature cooling device, with a threshold of approximately 220 K). b) Delayed emission spectra across 100 K–380 K and c) CIE 1931 chromaticity diagram of 1,2-PhAc crystals at 100 K-380 K. The experiment was repeated independently 3 times with similar results.

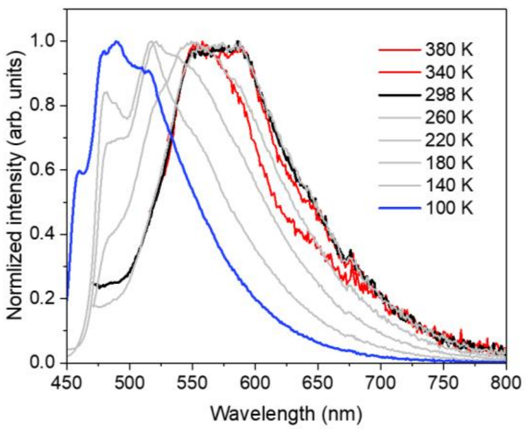

Figure S42. Normalized temperature-dependent delayed emission spectra of 1,2-PhAc crystal across 100-380 K shown as a 1D plot.

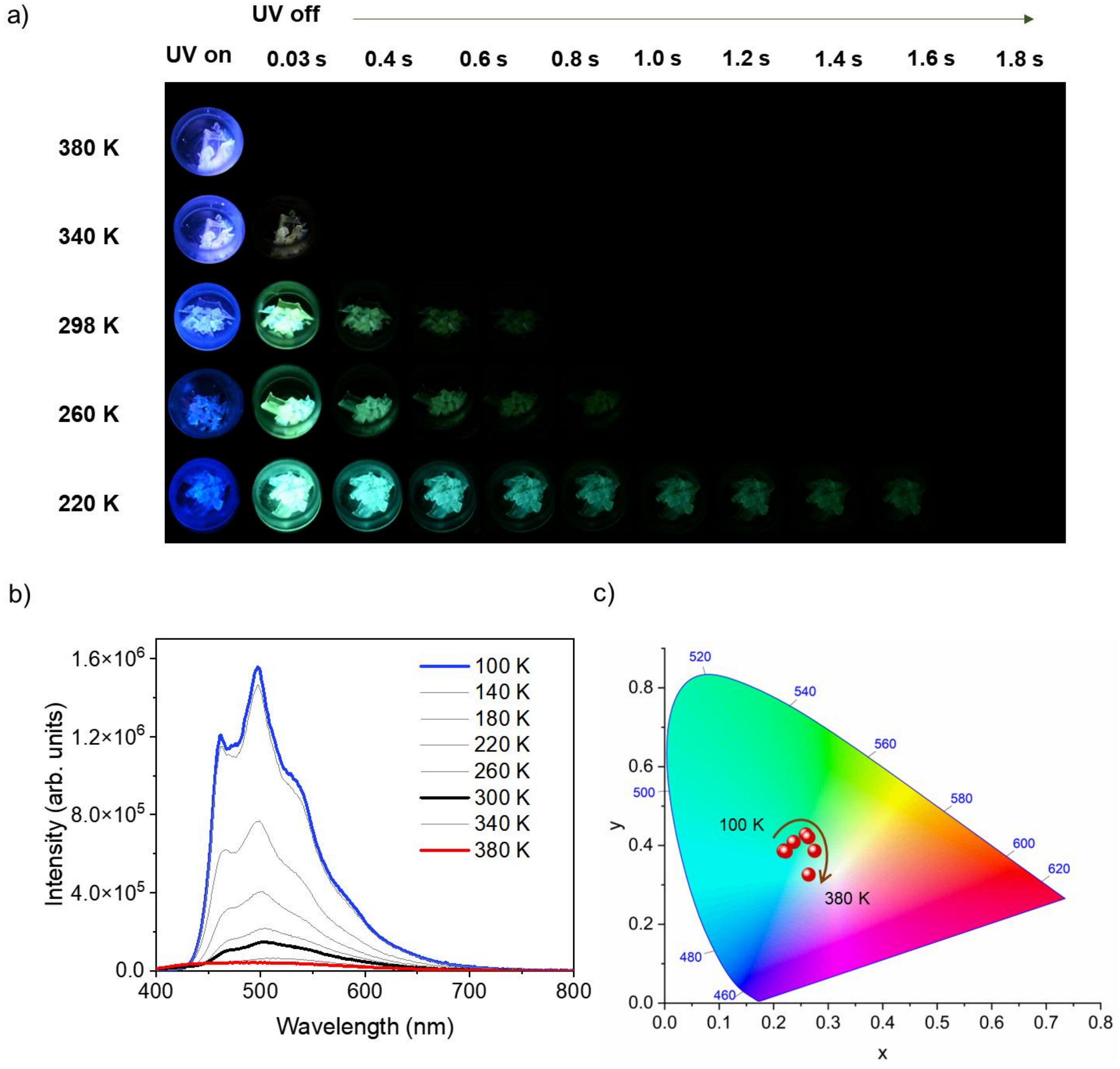

Figure S43. a) Afterglow photos of Ph-Ac crystals across 220 K–380 K. b) Delayed emission spectra and c) CIE 1931 chromaticity diagram of Ph-Ac crystals at 100 K-380 K. The experiment was repeated independently 3 times with similar results.

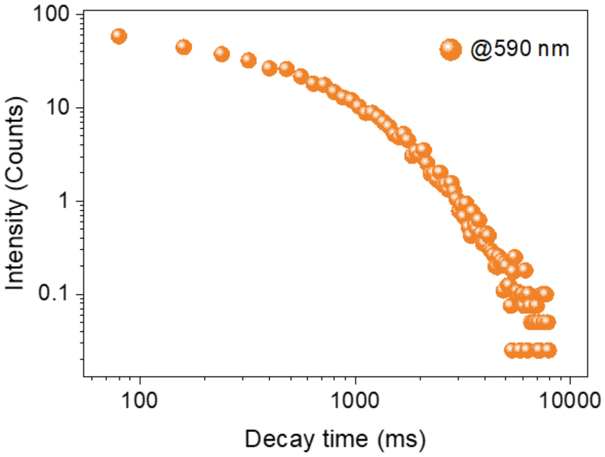

Figure S44. Time-resolved emission decay kinetics in 1,2-PhAc crystal at 590 nm from TRES, plotted on a log–log scale.

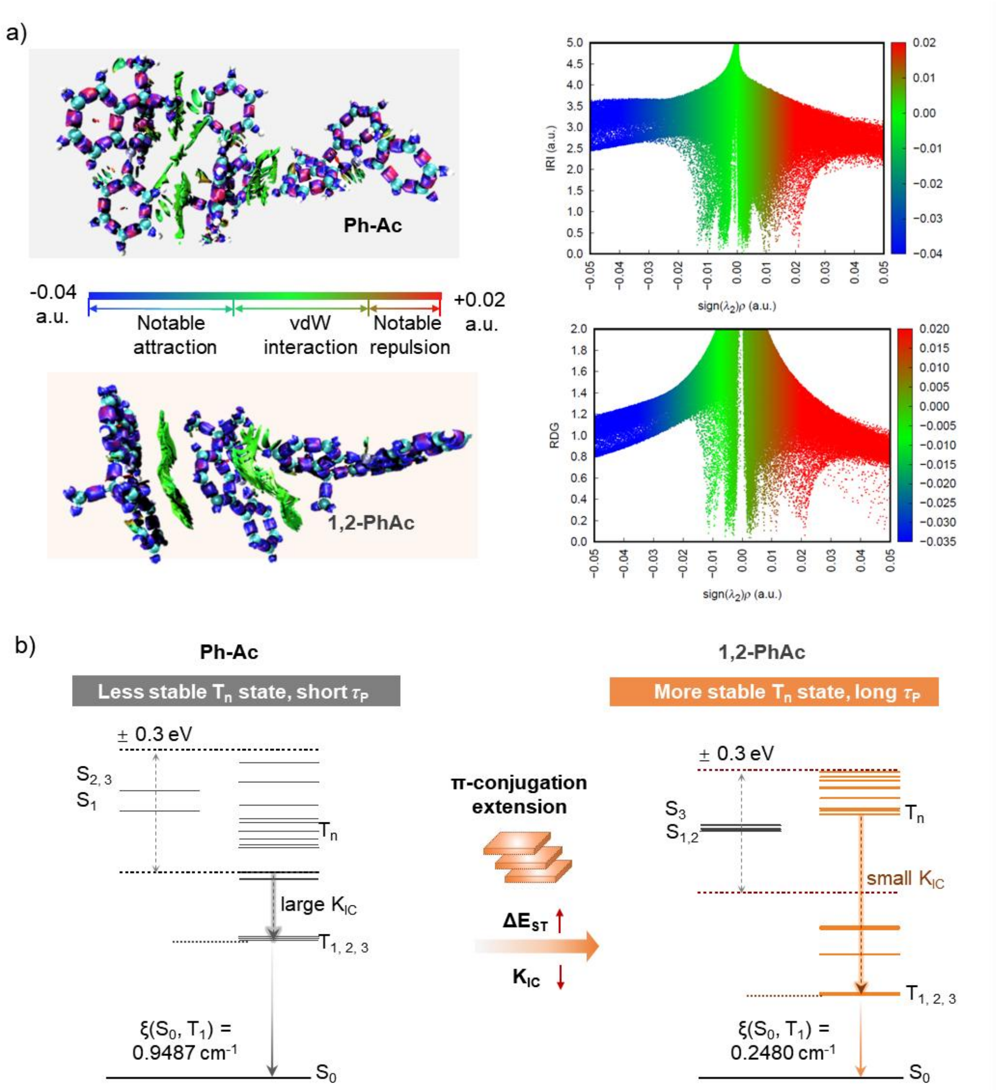

Figure S45. a) Gradient isosurfaces and the corresponding scatter diagrams of Ph-Ac and 1,2-PhAc trimer for IRI versus sign $(\lambda_2)\rho$[5]. b) Calculated SOC constants and energy gaps of Ph-Ac and 1,2-PhAc trimer. (The molecular geometries were extracted from the corresponding single crystals.

Table S15. The energy level of Ph-Ac and 1,2-PhAc trimer in the crystal state

Ph-Ac				1,2-PhAc			
	n-th	Energy level (eV)	ξ(S_1, T_n)		n-th	Energy level (eV)	ξ(S_1, T_n)
S_n	1	3.9250		S_n	1	3.8174	
T_n	1	3.2967		T_n	1	3.0192	
	2	3.3037			2	3.0278	
	3	3.3133			3	3.0292	
	4	3.5890			4	3.2154	
	5	3.5942			5	3.2170	
	6	3.5943			6	3.2384	
	7	3.6240			7	3.3407	
	8	3.6253	0.0084		8	3.3481	
	9	3.6297	0.6556		9	3.3510	
	10	3.7469	0.0081		10	3.8999	0.0168
	11	3.7591	0.0005		11	3.9178	0.1917
	12	3.7879	0.4088		12	3.9287	0.3130
	13	3.827	0.5107		13	3.9795	0.0176
	14	3.8712	0.0001		14	4.0250	0.1671
	15	3.890	0.0040		15	4.0308	0.0747
	16	3.9523	0.7366		16	4.0329	0.1684
	17	4.0677	0.0007		17	4.0628	0.1609
	18	4.1610	0.0032		18	4.0666	0.1942
	19	4.2593			19	4.0814	0.0891
	20				20	4.0829	0.1146
	21				21	4.1078	0.0642
	22				22	4.1529	

2.4 Photophysical property of 1,2-NpAc and 2,3-NpAc

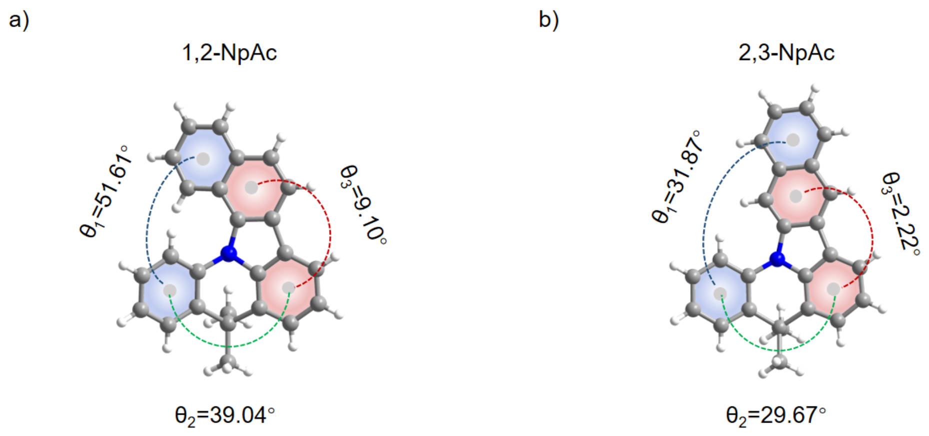

Figure S46. The molecular conformation of 1,2-NpAc and 2,3-NpAc (the geometry structure is from the crystal structure).

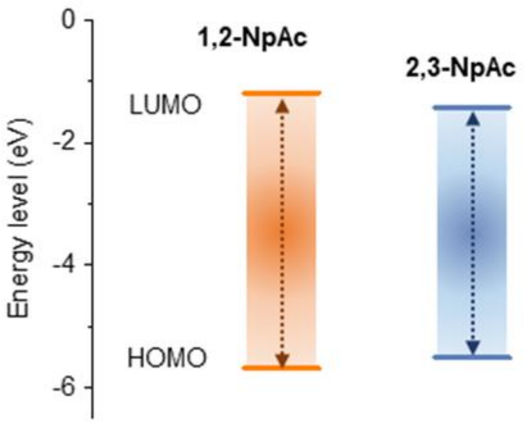

Figure S47. The HOMO and LUMO energy levels of 1,2-NpAc and 2,3-NpAc.

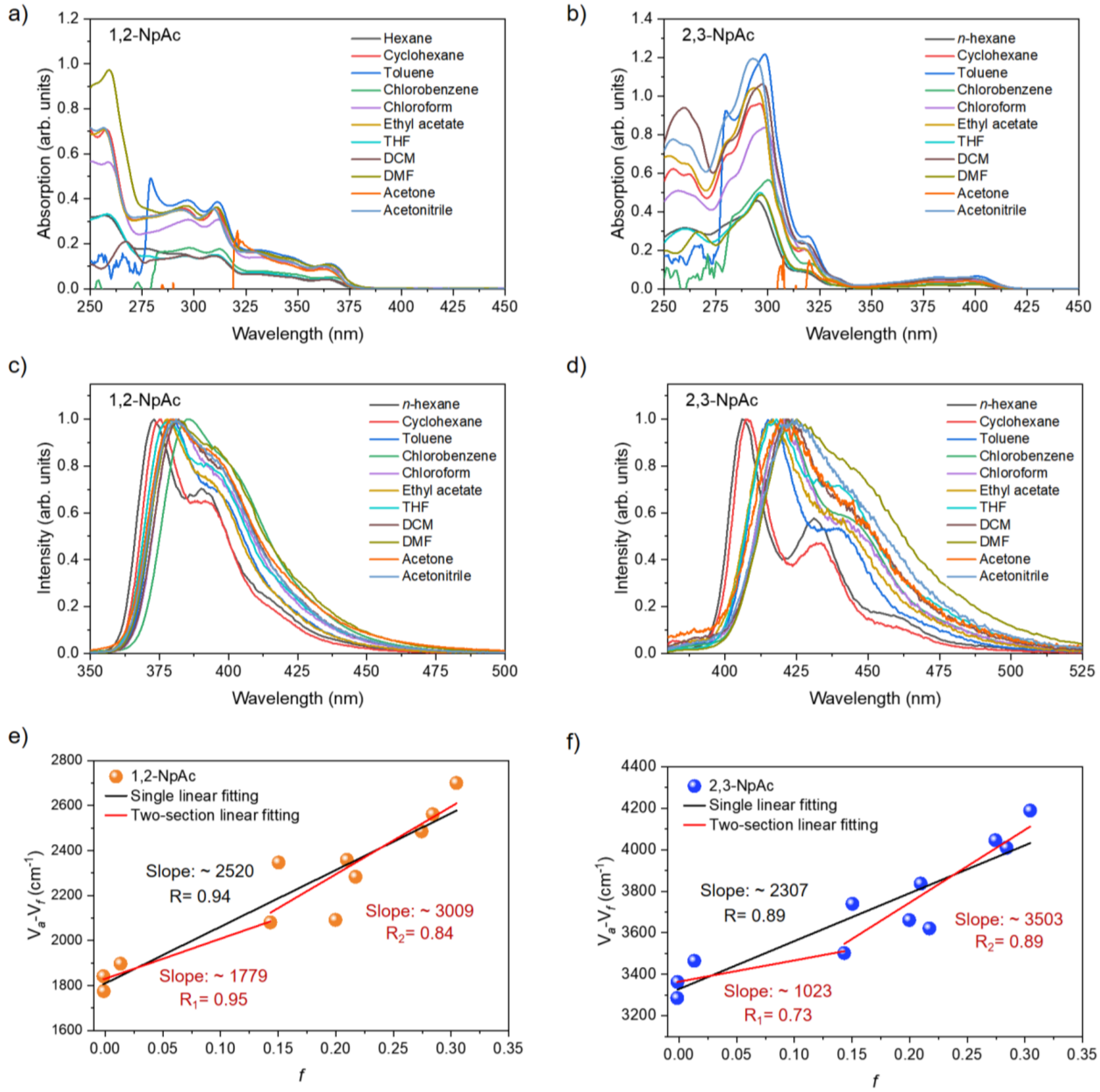

Figure S48. a-b) Absorption and c-d) PL spectra of 1,2-NpAc and 2,3-NpAc (10 μM) in different solution at room temperature and e-f) The solvatochromic Lippert–Mataga models of 1,2-NpAc and 2,3-NpAc (V_a-V_f = $(1/\lambda_{abs} – 1/\lambda_{PL}) * 10^7$).

Table S16. Solvatochromic effect investigation of 1,2-NpAc and 2,3-NpAc

Solvent	f (ε, n)	ν_a-ν_f (cm^{-1})	
		1,2-NpAc	2,3-NpAc
Cyclohexane	-0.002	1841.268	3285.129
n-hexane	-0.001	1775.013	3362.936
Toluene	0.013	1897.08	3464.909
Chlorobenzene	0.143	2080.804	3502.116
Chloroform	0.150	2346.890	3739.36
Ethyl acetate	0.199	2091.817	3661.116
THF	0.210	2359.292	3836.575
Dichloromethane	0.217	2283.611	3620.961
DMF	0.274	2485.553	4045.629
Acetone	0.284	2561.653	4009.516
Acetonitrile	0.304	2700.831	4188.199
a (Å)		6.03	5.92
$\Delta\mu = (\mu_e - \mu_g)^a$		8.3 D	8.8 D
$\Delta\mu = (\mu_e - \mu_g)^b$		7.1 / 9.0 D	5.8 / 10.8 D
μ_g		0.9 D	1.0 D
μ_e^a		9.2 D	9.8 D
μ_e^b		8.0 / 9.9 D	6.8 / 11.8 D

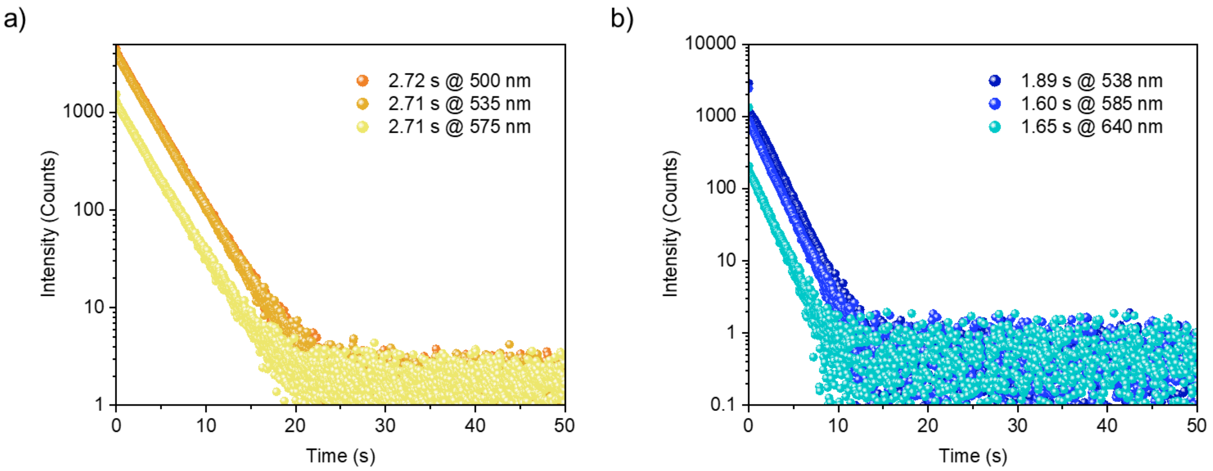

Figure S49. The delayed emission decays of a) 1,2-NpAc and b) 2,3-NpAc in THF solution (10 μM) at 77 K.

Table S17. Multiexponential fitting parameters of the delayed emission decay in THF at 77 K

Sample	τ_1 (s)	Rel.%	τ (s)	χ^2
1,2-NpAc@500 nm	2.72	100	2.72	0.999
1,2-NpAc@535 nm	2.72	100	2.72	0.999
1,2-NpAc@575 nm	2.71	100	2.71	0.999
2,3-NpAc@538 nm	1.89	100	1.89	0.999
2,3-NpAc@585 nm	1.60	100	1.60	0.990
2,3-NpAc@640 nm	1.65	100	1.65	0.999

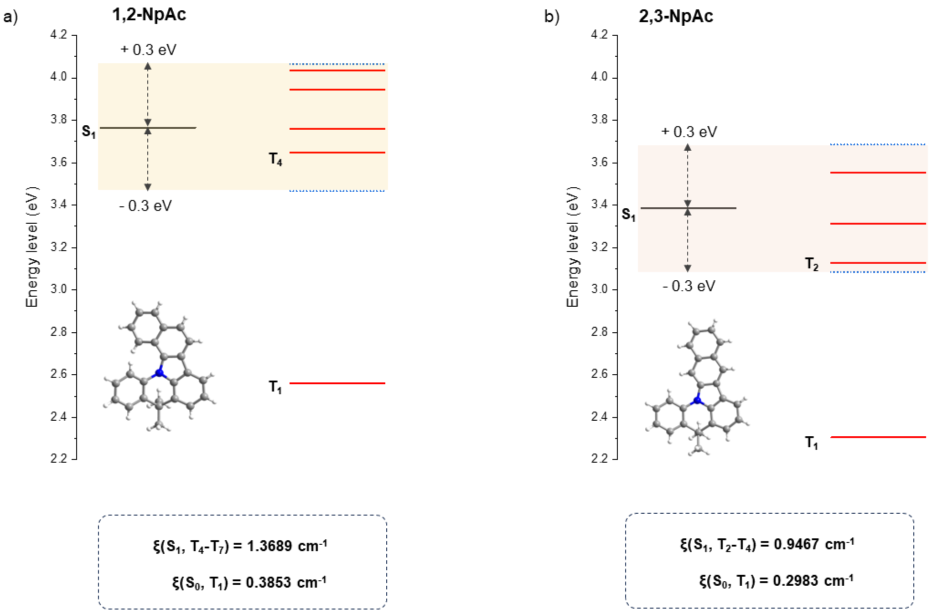

Figure S50. Calculated spin-orbit coupling (SOC) constants and energy gaps of a) 1,2-PhAc and b) 1,2-NpAc for the intersystem crossing process. (The molecular geometries were extracted from the corresponding single crystals)

Table S18. The energy level and SOC of 1,2-NpAc and 2,3-NpAc.

1,2-NpAc				2,3-NpAc.			
	n-th	Energy level (eV)	ξ (S_1, T_n)		n-th	Energy level (eV)	ξ (S_1, T_n)
S_n	1	3.7662		S_n	1	3.3851	
T_n	1	2.5590		T_n	1	2.3048	
	2	3.2649			2	3.1283	0.5568
	3	3.3621			3	3.3115	0.2384
	4	3.6472	0.3411		4	3.5545	0.1516
	5	3.7596	0.3352		5	3.7361	
	6	3.9464	0.4937				
	7	4.0341	0.1991				
	8	4.1829					

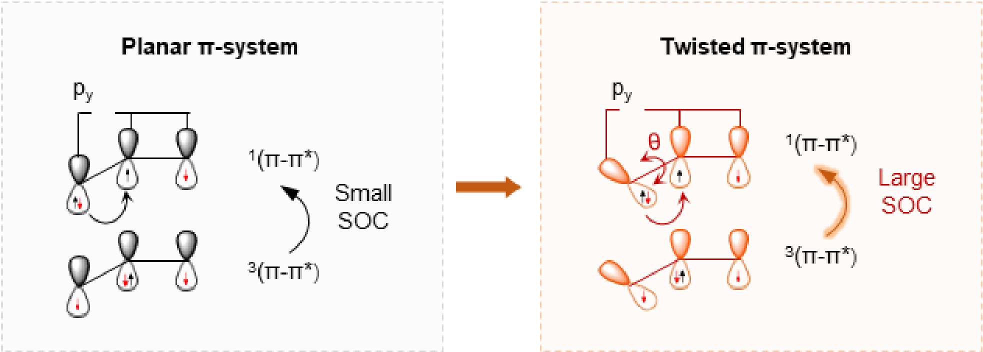

Figure S51. Schematic illustration of the effect of molecular conformation on SOC between excited singlet and triplet states.

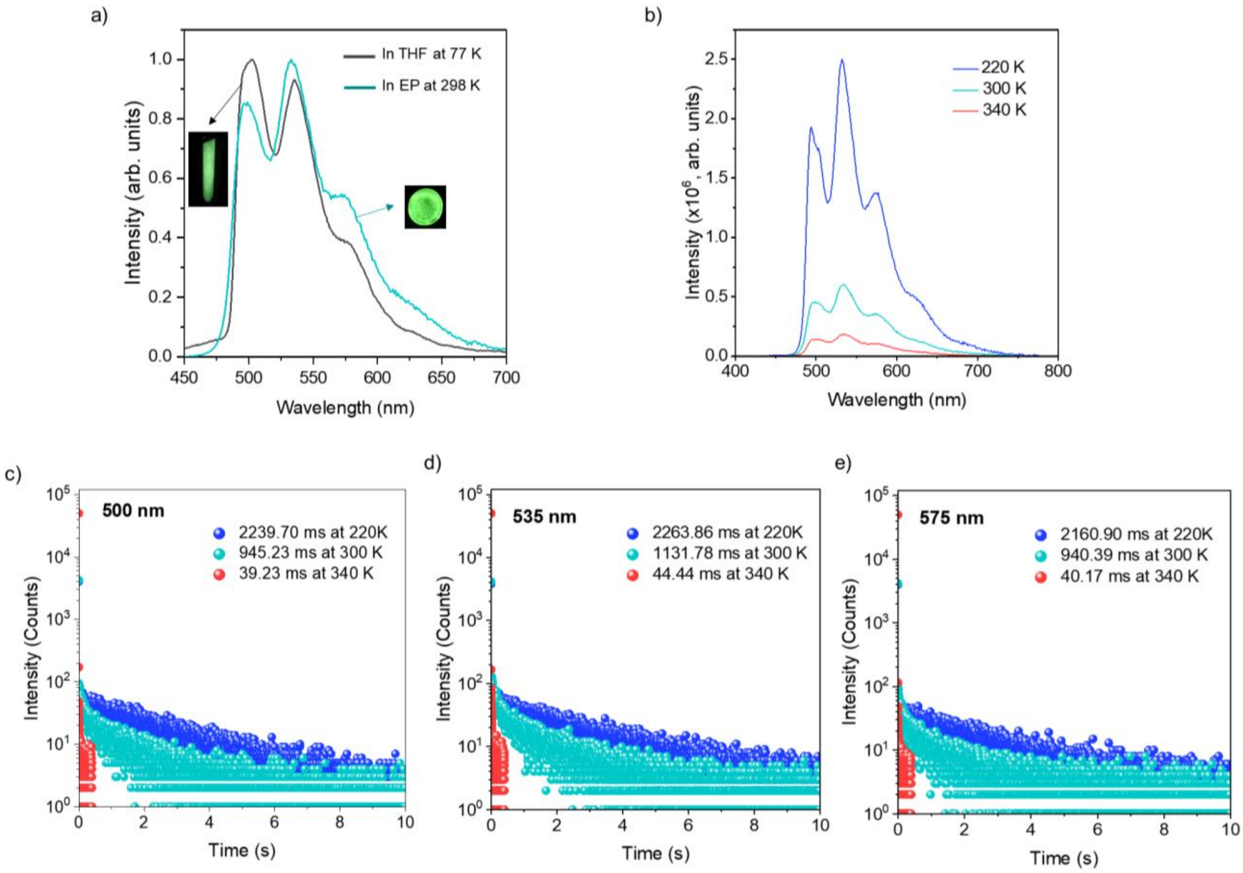

Figure S52. a) The delayed emission spectra (1 ms delay) of 1,2-NpAc in THF at 77 K and in epoxy resin (EP) at 298 K. b) Delayed emission spectra (1 ms delay) at different temperatures. The delayed emission decay profiles of 1,2-NpAc@epoxy resin monitored at c) 500 nm, d) 535 nm and e) 575 nm at different temperatures. (EP: epoxy resin)

Table S19. Multiexponential fitting parameters of the prompt emission decay in epoxy resin across

	τ_1 (ms)	Rel.%	τ_2 (ns)	Rel.%	τ (ms)	χ^2
1,2-NpAc@500 nm-340 K	3.22	29.18	54.07	70.82	39.23	1.017
1,2-NpAc@500 nm-300 K	945.23	100	-	-	945.23	0.995
1,2-NpAc@500 nm-220 K	2239.7	100	-	-	2239.70	1.078
1,2-NpAc@535 nm-340 K	3.15	23.72	57.28	76.28	44.44	1.149
1,2-NpAc@535 nm-300 K	158.56	20.42	1381.50	79.58	1131.78	0.939
1,2-NpAc@535 nm-220 K	287.19	1.47	2293.35	98.53	2263.86	1.080
1,2-NpAc@575 nm-340 K	3.44	28.84	55.06	71.16	40.17	1.037
1,2-NpAc@575 nm-300 K	125.35	21.42	1162.56	78.58	940.39	0.991
1,2-NpAc@575 nm-220 K	239.75	2.54	2210.97	997.46	2160.90	1.100

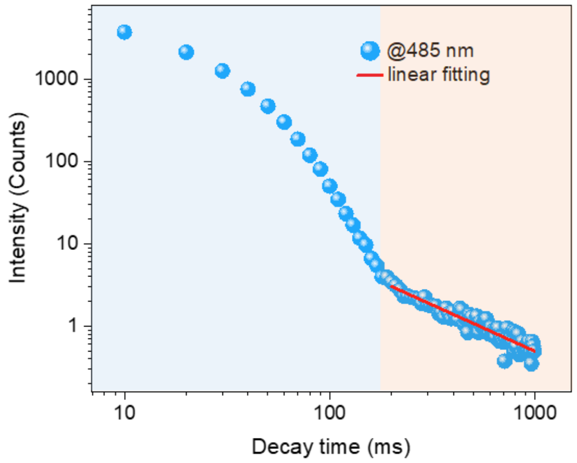

Figure S53. Time-resolved emission decay kinetics in 1,2-NpAc crystal at 485 nm from TRES, plotted on a log–log scale.

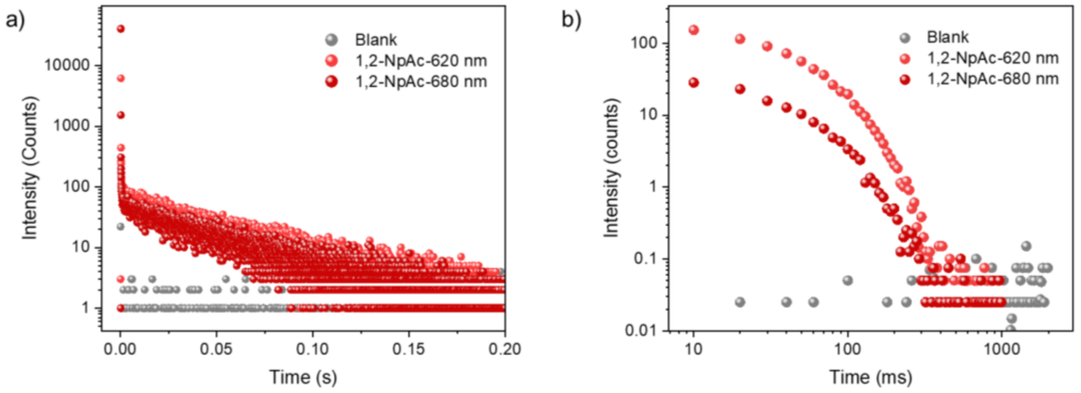

Figure S54. a) Delayed emission decay profiles of 1,2-NpAc crystals monitored at 620 and 680 nm, and the background (blank). b) Time-resolved emission decay kinetics of 1,2-NpAc crystals monitored at 620 and 680 nm, and the background (blank) from TRES, plotted on a log–log scale.

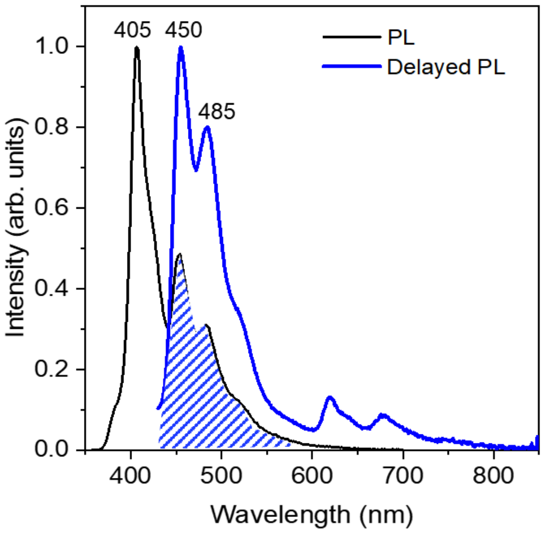

Figure S55. The prompt photoluminescence (PL) spectra and delayed emission spectra (1 ms delay) of 1,2-NpAc crystals.

Table S20. Multiexponential fitting parameters of the delayed emission decay in the crystal state across 220 K-380 K

	τ_1 (ms)	Rel.%	τ_2 (ms)	Rel.%	τ_3 (ms)	Rel.%	τ (ms)	χ^2
1,2-NpAc@450 nm-220 K	3.75	41.46	24.81	31.10	115.78	27.44	41.04	1.050
1,2-NpAc@450 nm-260 K	2.32	6.67	38.21	69.18	88.69	24.15	48.01	1.017
1,2-NpAc@450 nm-300 K	10.15	17.98	24.11	80.22	162.95	1.79	24.08	1.117
1,2-NpAc@450 nm-340 K	3.28	53.51	8.03	44.74	46.41	1.75	6.16	1.038
1,2-NpAc@450 nm-380 K	1.08	19.60	2.67	21.93	45.59	0.07	2.64	1.001
1,2-NpAc@485 nm-220 K	4.40	40.46	28.25	36.22	164.27	23.33	50.34	1.133
1,2-NpAc@485 nm-260 K	1.70	19.01	56.28	4.98	22.69	72.33	45.11	1.088
1,2-NpAc@485 nm-300 K	9.91	18.44	23.87	80.16	174.64	1.40	23.41	1.072
1,2-NpAc@485 nm-340 K	2.88	47.64	7.36	50.54	37.08	1.81	5.76	0.985
1,2-NpAc@485 nm-380 K	1.02	79.60	2.54	18.94	39.06	1.46	1.86	0.939
1,2-NpAc@620 nm-220 K	0.34	3.55	101.82	96.45	-	-	98.22	0.914
1,2-NpAc@620 nm-260 K	0.25	2.26	90.33	97.73	-	-	88.29	0.910
1,2-NpAc@620 nm-300 K	1.56	1.57	40.60	98.43	-	-	39.98	1.082
1,2-NpAc@620 nm-340 K	0.84	16.96	9.23	83.04	-	-	7.81	1.019
1,2-NpAc@620 nm-380 K	0.45	42.26	2.16	57.74	-	-	1.44	0.928
1,2-NpAc@680 nm-220 K	0.42	2.87	99.05	97.13	-	-	96.22	0.909
1,2-NpAc@680 nm-260 K	0.30	1.42	88.22	98.58	-	-	86.97	1.131
1,2-NpAc@680 nm-300 K	0.22	2.92	39.04	97.08	-	-	37.91	1.008
1,2-NpAc@680 nm-340 K	0.02	66.86	8.26	33.14			2.75	0.947
1,2-NpAc@680 nm-380 K	0.02	21.53	1.54	78.47	-	-	1.21	1.011

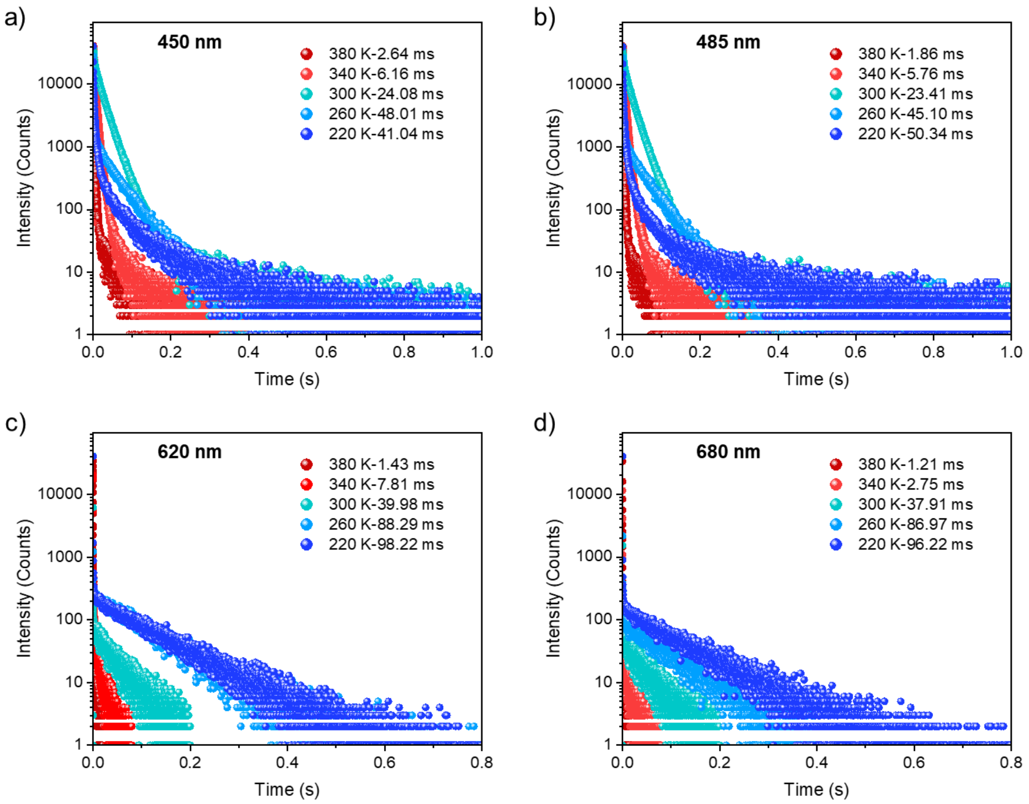

Figure S56. Temperature-dependent delayed emission decay profiles of 1,2-NpAc crystals monitored at a) 450 nm, 485 nm, 620 nm, and 680 nm over the temperature range of 220 K–380 K.

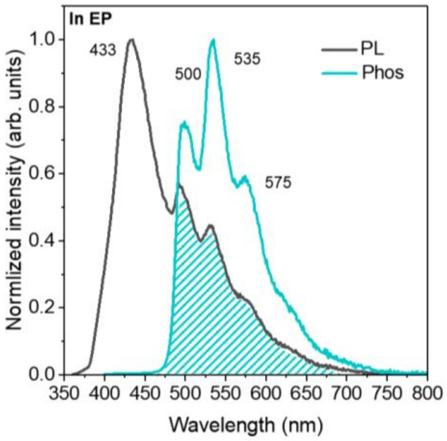

Figure S57. The prompt photoluminescence (PL) spectra and phosphorescence (Phos) spectra of 1,2-NpAc@epoxy resin (EP: epoxy resin).

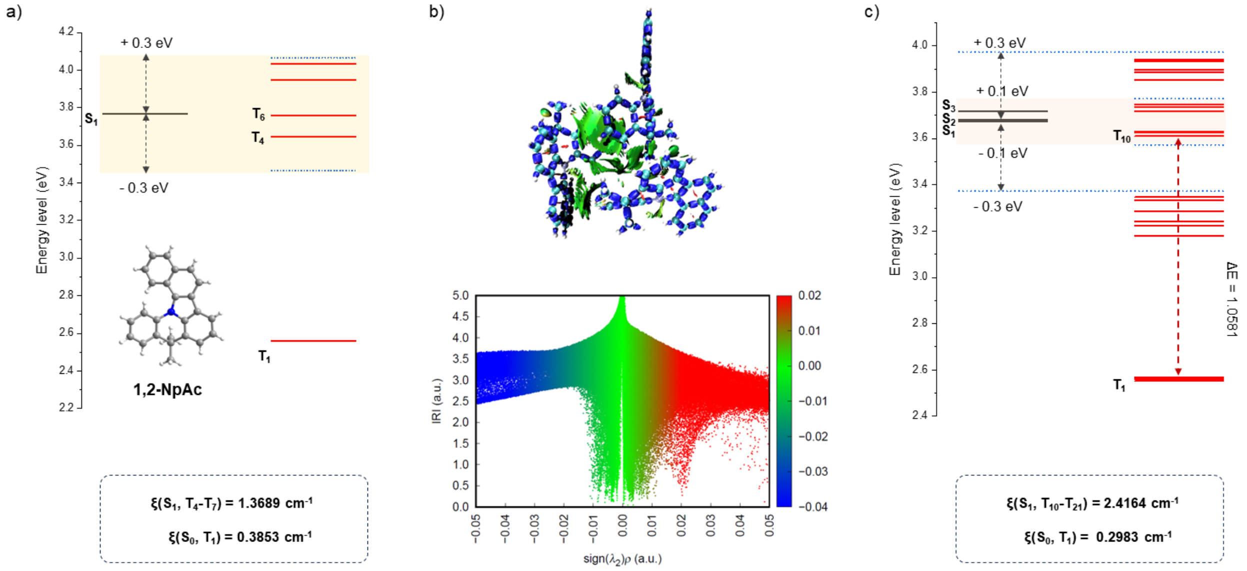

Figure S58. a) Calculated SOC constants and energy level of 1,2-NpAc at isolated state. b) Gradient isosurfaces and the corresponding scatter diagrams of 1,2-NpAc trimer for IRI versus sign $(\lambda_2)\rho$. c) Calculated SOC constants and energy gaps of 1,2-NpAc trimer. (The molecular geometries were extracted from the corresponding single crystals).

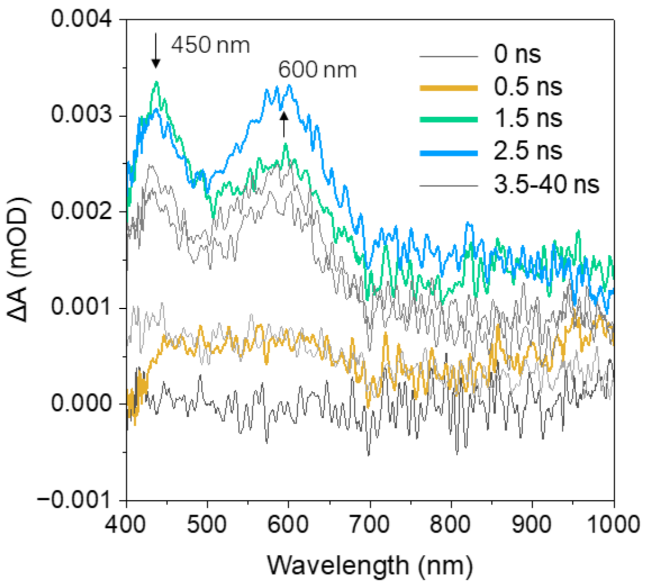

Figure S59. Nanosecond-TA spectroscopy of 1,2-NpAc crystal.

Table S21. Crystal data of 1,2-NpAc and 2,3-NpAc

Name	1,2-NpAc	2,3-NpAc
Formula	$C_{25}H_{19}N$	$C_{25}H_{19}N$
Crystal system	Monoclinic	Monoclinic
Space Group	C2/c	P 21/c
Cell Length (Å)	a = 27.268 (9)<br>b = 11.392 (4)<br>c = 22.881 (8)	a = 11.208 (3)<br>b = 6.175 (2)<br>c = 25.365 (9)
Cell Angles (°)	α = 90<br>β = 96.205 (10)<br>γ = 90	α = 90<br>β = 98.49 (3)<br>γ = 90
Cell Volume ($Å^3$)	7066.1 (4)	1736.4 (10)
z	8	4
Density (g/cm^3)	1.254	1.275
F (000)	2816	704
h_{max}, k_{max}, l_{max}	$-32 \leq h \leq 32$,<br>$-13 \leq k \leq 13$,<br>$-27 \leq l \leq 27$	$-13 \leq h \leq 14$,<br>$-3 \leq k \leq 7$,<br>$-31 \leq l \leq 30$
CCDC	2491721	2491722

Table S22. The energy level of 1,2-NpAc trimer in the crystal state

1,2-NpAc			
	n-th	Energy level (eV)	ξ(S_1, T_n)
S_n	1	3.6728	
T_n	1	2.5518	
	2	2.5607	
	3	2.5683	
	4	3.1782	
	5	3.2236	
	6	3.2411	
	7	3.2860	
	8	3.3344	
	9	3.3464	
	10	3.6099	0.2087
	11	3.6242	0.2204
	12	3.6309	0.1753
	13	3.7190	0.1984
	14	3.7350	0.3414
	15	3.7473	0.0901
	16	3.8518	0.0767
	17	3.8850	0.1588
	18	3.8985	0.0837
	19	3.9342	0.3110
	20	3.9413	0.1376
	21	3.9418	0.4143
	22	4.0028	

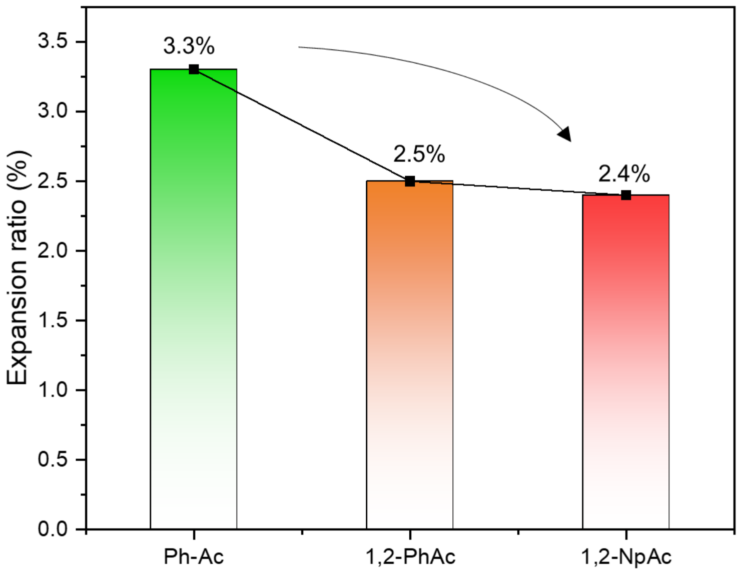

Figure S60. Comparison of the cell volume expansion ratios for Ph-Ac, 1,2-PhAc, and 1,2-NpAc crystals.

Table S23. The cell volume expansion ratios for Ph-Ac, 1,2-PhAc, and 1,2-NpAc crystals

Sample	Cell volume (Å^3)		Expansion ratios[a] (%)
	V_1	V_2	
Ph-Ac	1590.34 (220 K)	1642.44 (360 K)	3.3
1,2-PhAc	1459.15 (220 K)	1496.03 (380 K)	2.5
1,2-NpAc	6980.74 (220 K)	7148.00 (380 K)	2.4

[a] Expansion ratios = $(V_2-V_1)/V_1 * 100\%$

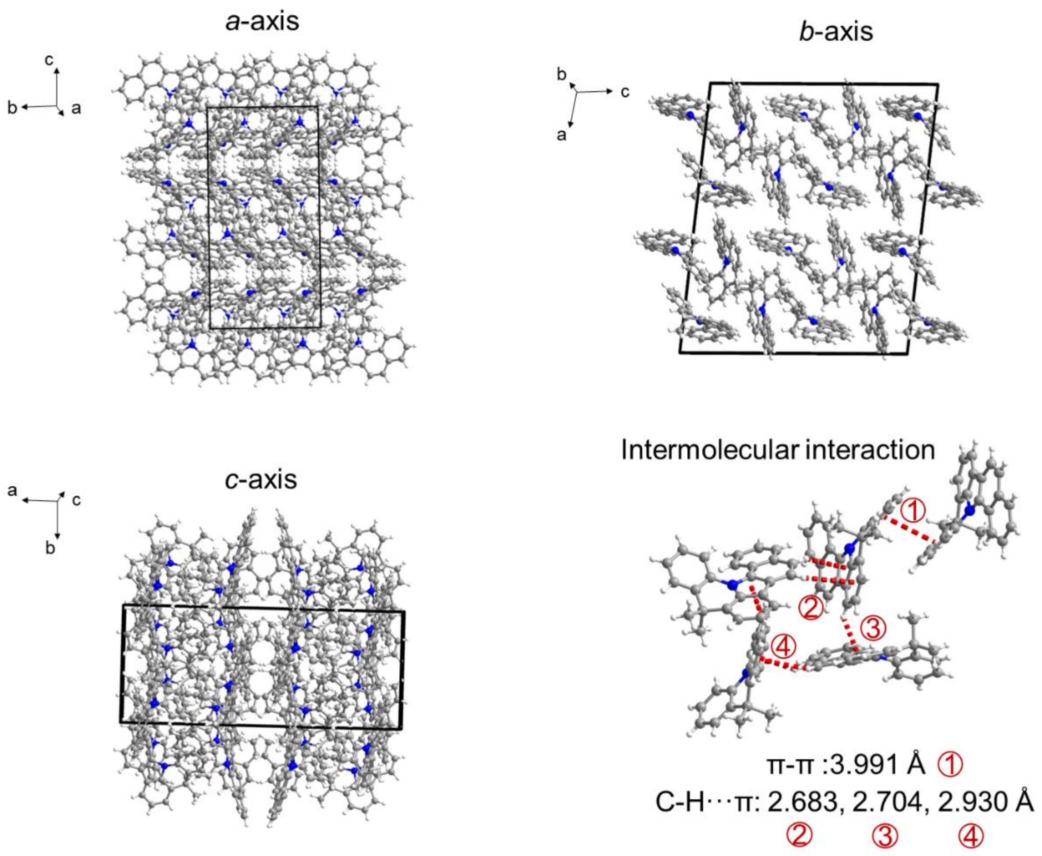

Figure S61. Packing modes along *a*-, *b*- and *c*-axis and intermolecular interactions of 1,2-NpAc crystals.

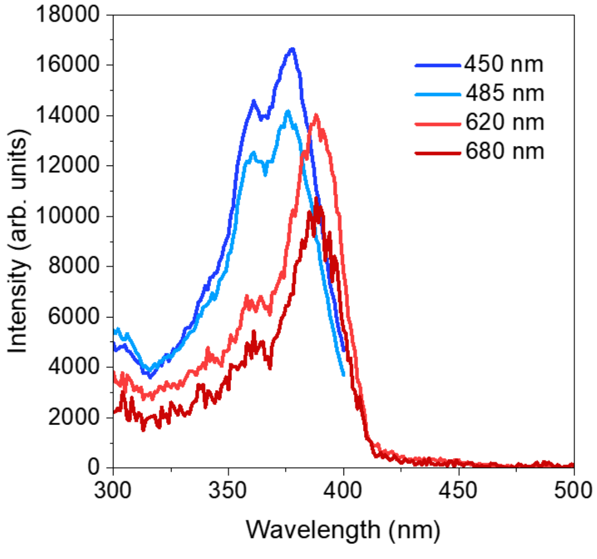

Figure S62. Excitation spectra of 1,2-NpAc crystals recorded at emission wavelengths of 450, 485, 620, and 680 nm.

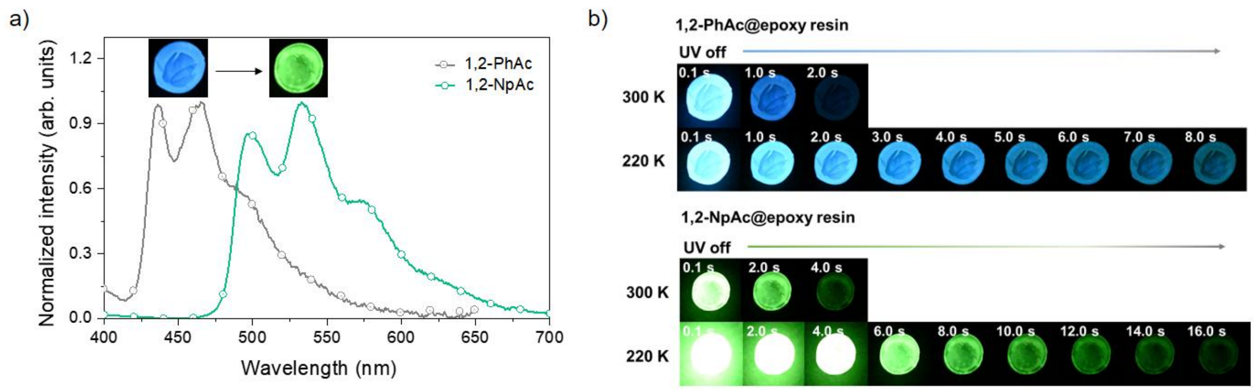

Figure S63. a) The phosphorescence spectra of 1,2-PhAc@epoxy resin and 1,2-NpAc@epoxy resin and corresponding their afterglow photos. The experiment was repeated independently 3 times with similar results.

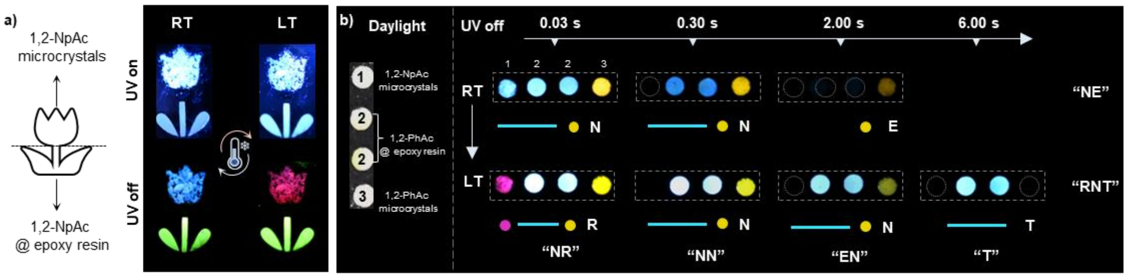

Figure S64. a) Afterglow patterns with temperature-responsive characterization (petal: 1,2-NpAc microcrystals, leaf: 1,2-NpAc@epoxy resin). b) Reading and decryption of recyclable temperature/time-related bimodal encrypted messaging system using 1,2-NpAc microcrystals, 1,2-PhAc@epoxy resin and 1,2-PhAc microcrystals. The experiment was repeated independently 3 times with similar results.

3. Supplementary References

[1] Mataga N., Kaifu Y., Koizumi M. Solvent effects upon fluorescence spectra and the dipolemoments of excited molecules. *Bull. Chem. Soc. Jpn.* **29**, 465–470 (1956).

[2] Lippert E. Dipolmoment und elektronenstruktur von angeregten molekülen. *Z. Naturforsch.* **10a**, 541–545 (1955).

[3] Li X. -Y. An Overview of continuum models for nonequilibrium solvation: popular theories and new challenge. *Int. J. Quantum Chem.* **115**, 700–721 (2015).

[4] Cinar R., Nordmann J., Dirksen E., Müller T. J. J. Domino synthesis of protochromic "ON–OFF–ON" luminescent 2-styryl quinolines. *Org. Biomol. Chem.* **11**, 2597–2604 (2013).

[5] Lu, T., Chen, Q. Interaction region indicator: a simple real space function clearly revealing both chemical bonds and weak interactions. *Chem. Methods* **1**, 231–239 (2021).
