# Supplementary material for: High energy triplet-state manipulation via temperature-responsive twisted hetero-annulation systems
Source: Nat Commun. 2026 May 25;17:6790. doi: 10.1038/s41467-026-73715-8 (PMC13385895; doi:10.1038/s41467-026-73715-8)
Supplement: Supplementary file 2 — Description of Additional Supplementary Files [file 41467_2026_73715_MOESM2_ESM.pdf]

Description of Additional Supplementary Files

File Name: Supplementary Data 1
Description: The atomic coordinates of the optimized computational models
