# Supplementary material for: High energy triplet-state manipulation via temperature-responsive twisted hetero-annulation systems
Source: Nat Commun. 2026 May 25;17:6790. doi: 10.1038/s41467-026-73715-8 (PMC13385895; doi:10.1038/s41467-026-73715-8)
Supplement: Supplementary file 4 — Reporting Summary [file 41467_2026_73715_MOESM4_ESM.pdf]

nature portfolio

Corresponding author(s): Qianqian Li

Last updated by author(s): May 13, 2026

Reporting Summary

Nature Portfolio wishes to improve the reproducibility of the work that we publish. This form provides structure for consistency and transparency in reporting. For further information on Nature Portfolio policies, see our Editorial Policies and the Editorial Policy Checklist.

Statistics

For all statistical analyses, confirm that the following items are present in the figure legend, table legend, main text, or Methods section.

n/a	Confirmed	
☐	☒	The exact sample size (n) for each experimental group/condition, given as a discrete number and unit of measurement
☐	☒	A statement on whether measurements were taken from distinct samples or whether the same sample was measured repeatedly
☒	☐	The statistical test(s) used AND whether they are one- or two-sided<br>*Only common tests should be described solely by name; describe more complex techniques in the Methods section.*
☐	☒	A description of all covariates tested
☒	☐	A description of any assumptions or corrections, such as tests of normality and adjustment for multiple comparisons
☐	☒	A full description of the statistical parameters including central tendency (e.g. means) or other basic estimates (e.g. regression coefficient) AND variation (e.g. standard deviation) or associated estimates of uncertainty (e.g. confidence intervals)
☒	☐	For null hypothesis testing, the test statistic (e.g. F, t, r) with confidence intervals, effect sizes, degrees of freedom and P value noted<br>*Give P values as exact values whenever suitable.*
☒	☐	For Bayesian analysis, information on the choice of priors and Markov chain Monte Carlo settings
☒	☐	For hierarchical and complex designs, identification of the appropriate level for tests and full reporting of outcomes
☒	☐	Estimates of effect sizes (e.g. Cohen's d, Pearson's r), indicating how they were calculated

Our web collection on statistics for biologists contains articles on many of the points above.

Software and code

Policy information about availability of computer code

Data collection	TD-DFT/DFT calculations were performed on Gaussian 09 program (Revision D01) and Gaussian 16, Revision C.01.The spin-orbital coupling (SOC) values were performed through the same functional and basis using PySOC package with python 2.7. The non-covalent interaction (NCI) analysis, interaction region indicator (IRI) and electrostatic potential analysis were performed through Multiwfn 3.8 and VMD.All the structures were resolved and analyzed with the assistance of olex2 software.
Data analysis	TD-DFT/DFT calculations were performed on Gaussian 09 program (Revision D01) and Gaussian 16, Revision C.01.The spin-orbital coupling (SOC) values were performed through the same functional and basis using PySOC package with python 2.7. The non-covalent interaction (NCI) analysis, interaction region indicator (IRI) and electrostatic potential analysis were performed through Multiwfn 3.8 and VMD.All the structures were resolved and analyzed with the assistance of olex2 software.

For manuscripts utilizing custom algorithms or software that are central to the research but not yet described in published literature, software must be made available to editors and reviewers. We strongly encourage code deposition in a community repository (e.g. GitHub). See the Nature Portfolio guidelines for submitting code & software for further information.

Data

Policy information about availability of data

All manuscripts must include a data availability statement. This statement should provide the following information, where applicable:

- Accession codes, unique identifiers, or web links for publicly available datasets
- A description of any restrictions on data availability
- For clinical datasets or third party data, please ensure that the statement adheres to our policy

All data supporting the findings of this study are available in the Manuscript, Supplementary Information and Source Data.

Research involving human participants, their data, or biological material

Policy information about studies with human participants or human data. See also policy information about sex, gender (identity/presentation), and sexual orientation and race, ethnicity and racism.

Reporting on sex and gender	No reporting on sex and gender.
Reporting on race, ethnicity, or other socially relevant groupings	No reporting on race, ethnicity, and other socially relevant groupings.
Population characteristics	No reporting on population characteristics.
Recruitment	No reporting on recruitment.
Ethics oversight	No reporting on the ethics oversight.

Note that full information on the approval of the study protocol must also be provided in the manuscript.

Field-specific reporting

Please select the one below that is the best fit for your research. If you are not sure, read the appropriate sections before making your selection.

☐ Life sciences ☐ Behavioural & social sciences ☒ Ecological, evolutionary & environmental sciences

For a reference copy of the document with all sections, see nature.com/documents/nr-reporting-summary-flat.pdf

Ecological, evolutionary & environmental sciences study design

All studies must disclose on these points even when the disclosure is negative.

Study description	This study focused on the synthesis and characterization of a series of organic luminogens. The structural variation was introduced through different substituents and fusion sites, which served as the main comparative factors in a structure–property relationship study. Each compound represents an independent experimental unit, and all photophysical measurements were performed on multiple independently prepared samples. For each structure, at least three independent measurements (n ≥ 3) were conducted to ensure reproducibility. The study follows a comparative experimental design rather than a factorial or hierarchical design, aiming to systematically correlate molecular structure with optical properties.
Research sample	This study does not involve biological organisms, human subjects, or existing datasets. The research samples are pure organic single crystals prepared in the laboratory. The starting materials were commercially purchase. The crystals were grown via a solution-based growth method under controlled laboratory conditions. No biological taxa, sex, age, or organism-related manipulations are applicable in this study.
Sampling strategy	Samples were selected from optically uniform single crystals grown in solution. No statistical power analysis was performed, as is standard in materials science studies. Sample size was determined based on experimental reproducibility, and measurements were repeated on multiple independent crystals (n ≥ 3) to ensure consistency of results.
Data collection	1H and 13C NMR spectra were characterized on a Bruker Avance III HD 400 MHz or Bruker AVANCE NEO 400 MHz. Mass spectra were recorded on a Shimadzu GCMS-QP2020 mass spectrophotometer. Elemental analyses were conducted on a UNICUBE. Photoluminescence and phosphorescence spectra in aggregated and solution states were determined on a FLS980 spectrometer. UV-vis absorption spectra were measured on Shimadzu UF5700 spectrometer. Lifetimes and quantum yields were measured with FLS980 spectrometer. The single-crystal X-ray diffraction data were collected on an XtaLAB Synergy Custom and Bruke D8. The transient absorption was measured using a Dalian Chuangrui instrument (TA100-DZ) and Ultrafast Systems (HELIOS). All the structures were resolved and analyzed with the assistance of olex2 software. High performance liquid chromatography (HPLC) was conducted on LaboACE LC-500. The photos were taken by Nikon Z9
Timing and spatial scale	Data collection for the spectroscopic measurements was conducted from January 2024 to March 2026. All measurements were acquired from a consistent spatial scale corresponding to the illuminated area of the sample within the spectrometer setup.
Data exclusions	No data were excluded from the analyses.

Reproducibility	Each experiment was independently repeated at least three times using different crystals from independent growth batches ($n \geq 3$). Consistent results were obtained across all measurements, confirming reproducibility.
Randomization	No group allocation was involved, as each compound was measured independently and comparisons were made across different compounds rather than between experimental groups.
Blinding	No blinding was performed during data acquisition or analysis, because all measurements were obtained using the relevant instrumentation, and the study design did not involve subjective assessments or comparisons between experimental groups.

Did the study involve field work? ☐ Yes ☒ No

Reporting for specific materials, systems and methods

We require information from authors about some types of materials, experimental systems and methods used in many studies. Here, indicate whether each material, system or method listed is relevant to your study. If you are not sure if a list item applies to your research, read the appropriate section before selecting a response.

Materials & experimental systems

n/a	Involved in the study
☒	☐ Antibodies
☒	☐ Eukaryotic cell lines
☒	☐ Palaeontology and archaeology
☒	☐ Animals and other organisms
☒	☐ Clinical data
☒	☐ Dual use research of concern
☒	☐ Plants

Methods

n/a	Involved in the study
☒	☐ ChIP-seq
☒	☐ Flow cytometry
☒	☐ MRI-based neuroimaging

Plants

Seed stocks	No reporting on the seed stocks.
Novel plant genotypes	No reporting on the novel plant genotypes.
Authentication	No reporting on the authentication.
