# Supplementary material for: High energy triplet-state manipulation via temperature-responsive twisted hetero-annulation systems
Source: Nat Commun. 2026 May 25;17:6790. doi: 10.1038/s41467-026-73715-8 (PMC13385895; doi:10.1038/s41467-026-73715-8)
Supplement: Supplementary file 5 — Transparent Peer Review File [file 41467_2026_73715_MOESM5_ESM.pdf]

nature portfolio

Peer Review File

High Energy Triplet-State Manipulation via Temperature-Responsive Twisted Hetero-Annulation Systems

Corresponding Author: Professor Qianqian Li

This file contains all reviewer reports in order by version, followed by all author rebuttals in order by version.

Version 0:

Reviewer comments:

Reviewer #1

(Remarks to the Author)
This manuscript entitled Modulating High-Energy Triplet State Transition Pathways by Twisted Hetero-annulation Strategy with Temperature as "Control Knobs" presents very interesting observations in organic afterglow systems with diverse photophysical behaviors. A series of organic compounds have been studied, some of which exhibit unusual afterglow property. The observation of TADF-type afterglow with large deltaEST is rare. Most of the properties are well interpreted either by experiments or by theoretical calculations. There are some comments attached in the following. This manuscript can be accepted for publication after revisions.
Comment 1. Regarding the title, twisted hetero-annulation seems not to be a strategy, so the word strategy may be removed. with temperature as "control knobs" seems to be difficult to follow. Does it mean controlled by temperature? A clear title may be better for this manuscript and for the readers.
Comment 2. In page 3, the sentence "3 pi-pi transition in Tn states, which can slow down the decay rates for the spin-forbidden transitions ...favoring the stabilization of Tn states" make sense. On the other hand, the Tn-T1 internal conversion would quench or consume Tn states rapidly. This should be considered in the discussion. The emergence of TADF-type afterglow with such large deltaEST needs further supports.
Comment 3. For the terms stabilize and stabilization, these would also refer to the lowering of energy levels of excited states. From the context, the authors use these terms to describe the protection of excited states or the suppression of nonradiative decay. These terms may be revised to avoid misunderstanding.
Comment 4. Tn emission is very rare. The assignment of Tn emission needs further support or ruling out other possible mechanism.

Reviewer #2

(Remarks to the Author)
Review work: “Modulating High-Energy Triplet State Transition Pathways by Twisted Hetero-annulation Strategy with Temperature as “Control Knobs”

The authors report a series of organic compounds designed to investigate how molecular rigidification, achieved through annulation, and π-system extension influence luminescent properties via modulation of singlet and triplet excited states. The photophysical behavior is first examined in solution (THF) and subsequently in the solid state, with particular emphasis on crystalline samples and the temperature dependence of their emission properties. Building on the insights gained from the initial molecule set on delayed luminescence, the study further explores how extending the π-conjugated framework impacts excited-state dynamics and emission characteristics.
The control and manipulation of excited states—particularly triplet states—represent an important and timely topic with clear relevance for the development of advanced luminescent materials and sensing applications. While the overall approach and objectives of the study are of significant interest, several aspects of the analysis and interpretation require further clarification and deeper discussion before the work can be considered for publication. I believe this paper will need re-review.

Major Issues:
General:

The authors attribute the observed temperature-dependent emission behavior primarily to hot-exciton–mediated processes, including hISC and hRISC, supported by DFT/TDDFT calculations and experimental observations. While this interpretation is plausible, the experimental results suggest that such behavior may not be an intrinsic molecular property alone, but rather strongly dependent on the solid-state environment. The authors should clarify the assignment of each of the processes they observe to unambiguously discuss their observation. Currently there is a lack of clarity on which each process is assigned to which observation.

In particular, in dilute solution or especially in resin matrices (Figure S53)—where intermolecular interactions and excitonic coupling are minimized—the compounds seem to exhibit relatively conventional photophysical behavior, without clear signatures of hot-exciton–driven emission. Even if the calculation results seem to indicate involvement of Tn state in ISC the energy of their predicted T1 can still be linked with emission around 450nm.
While afterglow can be observed in resin no temperature induced color-changed can be seen and I wonder if TADF type emission is observed for compound 1,2-NpAc.
In contrast, the most striking phenomena, such as temperature-dependent color switching and pronounced phosphorescence, only emerge in the crystalline state.

This raises the question of whether the observed effects originate primarily from intrinsic hot-exciton dynamics of individual molecules, or whether they arise from collective phenomena such as crystal packing, exciton delocalization, or rigidification-induced suppression of nonradiative decay. In this context, the role of the crystal lattice and intermolecular interactions may be as critical as the electronic structure of the isolated chromophores themselves.

The authors must discuss such possibilities in order to allow for a complete interpretation of their observed properties.

Lifetimes and decays:

The presentation of time-resolved data needs greater consistency. In the current manuscript, decay curves are shown using a mixture of logarithmic–linear and log–log representations (ex: Figure S40, Figure 3h), sometimes with the ordinate described as intensity in arbitrary units and other times in photon counts. This variability complicates direct comparison between datasets. A consistent representation—preferably using logarithmic photon counts versus linear time, which is standard for phosphorescence and TADF analysis—is necessary to improve clarity and facilitate comparison of decay dynamics across samples and temperatures.
Moreover, in some cases the reported photon counts appear to be close to the noise level (below 100). which complicates the interpretation of the decay behaviour. For clarity and reproducibility, all decay traces including the corresponding fitting curves and χ^2 values should be presented in the SI

Page 6 line 115-117: The authors report "Accordingly, the photoluminescence quantum yields (PLQYs) largely increased from Ph-X series to 1,2-PhX ones, for instance, the PLQY of Ph-POZ was 9.64%, while that of 1,2-PhPOZ increased to 42.60%"
This is not true, while two compounds 1,2-PhPOZ and 1,2-PhAc displayed a large PLQY post annulation, the other three reported compounds showed a weak improvement in PLQY, between 3 to 6%. The authors should adapt the discussion

Page 6 line 121-123: The authors wrote: "With the introduction of various solvents with increased polarity from n-hexane to acetonitrile, the maximum emission wavelengths (λem) of the 1,2-PhX series red-shifted gradually (Figure 2c–2d and Figure S21). Except for 1,2-PhPOZ, the other 1,2-PhX (X = PTZ, Ac, PTZO and PTZOO) showed a two-section linear fitting in low-polarity and high-polarity solvents, indicating the existence of two distinct excited states, according to the Lippert-Mataga solvatochromic model."

After reviewing Figure 2d and Figure S21 k)-o) I am not convinced that the presented data show a clear curvature or discontinuity that would necessitate a two-regime fit. In HLCT systems, the coexistence of LE and CT character is typically reflected by a smooth curvature in the solvatochromic plot rather than two imposed linear segments as shown in ref 35 (Adv. Funct. Mater. 2014, 24, 1609–1614).
In the case of this article the division into two linear regimes appears to rely on a selective choice of data points, and it is not clear that this segmentation is uniquely justified by the experimental data. For clarity and completeness, the authors need to include a conventional single-linear Lippert–Mataga fit using the full dataset, and to discuss how the conclusions compare with those obtained from the two-section fitting approach and why such a two-section fitting approach is absolutely needed.

Page 9 line 180-185. 1. The authors assignment of the pronounced temperature-dependent phosphorescence color shift to emission from higher-lying triplet states (T_n) is intriguing but, in its current form, not fully substantiated. In organic crystals, large spectral shifts upon cooling can also arise from reduced triplet exciton migration and suppression of defect- or trap-mediated emission, which is typically red-shifted. In addition, temperature-induced changes in exciton localization or subtle modifications of intermolecular packing may contribute without requiring a change in the emitting electronic state as shown in the following paper: : https://www.sciencedirect.com/science/article/pii/S0022231320316987
where blue shifted and enhanced phosphorescence from crystalline powder was observed upon temperature decrease.

More comprehensive temperature-dependent lifetime analysis at different temperatures not just at room temperature as shown in Fig.S40, would be valuable to highlight in more detail the mechanism.

2. Additionally the authors mentioned Figure S41 in the following sentence: ".[…] the exponential decay observed in time-

resolved emission spectroscopy (TRES) measurements (Figure S40 and S41)" which makes it look like Figure S41 focuses on TRES measurements which is not the case. The fact is the content of Figure S41 is not clearly discussed by the authors in the manuscript.

Page 12 line 237-239 the Authors reported: "the afterglow emission at short wavelengths (450 nm and 485 nm) initially showed exponential decay; subsequently, linear components appeared with a linear slope close to 1, indicating the existence of phosphorescence and TADF emission"
A few points are unclear about this sentence.
1. Why is the plot reported Figure 3h is in log vs log? This is an uncommon way to report decays usually shown is log (photon count) vs linear time (as explain above).
2. Why is the ordinate axis of the plot of Figure 3h described as Intensity (a.u.)? is it not photon counts like the previous decays reported?
3. In the case the ordinate is related to photon count and not an arbitrary intensity, the linear plot appears at extremely low counts (below 10).
The late-time decay component, which appears approximately linear on a log–log plot, emerges at photon count levels below ~10, where Poisson noise and background contributions are expected to dominate. In this regime, extracting decay slopes is statistically unreliable, and a linear dependence on a log–log scale merely reflects apparent power-law behavior (linear on log/log plot) rather than a specific emissive process. Consequently, the assignment of this component to phosphorescence and TADF emission is not sufficiently supported. Moreover, usual TADF and phosphorescence should be shown and analyzed on log/linear plots.

Page 13 line 264-268: The temperature-dependent enhancement of the ESA feature around 780 nm is intriguing and suggests thermally activated excited-state dynamics. However, attributing this behavior directly to a hot reverse intersystem crossing (hRISC) process may be premature. More discussion is necessary.

Minor
Figure 1 page 4: The definition of TADF vs hot-exciton as described by Y. Ma in his review: Chem. Soc. Rev., 2021,50, 1030-1069 states than TADF mainly involves S1 and T1 excited state in the ISC and RISC process. Ma and colleagues explain that involvement of higher energy excited state such as Sn and or Tn leads to the phenomenon of hot exciton. Can the authors share their opinion of choosing the term TADF in Figure 1b and c as these show Tn involved processes?

Figure 1d: 1. In this figure the authors decided to show TADF-Phosphorescence emission gap vs PLQY. The issue is that the TADF-Phosphorescence gap has been reported in units of nanometers which is not suitable to report energy gaps as wavelength is not linearly proportional to energy.
Such energy differences relevant to thermally activated processes should be reported in energy units (eV or cm^{-1}) to allow meaningful physical interpretation and comparison.

2. At the end of the manuscript such as page 13 line 244 the authors again use nanometer to highlight a wavelength shift which would be more appropriately underlined in a unit linearly proportional to energy.
Throughout the paper all discussions of these values should be change to eV.

Page 6 line 110 – Figure S17a: The author chose to show the absorption spectra of compounds in THF. THF absorption cut-off happens at 215nm, which means that data below the cut-off are not particularly relevant.

Page 8 Line 150-151: Two typos are present: "3.29s@425nm and 5.76s@420nm" should be written as "3.29s at 425nm" and "5.76s at 420nm"

Page 5 line 98: The phrase "proportions of (n, π) and (π,π*) characterization" is somewhat imprecise and need rephrasing such as:
"The introduction of different heteroatoms alters the orbital composition of the excited states, shifting their n→π versus π→π* character and consequently modulating intersystem crossing and radiative decay pathways."

Page 5 line 100: C-N coupling reaction is more commonly known as "Buchwald-Hartwig C-N coupling"

Figure 2 i): The authors show the evolution of the delayed emission of 1,2-PhAc as a function of temperature. In addition to the figure shown, it would be appreciable to visualize this evolution with normalized spectra even in a 1D plot in the SI.

ESI:

NMRs:
All numerical data for carbon NMR reported include the 3 peaks of the solvent: deuterated chloroform. The authors should not include these peaks when reporting the numerical NMR data.
Additionally, for molecule 1,2NpAc the signal of the quaternary carbon at 32.17ppm visible on the spectra reported below, is missing from the description of carbon peaks. It should be added.

Synthesis
The synthetic route of 1,2-PhPTZO and 1,2-PhPTZOO
I assume that the authors obtain a mixture of 1,2-PhPTZO and 1,2-PhPTZOO at the end of the procedure.
As such I would appreciate if the author could add the Rf values for both compound along with order of elution. Adding Rf

and elution order for other compounds would be nice but not mandatory as in this case where, to my understanding, both are separated from the same reaction mixture.

The synthetic route of 1,2-NpAc and 2,3-NpAc
“The crude product was purified by column chromatography on silica gel using petroleum ether and dichloromethane as eluents.”
I would appreciate if the authors could describe exactly the proportion of each solvent depending on the molecule or if they were used as gradients. Can be added to the characterization part.

Version 1:

Reviewer comments:

Reviewer #1

(Remarks to the Author)
The authors response positively to the comments. Now the manuscript is ready for publication.

Reviewer #2

(Remarks to the Author)
This review is my second for this submission and is a review of the paper following changes made in response to comments from myself and other reviewers during initial review.

I thank the authors for their detailed responses along with their changes to the manuscript and supplemental data, which have addressed all of my comments along with those of the other reviewer.

I now believe that this paper is ready for acceptance.

Open Access This Peer Review File is licensed under a Creative Commons Attribution 4.0 International License, which permits use, sharing, adaptation, distribution and reproduction in any medium or format, as long as you give appropriate credit to the original author(s) and the source, provide a link to the Creative Commons license, and indicate if changes were made.
In cases where reviewers are anonymous, credit should be given to 'Anonymous Referee' and the source.
The images or other third party material in this Peer Review File are included in the article’s Creative Commons license, unless indicated otherwise in a credit line to the material. If material is not included in the article’s Creative Commons license and your intended use is not permitted by statutory regulation or exceeds the permitted use, you will need to obtain permission directly from the copyright holder.
To view a copy of this license, visit https://creativecommons.org/licenses/by/4.0/

Manuscript ID: NCOMMS-25-87645

MS Title: Modulating High-Energy Triplet State Transition Pathways by Twisted Hetero-annulation Strategy with Temperature as "Control Knobs"

MS Authors: Guigui Ye, Yan Gao, Wentao Yuan, Juqing Gu, Jiaqiang Wang, Yujie Yang, Qianqian Li, Zhen Li

As to Reviewer (R) 1,

"This manuscript entitled Modulating High-Energy Triplet State Transition Pathways by Twisted Hetero-annulation Strategy with Temperature as "Control Knobs" presents very interesting observations in organic afterglow systems with diverse photophysical behaviors. A series of organic compounds have been studied, some of which exhibit unusual afterglow property. The observation of TADF-type afterglow with large ΔE_{ST} is rare. Most of the properties are well interpreted either by experiments or by theoretical calculations. There are some comments attached in the following. This manuscript can be accepted for publication after revisions."

(R) 1. "Regarding the title, twisted hetero-annulation seems not to be a strategy, so the word strategy may be removed. with temperature as "control knobs" seems to be difficult to follow. Does it mean controlled by temperature? A clear title may be better for this manuscript and for the readers."

Thanks for your comments. Accordingly, the manuscript title has been clarified and revised as "High Energy Triplet-State Manipulation *via* Temperature-Responsive Twisted Hetero-Annulation Systems".

(R) 2. "In page 3, the sentence "3 pi-pi transition in T_n states, which can slow down the decay rates for the spin-forbidden transitions ...favoring the stabilization of T_n states" make sense. On the other hand, the T_n-T_1 internal conversion would quench or consume T_n states rapidly. This should be considered in the discussion. The emergence of TADF-type afterglow with such large ΔE_{ST} needs further supports."

Thanks for your comments. We agree that internal conversion from higher-lying triplet states (T_n) to T_1 is generally efficient and would be expected to rapidly depopulate T_n states. This point has now been explicitly considered as follows:

$$K_{IC} = \frac{2\pi}{\hbar}\sum_{\{wj\}} |\langle T_1\{wj\}|\hat{V}|T_n\{0\}\rangle|^2\ \delta(E_f\text{-}E_i) \qquad \text{Equation 1}$$

According to Fermi's golden rule, the internal conversion rate (K_{IC}) can be qualitatively described by the general form of Equation 1. These relations show that K_{IC} is governed not only by the energy gap ($\delta(E_f\text{-}E_i)$), but

also by the nonadiabatic vibronic coupling between the involved states ($\langle T_1\{wj\}|\hat{V}|T_n\{0\}\rangle$). Thus, the effective suppression of T_n-T_1 internal conversion can be achieved by reducing molecular vibrations and enlarging the T_n-T_1 energy gap.

The rigid π-conjugated structure of 1,2-NpAc, together with strong intermolecular interactions in the crystalline state, imposes significant constraints on molecular motions. This confinement weakens nonadiabatic coupling and thereby suppresses internal conversion. In addition, the relatively large energy gap between T_n and T_1 states (e.g., ~1.06 eV between T_{10} and T_1, as estimated by TD-DFT for the trimer, where T_{10} is selected from the lowest triplet state satisfying S_1-T_n < 0.3 eV to enable efficient ISC) further impedes the internal conversion pathway. As a result, nonradiative decay among triplet excited states is largely suppressed in the 1,2-NpAc crystal.

At the same time, the high-level reverse intersystem crossing (*h*RISC) can be promoted by the small energy gap (only 0.06 eV, as estimated by TD-DFT for the trimer) between T_{10} and S_1. This delicate balance effectively suppresses internal conversion within the triplet manifold while enabling higher triplet states to drive delayed fluorescence through the $T_n \rightarrow S_1$ *h*RISC channel, thereby bypassing the conventional $T_1 \rightarrow S_1$ pathway.

According to the comments, we have expanded the discussion on this point in the revised manuscript to improve clarity.

Revised Text in the Manuscript:

"*Notably, the energy gap between T_{10} and S_1 is only 0.06 eV, accompanied by a large SOC value of 0.2087 cm-1 (Figure 4f) (T_{10} is selected from the lowest triplet state satisfying S_1–T_n < 0.3 eV to enable efficient ISC). Although $T_n \rightarrow T_1$ IC process is generally efficient, its rate is governed by both the nonadiabatic vibronic coupling and the T_n − T_1 energy gap. In the crystalline state, the rigid π-conjugated structure and strong intermolecular interactions effectively restrict molecular motions, thereby weakening vibronic coupling, while the large T_{10} − T_1 gap (~1.06 eV by calculation) further suppresses IC process. Meanwhile, the small T_{10} − S_1 gap facilitates hRISC, allowing T_n states to repopulate S_1. This balance suppresses nonradiative decay of T_n excitons while promoting T_n-mediated DF-type afterglow via the $T_n \rightarrow S_1$ pathway.*" (page 17, *line 337-345* in the revised manuscript).

***(R) 3.* "For the terms stabilize and stabilization, these would also refer to the lowering of energy levels of excited states. From the context, the authors use these terms to describe the protection of excited states or the**

suppression of nonradiative decay. These terms may be revised to avoid misunderstanding."

Thank you for your comments. Accordingly, we have replaced the terms "stabilize" and "stabilization" with "suppression of nonradiative decay" throughout the manuscript to enhance clarity and prevent ambiguity.

(R) 4.* "T_n *emission is very rare. The assignment of* T_n *emission needs further support or ruling out other possible mechanism."

Thank you for your comment. We agree that direct emission from T_n state is indeed rare in organic systems, and therefore requires careful justification and exclusion of alternative mechanisms. In this work, we do not assign the short-wavelength emission at 450 nm and 485 nm to direct radiative decay from T_n to S_0. Instead, we propose that higher-lying triplet states act as intermediate states that facilitate delayed fluorescence *via* the *h*RISC process from $T_n \rightarrow S_1 \rightarrow S_0$. Therefore, T_n acts as a transient intermediate that serves to mediate and accelerate the RISC process in this system.

Experimentally, delayed emission spectra recorded with 1 ms delay exhibit four peaks at 450 nm, 485 nm, 620 nm, and 680 nm, with lifetimes of 24.08 ms, 23.41 ms, 39.98 ms, and 37.91 ms, respectively. The prompt photoluminescence (PL) and delayed emission spectra show overlapping emission bands at 450 nm and 485 nm (Figure R1a), indicating that these features originate from the same emissive singlet state. Temperature-dependent measurements (Figure R1b–c) further support this assignment: the intensities of the short-wavelength emissions at 450 nm and 485 nm increase markedly as the temperature rises from 220 K to 340 K, followed by a slight decrease at higher temperatures (up to 380 K), a behavior characteristic of delayed fluorescence (DF). In contrast, the long-wavelength emissions at 620 nm and 680 nm decrease monotonically with increasing temperature, consistent with thermally quenched phosphorescence.

The energy gap estimated from the DF (450 nm) and phosphorescence (620 nm) emission wavelengths is approximately 0.76 eV, which significantly exceeds the typical energy gap required for efficient T_1-mediated reverse intersystem crossing (RISC). According to the Arrhenius equation, such a large energy gap would result in an extremely low RISC rate (on the order of 10^{-13} s^{-1}), effectively ruling out a conventional T_1-mediated DF mechanism. Instead, these processes are likely mediated by T_n states, which serve as intermediate "staging posts" in the upconversion pathway. The efficiency of these transitions is strongly governed by molecular packing and intermolecular interactions in the crystalline state. Single-crystal structural analysis reveals the presence of multiple short-range interactions, including C–H···π interactions with distances ranging from 2.683 to 2.930 Å and π–π interactions with a centroid-to-centroid distance of 3.991 Å. Such noncovalent interactions

effectively restrict molecular vibrations and suppress nonradiative decays, particularly IC process from T_n to T_1. Concurrently, intermolecular electronic coupling arising from compact molecular packing enhances SOC, thereby facilitating *h*RISC from T_n to S_1. This $T_n \rightarrow S_1$ upconversion process competes effectively with $T_n \rightarrow T_1$ conversion, ultimately giving rise to dual delayed emission: DF arising from the $T_n \rightarrow S_1 \rightarrow S_0$ pathway and phosphorescence originating from the $T_n \rightarrow T_1 \rightarrow S_0$ pathway.

Moreover, the possibility of defect-related emission was excluded by the similar excitation spectra obtained for emissions at 450 nm, 485 nm, 620 nm, and 680 nm (Figure R1d). Likewise, the minimal structural variations across the investigated temperature range ruled out emission modulation arising from temperature-dependent changes in molecular packing (Figure R1e–f). Collectively, these spectroscopic tests, together with the analyses of temperature-dependent exciton dynamics, crystal structures, and theoretical calculations, provide strong support for T_n-mediated DF mechanism.

According to your comments, we have expanded the discussion on this point in the revised manuscript to improve clarity. Figures R1a and 1d were added to the revised supporting information as Figure S53 and Figure S60, respectively.

Revised Text in the Manuscript:

"*Upon aggregation into the crystalline state, the 1,2-NpAc crystal exhibits dual-emission afterglow consisting of both DF and phosphorescence. Quantitatively, the energy gap between DF and phosphorescence is measured to be 0.76 eV, substantially larger than 0.3 eV. This observation suggests that efficient RISC is unlikely to occur directly between* T_1 *and* S_1 *via the conventional pathway. Instead, these processes are likely mediated by* T_n *states, which serve as intermediate "staging posts" in the upconversion pathway. The efficiency of these transitions is strongly governed by molecular packing and intermolecular interactions in the crystalline state (Table S21). Single-crystal structural analysis reveals the presence of multiple short-range interactions, including C–H···π interactions with distances ranging from 2.683 to 2.930 Å and π–π interactions with a centroid-to-centroid distance of 3.991 Å. Such noncovalent interactions effectively restrict molecular vibrations and suppress nonradiative decays, particularly IC process from* T_n *to* T_1*. Concurrently, intermolecular electronic coupling arising from compact molecular packing enhances SOC (Figure S56 and Table S22), thereby facilitating hRISC from* T_n *to* S_1*. This* $T_n \rightarrow S_1$ *upconversion process competes effectively with* $T_n \rightarrow T_1$ *conversion, ultimately giving rise to dual delayed emission: DF arising from the* $T_n \rightarrow S_1 \rightarrow S_0$ *pathway and phosphorescence originating from the* $T_n \rightarrow T_1 \rightarrow S_0$ *pathway*" (page 14, *line 291-300, and* page 15, *line 301-304* in the revised manuscript)

"*Thus, emission modulation due to temperature-induced changes in molecular packing can be discounted, as the crystal structure exhibits only negligible variations across the studied temperature range.*" (page 17, *line 355-356* in the revised manuscript)

"*Additionally, the possibility of defect-related emission was excluded by the similar excitation spectra obtained for emissions at 450 nm, 485 nm, 620 nm, and 680 nm (Figure S60). Collectively, these spectroscopic tests, together with the analyses of temperature-dependent exciton dynamics, crystal structures, and theoretical calculations, provide strong support for T_n-mediated DF and phosphorescence mechanism.*" (page 18, *line 361-365* in the revised manuscript)

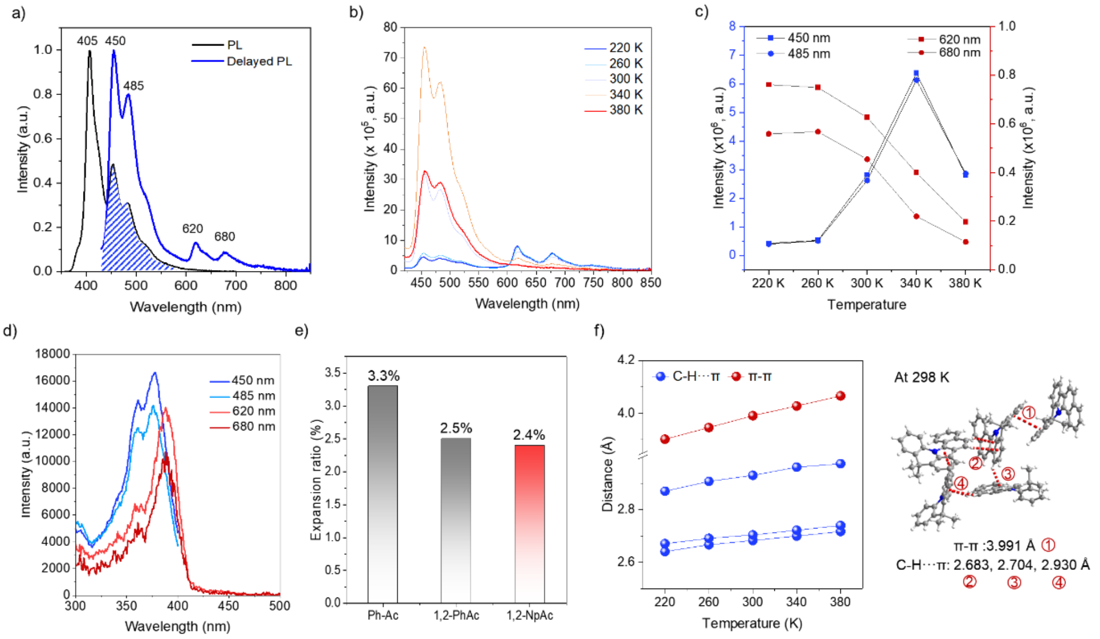

Figure R1. a) The prompt photoluminescence (PL) spectra and delayed emission spectra (1 ms delay) of 1,2-NpAc crystals. b) Delayed emission spectra (1 ms delay) of 1,2-NpAc crystals at different temperatures ranging from 220 K to 380 K. c) Temperature-dependent intensity evolution of the delayed emission monitored at 450 nm, 485 nm, 620 nm and 680 nm over the range of 220 K – 380 K. d) Excitation spectra of 1,2-NpAc crystals recorded at emission wavelengths of 450 nm, 485 nm, 620 nm, and 680 nm. e) The cell volume expansion ratios for Ph-Ac, 1,2-PhAc, and 1,2-NpAc crystals with the temperature increasing from 220 K to 380 K. f) The changes of intermolecular distances with C-H···π and π-π interaction in 1,2-NpAc crystal across the temperature range of 220 K – 380 K and corresponding intermolecular interactions in 1,2-NpAc crystal at 298 K.

As to Reviewer (R) 2,

"The authors report a series of organic compounds designed to investigate how molecular rigidification, achieved through annulation, and π-system extension influence luminescent properties via modulation of singlet and triplet excited states. The photophysical behavior is first examined in solution (THF) and subsequently in the solid state, with particular emphasis on crystalline samples and the temperature dependence of their emission properties. Building on the insights gained from the initial molecule set on delayed luminescence, the study further explores how extending the π-conjugated framework impacts excited-state dynamics and emission characteristics.

The control and manipulation of excited states—particularly triplet states—represent an important and timely topic with clear relevance for the development of advanced luminescent materials and sensing applications. While the overall approach and objectives of the study are of significant interest, several aspects of the analysis and interpretation require further clarification and deeper discussion before the work can be considered for publication. I believe this paper will need re-review."

(R) 1. "The authors attribute the observed temperature-dependent emission behavior primarily to hot-exciton–mediated processes, including hISC and hRISC, supported by DFT/TDDFT calculations and experimental observations. While this interpretation is plausible, the experimental results suggest that such behavior may not be an intrinsic molecular property alone, but rather strongly dependent on the solid-state environment. The authors should clarify the assignment of each of the processes they observe to unambiguously discuss their observation. Currently there is a lack of clarity on which each process is assigned to which observation. In particular, in dilute solution or especially in resin matrices (Figure S53)—where intermolecular interactions and excitonic coupling are minimized—the compounds seem to exhibit relatively conventional photophysical behavior, without clear signatures of hot-exciton–driven emission. Even if the calculation results seem to indicate involvement of T_n state in ISC, the energy of their predicted T_1 can still be linked with emission around 450 nm. While afterglow can be observed in resin no temperature induced color-changed can be seen and I wonder if TADF type emission is observed for compound 1,2-NpAc. In contrast, the most striking phenomena, such as temperature-dependent color switching and pronounced phosphorescence, only emerge in the crystalline state. This raises the question of whether the observed effects originate primarily from intrinsic hot-exciton dynamics of individual molecules, or whether they arise from collective phenomena such as crystal packing, exciton delocalization, or rigidification-induced suppression of nonradiative decay. In this context, the role of the crystal lattice and intermolecular interactions may be

as critical as the electronic structure of the isolated chromophores themselves. The authors must discuss such possibilities in order to allow for a complete interpretation of their observed properties."

Thanks for your comments. We fully agree that a clear assignment of the observed photophysical phenomena to their underlying excited-state processes is essential. In response, we have carefully re-examined both the experiments and theoretical results across different environments. Below, we address the reviewer's comments point by point, focusing on **(i)** the role of intrinsic excited-state characteristics *versus* crystalline environment and **(ii)** the clear assignment of each observed afterglow.

(i) The role of intrinsic excited-state characteristics *versus* crystalline environment

Based on systematic investigation of the photophysical properties of 1,2-NpAc in isolated states (*e.g.*, in dilute solution and resin matrix) and in the crystalline state, we conclude that the temperature-dependent switching of the afterglow color arises not only from intrinsic molecular properties, but also from electronic coupling in the crystalline environment.

For 1,2-NpAc in its isolated state, the delayed emission spectrum of the compound embedded in epoxy resin at room temperature closely resembles that recorded in dilute THF solution at 77 K, featuring emission peaks at 500 nm, 535 nm, and 575 nm (Figures R2a), respectively. As the temperature increased, the emission wavelengths showed negligible changes, and the corresponding intensities and lifetimes decreased monotonically, consistent with phosphorescence emission (Figure R2b-2e). The singlet–triplet energy gap is approximately 0.38 eV (Figure R2f). As an intrinsic feature of the excited-state manifold, this substantial energy gap opens up the possibility of realizing T_n-mediated ISC processes.

In the crystalline state, 1,2-NpAc exhibits distinct temperature-dependent afterglow color switching, which originates from the distinct responses of its short- and long-wavelength emission peaks to temperature variation. Upon increasing the temperature from 220 K to 340 K, the short-wavelength delayed emission bands at 450 nm and 485 nm progressively intensify, whereas the long-wavelength bands at 620 and 680 nm concomitantly weaken. This opposing trend indicates that different emission mechanisms govern the short- and long-wavelength regions. A combined analysis of steady-state spectra, TRES measurements, and temperature-dependent responses assigns the short-wavelength emission to delayed fluorescence and the long-wavelength emission to phosphorescence. The energy gap between delayed fluorescence (450 nm) and phosphorescence (620 nm) is approximately 0.76 eV, which substantially exceeds the typical energy separation required for efficient RISC in conventional TADF systems. This large energy difference effectively precludes a direct $T_1 \rightarrow S_1$ upconversion pathway. Instead, the upconversion mechanism most likely involves T_n states, acting as

intermediate states. This hypothesis is further supported by analysis of the crystal structure and density functional theory (DFT) calculations.

Single-crystal X-ray diffraction analysis reveals a network of intermolecular C–H···*π* (2.683–2.930 Å) and *π*–*π* (3.991 Å) interactions, which induce lattice rigidification and intermolecular electronic coupling. The resultant rigid environment restricts large-amplitude molecular vibrations and attenuates vibronic coupling, effectively suppressing $T_n \rightarrow T_1$ internal conversion. Simultaneously, the intermolecular orbital overlap enhances SOC (Figure R3), thereby providing additional spin-flip channels that facilitate the $T_n \rightarrow S_1$ *h*RISC process. Thus, the rigid crystalline environment impedes $T_n \rightarrow T_1$ internal conversion, leading to transient T_n population. Assisted by enhanced SOC from intermolecular electronic coupling, thermally activated $T_n \rightarrow S_1$ RISC subsequently repopulates S_1, resulting in short-wavelength delayed fluorescence.

Consequently, cooling suppresses thermal activation pathways, redirecting excited-state relaxation primarily *via* $T_n \rightarrow T_1 \rightarrow S_0$ to produce phosphorescence at 620 and 680 nm. Intermolecular π–π interactions within the crystal account for the redshift of this emission relative to that of isolated 1,2-NpAc (λ_{max} = 575 nm). Thus, the observed afterglow color switching in the crystalline state arises from temperature-dependent competition between dual excited-state decay channels from the T_n states: delayed fluorescence ($T_n \rightarrow S_1 \rightarrow S_0$) and phosphorescence ($T_n \rightarrow T_1 \rightarrow S_0$).

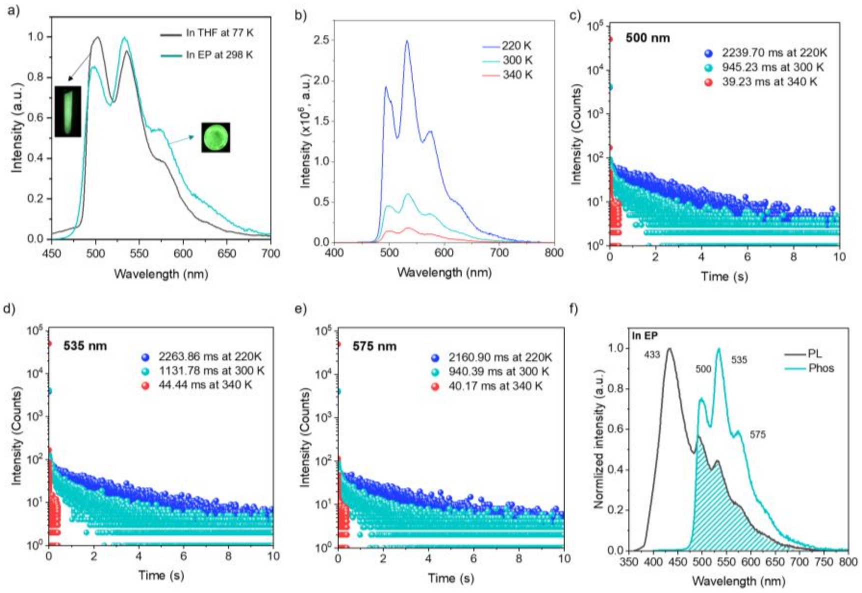

Figure R2. a) The delayed emission spectra (1 ms delay) of 1,2-NpAc in THF at 77 K and in epoxy resin (EP) at 298 K. b) Delayed emission spectra (1 ms delay) at different temperatures. The delayed emission decay profiles of 1,2-NpAc@epoxy resin monitored at c) 500 nm, d) 535 nm and e) 575 nm. f) The prompt photoluminescence (PL) spectra and phosphorescence spectra of 1,2-NpAc@epoxy resin.

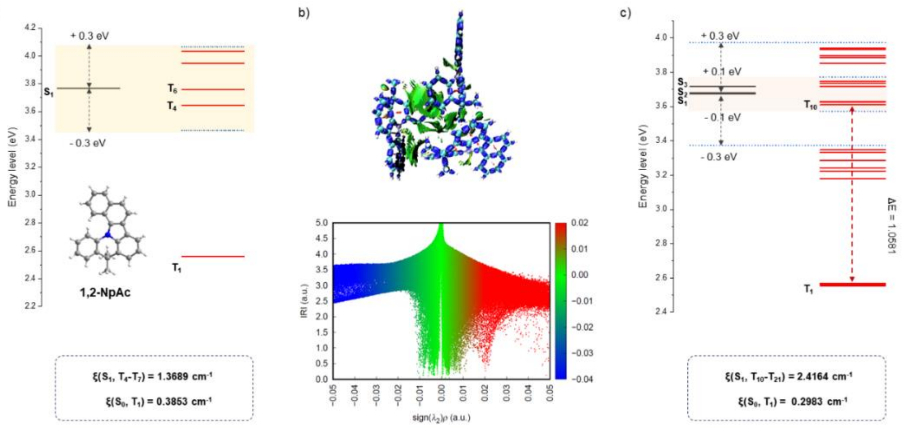

Figure R3. a) Calculated SOC constants and energy level of 1,2-NpAc at isolated state. b) Gradient isosurfaces and the corresponding scatter diagrams of 1,2-NpAc trimer for IRI versus sign $(\lambda_2)\rho$. c) Calculated SOC constants and energy gaps of 1,2-NpAc trimer. (The molecular geometries were extracted from the corresponding single crystals).

(ii) The clear assignment of each observed afterglow

By systematically investigating the emission spectra of 1,2-NpAc in isolated and crystalline states, we establish a definitive correlation between its photophysical properties and excited-state dynamics. Integrating steady-state, time-resolved, and temperature-dependent spectroscopy enables the clear assignment of each observed afterglow to either T_n-mediated DF or phosphorescence, directly elucidating the environmental modulation of the excited-state transition channels.

a) In isolated state (delayed emission wavelength: 500 nm, 535 nm, and 575 nm)

Long-lived emission bands are observed at 500 nm, 535 nm, and 575 nm in THF solution (77 K) and in epoxy resin matrix (room temperature), corresponding to phosphorescence.

b) In crystal state (delayed emission wavelength: 450 nm, 485 nm, 620 nm and 680 nm)

The emission bands centered at 450 nm and 485 nm can be unambiguously assigned to the T_n-mediated DF process involving $S_1 \rightarrow T_n \rightarrow S_1 \rightarrow S_0$ transitions. This assignment is substantiated by multiple lines of evidence: (i) their spectral positions coincide with those of the prompt fluorescence; (ii) their intensities increase with rising temperature, characteristic of thermally activated processes; (iii) their decay profiles are multi-exponential; and (iv) the large energy gap (~0.76 eV) effectively precludes direct $T_1 \rightarrow S_1$ upconversion. These observations collectively implicate the T_n states as the key mediators of the observed DF. Meanwhile, the peaks at 620 nm and 680 nm are assigned to $S_1 \rightarrow T_n \rightarrow T_1 \rightarrow S_0$ phosphorescence.

The T_n-mediated afterglow of the 1,2-NpAc crystal arises from two contributing factors. Intrinsically, the

compound possesses a large singlet–triplet energy gap that enables potential T_n-mediated transitions. Extrinsically, the rigid crystal structure and intermolecular electronic coupling simultaneously suppress $T_n \rightarrow T_1$ conversion and promote the $T_n \rightarrow S_1$ *h*RISC process, leading to delayed fluorescence at room temperature. π–π coupling in the crystal further enlarges the energy gap between delayed fluorescence and phosphorescence by lowering the T_1 energy level, thereby enabling the blue-red afterglow switching with a significant color change.

According to the comments, the corresponding discussions have been added to the revised manuscript, and Figures R2a-e, R2f and R3 were added to the supporting information as Figure S50, Figure S55 and Figure S56, respectively.

Revised Text in the Manuscript:

"*Systematic photophysical investigations in both isolated and crystalline states reveal that the high-contrast, temperature-dependent afterglow color change of 1,2-NpAc arises from the combined effects of intrinsic excited-state characteristics and electronic coupling in the crystalline state. In the isolated state, 1,2-NpAc exhibits only phosphorescence. Its pronounced (π, π*) character by naphthyl-fused conjugation leads to a relatively large singlet–triplet energy gap (~0.38 eV) (Figure S55), while the twisted π-conjugated structure enhances SOC, enabling potential T_n-mediated transitions from $S_1 \rightarrow T_n \rightarrow T_1 \rightarrow S_0$. This establishes an intrinsic basis for efficient triplet exciton utilization.*

Upon aggregation into the crystalline state, the 1,2-NpAc crystal exhibits dual-emission afterglow consisting of both DF and phosphorescence. Quantitatively, the energy gap between DF and phosphorescence is measured to be 0.76 eV, substantially larger than 0.3 eV. This observation suggests that efficient RISC is unlikely to occur directly between T_1 and S_1 via the conventional pathway. Instead, these processes are likely mediated by T_n states, which serve as intermediate "staging posts" in the upconversion pathway. The efficiency of these transitions is strongly governed by molecular packing and intermolecular interactions in the crystalline state (Table S21). Single-crystal structural analysis reveals the presence of multiple short-range interactions, including C–H···π interactions with distances ranging from 2.683 to 2.930 Å and π–π interactions with a centroid-to-centroid distance of 3.991 Å. Such noncovalent interactions effectively restrict molecular vibrations and suppress nonradiative decays, particularly IC process from T_n to T_1. Concurrently, intermolecular electronic coupling arising from compact molecular packing enhances SOC (Figure S56 and Table S22), thereby facilitating hRISC from T_n to S_1. This $T_n \rightarrow S_1$ upconversion process competes effectively with $T_n \rightarrow T_1$ conversion, ultimately giving rise to dual delayed emission: DF arising

from the $T_n \rightarrow S_1 \rightarrow S_0$ pathway and phosphorescence originating from the $T_n \rightarrow T_1 \rightarrow S_0$ pathway. π–π coupling in the crystal further enlarges the energy gap between delayed fluorescence and phosphorescence by lowering the T_1 energy level, thereby enabling the blue-red afterglow switching with a significant color change." (page 14, *line 284-300* and page 15, *line 301-307* in the revised manuscript)

***(R) 2.* "The presentation of time-resolved data needs greater consistency. In the current manuscript, decay curves are shown using a mixture of logarithmic–linear and log–log (ex: Figure S40, Figure 3h), sometimes with the ordinate described as intensity in arbitrary units and other times in photon counts. This variability complicates direct comparison between datasets. A consistent representation—preferably using logarithmic photon counts versus linear time, which is standard for phosphorescence and TADF analysis—is necessary to improve clarity and facilitate comparison of decay dynamics across samples and temperatures."**

Thanks for your comments. We fully agree that a consistent representation of time-resolved decay curves is essential for direct comparison of decay dynamics across samples and temperatures. Accordingly, in the revised manuscript, all conventional time-resolved photoluminescence decay curves have been reprocessed and are now presented in a semi-logarithmic format (logarithmic photon counts *versus* linear time), which is the standard representation for analyzing phosphorescence and DF lifetimes. This unified treatment ensures direct comparability of decay kinetics throughout the manuscript and Supporting Information.

To analyze the excited-state relaxation regimes across an exceptionally wide temporal window, the data presented for the 1,2-NpAc crystal in Figure 3h and Figure S40 (Figure S43 in revised SI) were constructed from time-resolved emission spectra (TRES), rather than conventional time-resolved photoluminescence decay curves. Specifically, each data point represents the emission intensity extracted at a given wavelength and time delay. A log–log representation was deliberately adopted to facilitate visualization of multiple dynamical regimes within the same timeframe, a format well-established in the literature for complex excited-state systems involving competing emissive pathways (*Adv. Opt. Mater.* 2022, 10, 2101784; *J. Am. Chem. Soc.* 2023, 145, 25495), as illustrated in Figure R4.

Accordingly, to enable consistent lifetime comparison, all conventional time-resolved photoluminescence decay curves are now presented in a semi-logarithmic format. For Figure 3h and Figure S40 (Figure S43 in revised SI), the log–log representation is retained to better capture the intricate excited-state dynamics.

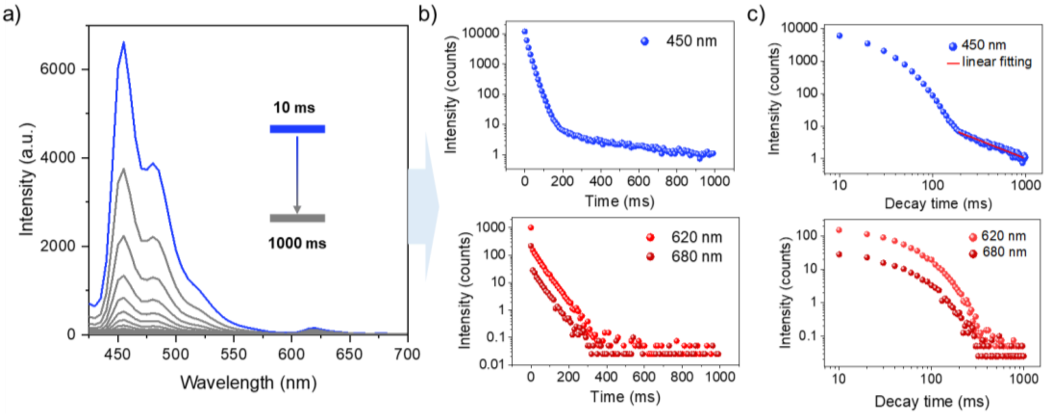

Figure R4. a) Time-resolved emission spectra (TRES) of 1,2-NpAc crystals. b) Time-resolved emission decay kinetics of 1,2-NpAc crystals at 450 nm, 620 nm, and 680 nm plotted on a logarithmic – linear scale. c) Time-resolved emission decay kinetics of 1,2-NpAc crystals at 450 nm, 620 nm, and 680 nm plotted on a log – log scale.

<u>(R) 3.</u> "Moreover, in some cases the reported photon counts appear to be close to the noise level (below 100). which complicates the interpretation of the decay behaviour. For clarity and reproducibility, all decay traces including the corresponding fitting curves and χ^2 values should be presented in the SI."

Thanks for your comments. Accordingly, we compared the sample decay traces with the corresponding background signals (blank) (Figure R5). The measured intensities consistently exceed the background level across the entire temporal range, confirming that the observed decay features are sample-derived and clearly distinguishable from instrumental noise. Figures R5 was added to the supporting information as Figure S52.

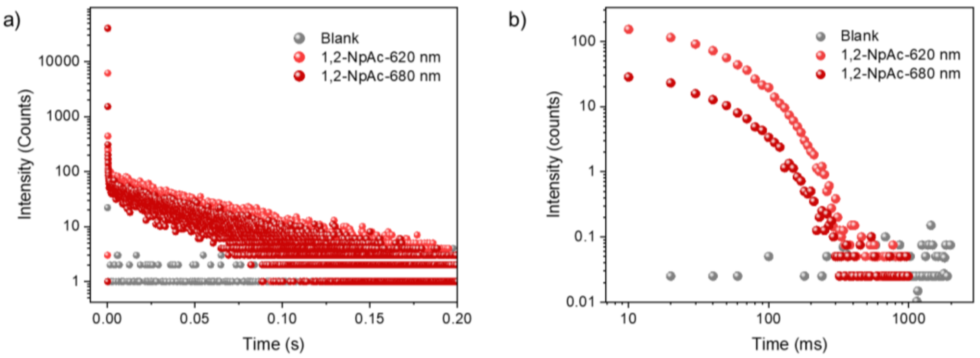

Figure R5. a) Delayed emission decay profiles of 1,2-NpAc crystals monitored at 620 and 680 nm, and the background (blank). b) Time-resolved emission decay kinetics of 1,2-NpAc crystals monitored at 620 and 680 nm, and the background (blank) from TRES, plotted on a log – log scale.

In response to the reviewer's comment regarding clarity and reproducibility, we have now incorporated fitting curves and corresponding χ^2 values for all decay traces in the manuscript. Except for the compounds measured in THF at 77 K using an F-4700 spectrometer, the χ^2 values were derived using the same proprietary fitting software (Edinburgh Instruments FLS980 spectrometer) under identical protocols to ensure consistency. Figure R6 provides a representative example, showing the software interface along with a decay trace and its fitting curve to illustrate the fitting quality. The complete dataset is summarized in Tables R1 – R6.

According to the comments, the corresponding discussions have been added to the revised manuscript, and Tables R1-R5 were added to Table S6, S14, S17, S19-S20 in SI, respectively.

Revised Text in the Manuscript:

"Comparison with background signals confirms that the decay traces originate from the sample rather than instrumental noise." (Page 13, *line 256 – 257* in the revised manuscript)

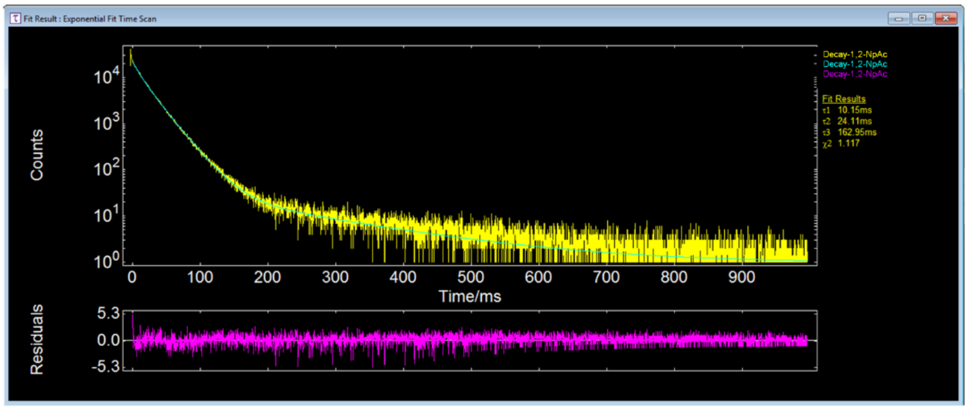

Figure R6. Fitting delayed emission decay curves of 1,2-NpAc crystal monitored at 450 nm by Edinburgh Instruments FLS980 spectrometer.

Table R1. Exponential fitting parameters of the delayed emission decay in THF at 77 K

Sample	τ_1 (s)	Rel.%	τ (s)	χ^2
Ph-POZ@455 nm	1.66	100	1.66	0.999
Ph-POZ@465 nm	1.79	100	1.79	0.999
Ph-POZ@487 nm	1.69	100	1.69	0.999
1,2-PhPOZ@435 nm	2.38	100	2.38	0.999
1,2-PhPOZ@465 nm	2.41	100	2.41	0.998
1,2-PhPOZ@487 nm	2.41	100	2.41	0.999
Ph-PTZ@504 nm	0.07	100	0.07	0.988
Ph-PTZ@535 nm	0.08	100	0.08	0.989
1,2-PhPTZ@490 nm	0.15	100	0.15	0.998
1,2-PhPTZ@515 nm	0.15	100	0.15	0.999
Ph-Ac@425 nm	3.29	100	3.29	0.999

1,2-PhAc@420 nm	5.18	100	5.18	0.998
1,2-PhAc@450 nm	5.46	100	5.46	0.987
1,2-PhAc@480 nm	4.12	100	4.12	0.980
Ph-PTZO@435 nm	0.15	100	0.15	0.999
1,2-PhPTZO@430 nm	1.45	100	1.45	0.997
1,2-PhPTZO@460 nm	1.44	100	1.44	0.997
Ph-PTZOO@430 nm	1.45	100	1.45	0.999
Ph-PTZOO@460 nm	1.44	100	1.44	0.999
1,2-PhPTZOO@430 nm	2.01	100	2.01	0.996
1,2-PhPTZOO@460 nm	1.97	100	1.97	0.998
1,2-PhPTZOO@490 nm	2.07	100	2.07	0.998

Table R2. Multiexponential fitting parameters of the delayed emission decay in crystal state

Sample	τ_1 (ms)	Rel.%	τ_2 (ms)	Rel.%	τ_3 (ms)	Rel.%	τ (ms)	χ^2
Ph-POZ@492 nm	0.09	9.96	2.43	90.04	-	-	2.19	0.969
Ph-POZ@530 nm	0.15	30.53	1.96	69.47	-	-	1.41	0.923
1,2-PhPOZ@490 nm	0.15	15.68	2.45	54.67	23.31	29.64	8.27	1.099
1,2-PhPOZ@610 nm	0.01	0.28	3.51	56.87	19.37	23.76	0.89	1.155
Ph-PTZ@540 nm	1.75	60.42	5.21	39.58	-	-	3.12	0.997
1,2-PhPTZ@462 nm	4.77	90.77	19.56	9.23	-	-	6.14	1.016
1,2-PhPTZ@496 nm	3.45	60.27	8.92	39.73	-	-	5.62	0.989
1,2-PhPTZ@530 nm	3.02	51.75	9.08	48.25	-	-	5.95	1.041
Ph-Ac@505 nm	36.92	49.81	277.56	50.19	-	-	157.70	1.032
1,2-PhAc@590 nm	158.21	768.54	52.97	47.03	-	-	445.25	1.065
Ph-PTZO@525 nm	18.62	48.10	79.97	20.03			24.52	0.997
1,2-PhPTZO@495 nm	19.33	80.43	161.98	19.57	-	-	47.25	1.057
1,2-PhPTZO@535 nm	10.45	73.92	30.66	26.08	-	-	15.72	0.964
Ph-PTZOO@530 nm	35.84	330.6	71.68	28.32	-	-	119.32	1.031
1,2-PhPTZOO@580 nm	16.71	153.04	43.45	56.55	-	-	93.80	1.075

Table R3. Multiexponential fitting parameters of the delayed emission decay in THF at 77 K

Sample	τ_1 (s)	Rel.%	τ (s)	χ^2
1,2-NpAc@500 nm	2.72	100	2.72	0.999
1,2-NpAc@535 nm	2.72	100	2.72	0.999
1,2-NpAc@580 nm	2.71	100	2.71	0.999
2,3-NpAc@538 nm	1.89	100	1.89	0.999
2,3-NpAc@585 nm	1.60	100	1.60	0.990
2,3-NpAc@640nm	1.65	100	1.65	0.999

Table R4. Multiexponential fitting parameters of the prompt emission decay in epoxy resin across

	τ_1 (ms)	Rel.%	τ_2 (ns)	Rel.%	τ (ms)	χ^2
1,2-NpAc@500 nm-340 K	3.22	29.18	54.07	70.82	39.23	1.017
1,2-NpAc@500 nm-300 K	945.23	100	-	-	945.23	0.995
1,2-NpAc@500 nm-220 K	2239.7	100	-	-	2239.70	1.078
1,2-NpAc@535 nm-340 K	3.15	23.72	57.28	76.28	44.44	1.149
1,2-NpAc@535 nm-300 K	158.56	20.42	1381.50	79.58	1131.78	0.939
1,2-NpAc@535 nm-220 K	287.19	1.47	2293.35	98.53	2263.86	1.080
1,2-NpAc@575 nm-340 K	3.44	28.84	55.06	71.16	40.17	1.037
1,2-NpAc@575 nm-300 K	125.35	21.42	1162.56	78.58	940.39	0.991
1,2-NpAc@575 nm-220 K	239.75	2.54	2210.97	997.46	2160.90	1.100

Table R5. Multiexponential fitting parameters of the delayed emission decay in the crystal state

	τ_1 (ms)	Rel.%	τ_2 (ms)	Rel.%	τ_3 (ms)	Rel.%	τ (ms)	χ^2
1,2-NpAc@450 nm-220 K	3.75	41.46	24.81	31.10	115.78	27.44	41.04	1.050
1,2-NpAc@450 nm-260 K	2.32	6.67	38.21	69.18	88.69	24.15	48.01	1.017
1,2-NpAc@450 nm-300 K	10.15	17.98	24.11	80.22	162.95	1.79	24.08	1.117
1,2-NpAc@450 nm-340 K	3.28	53.51	8.03	44.74	46.41	1.75	6.16	1.038
1,2-NpAc@450 nm-380 K	1.08	19.60	2.67	21.93	45.59	0.07	2.64	1.001
1,2-NpAc@485 nm-220 K	4.40	40.46	28.25	36.22	164.27	23.33	50.34	1.133
1,2-NpAc@485 nm-260 K	1.70	19.01	56.28	4.98	22.69	72.33	45.11	1.088
1,2-NpAc@485 nm-300 K	9.91	18.44	23.87	80.16	174.64	1.40	23.41	1.072
1,2-NpAc@485 nm-340 K	2.88	47.64	7.36	50.54	37.08	1.81	5.76	0.985
1,2-NpAc@485 nm-380 K	1.02	79.60	2.54	18.94	39.06	1.46	1.86	0.939
1,2-NpAc@620 nm-220 K	0.34	3.55	101.82	96.45	-	-	98.22	0.914
1,2-NpAc@620 nm-260 K	0.25	2.26	90.33	97.73	-	-	88.29	0.910
1,2-NpAc@620 nm-300 K	1.56	1.57	40.60	98.43	-	-	39.98	1.082
1,2-NpAc@620 nm-340 K	0.84	16.96	9.23	83.04	-	-	7.81	1.019
1,2-NpAc@620 nm-380 K	0.45	42.26	2.16	57.74	-	-	1.44	0.928
1,2-NpAc@680 nm-220 K	0.42	2.87	99.05	97.13	-	-	96.22	0.909
1,2-NpAc@680 nm-260 K	0.30	1.42	88.22	98.58	-	-	86.97	1.131
1,2-NpAc@680 nm-300 K	0.22	2.92	39.04	97.08	-	-	37.91	1.008
1,2-NpAc@680 nm-340 K	0.02	66.86	8.26	33.14			2.75	0.947
1,2-NpAc@680 nm-380 K	0.02	21.53	1.54	78.47	-	-	1.21	1.011

(R) 4. ***"Page 6 line 115-117: The authors report "****Accordingly, the photoluminescence quantum yields (PLQYs) largely increased from Ph-X series to 1,2-PhX ones, for instance, the PLQY of Ph-POZ was 9.64%, while that of 1,2-PhPOZ increased to 42.60%"* ***This is not true, while two compounds 1,2-PhPOZ and 1,2-PhAc displayed a large PLQY post annulation, the other three reported compounds showed a weak improvement***

in PLQY, between 3 to 6%. The authors should adapt the discussion. ***"***

We thank the reviewer for this insightful comment, which prompted us to refine our interpretation. Upon closer examination of the data, we recognize that the original statement overgeneralized the annulation effect on PLQY enhancement. Specifically, a substantial increase is observed only for 1,2-PhPOZ and 1,2-PhAc, while the other 1,2-PhX derivatives exhibit modest improvements. The discussion has been revised accordingly to reflect this distinction, ensuring an accurate description of the differential enhancement across the series.

According to the comments, the corresponding discussions have been added to the revised manuscript.

Revised Text in the Manuscript:

"Accordingly, the photoluminescence quantum yields (PLQYs) generally exhibit an increasing tendency from the Ph-X series to the 1,2-PhX ones (Table S3). Notably, pronounced enhancements are observed for 1,2-PhPOZ and 1,2-PhAc, whereas the other derivatives show modest improvements." (Page 6, *line 118-121* in the revised manuscript).

(R) 5. **"*Page 6 line 121-123: The authors wrote:*** *"With the introduction of various solvents with increased polarity from n-hexane to acetonitrile, the maximum emission wavelengths (λ_{em}) of the 1,2 PhX series red-shifted gradually (Figure 2c–2d and Figure S21). Except for 1,2-PhPOZ, the other 1,2-PhX (X = PTZ, Ac, PTZO and PTZOO) showed a two-section linear fitting in low-polarity and high-polarity solvents, indicating the existence of two distinct excited states, according to the Lippert-Mataga solvatochromic model."* ***After reviewing Figure 2d and Figure S21 k)-o) I am not convinced that the presented data show a clear curvature or discontinuity that would necessitate a two-regime fit. In HLCT systems, the coexistence of LE and CT character is typically reflected by a smooth curvature in the solvatochromic plot rather than two imposed linear segments as shown in ref 35 (Adv. Funct. Mater. 2014, 24, 1609–1614). In the case of this article the division into two linear regimes appears to rely on a selective choice of data points, and it is not clear that this segmentation is uniquely justified by the experimental data. For clarity and completeness, the authors need to include a conventional single-linear Lippert–Mataga fit using the full dataset, and to discuss how the conclusions compare with those obtained from the two-section fitting approach and why such a two-section fitting approach is absolutely needed."***

Thanks for your comments. Accordingly, to quantitatively elucidate the nature of the S_1 state, we applied the Lippert–Mataga model to the solvatochromic behavior of the 1,2-PhX series. Solvent polarity parameter

$f(\varepsilon,n)$ was recalculated from static dielectric constants (ε) and refractive indices (n) at 298 K following the treatment described by Reichardt (Solvents and Solvent Effects in Organic Chemistry) (Equation 2). Experimentally, increasing solvent polarity from *n*-hexane to acetonitrile leads to progressive spectral broadening, loss of vibronic structure, and gradual red shifts of emission wavelengths, consistent with a transition from LE-dominated emission in nonpolar solvents to increasing CT character in polar environments. To ensure more continuous coverage of the polarity range and thereby enhance the robustness of the analysis, chlorobenzene was also included as an intermediate-polarity solvent.

$$f(\varepsilon, n) = \frac{\varepsilon - 1}{2\varepsilon + 1} - \frac{n^2 - 1}{2n^2 + 1} \qquad \text{Equation 2}$$

Comparison of single-linear and two-section Lippert–Mataga fits reveals that a single fit across the full solvent range describes the solvatochromic trend with statistical robustness comparable to that obtained from separate fits for low- and high-polarity regimes (Figure R7 and R8). It indicates a continuous evolution of CT character within a single mixed LE/CT state with increasing polarity, rather than a discrete state transition, an interpretation consistent with literature precedents (*J. Phys. Chem. B* 2021, 125, 11717; *Chem. Sci.* 2025, 16, 14478). Quantitative assessment of the excited-state dipole moments for the target annulated luminogens (1,2-PhX series, 1,2-NpAc, and 2,3-NpAc) yielded values ranging from 4.9 to 14.6 D. These relatively small dipole moments are characteristic of LE-dominant HLCT states rather than pure CT states, consistent with the overall solvatochromic behavior described above.

According to the comments, the related discussion has been added to the revised manuscript, and Tables R6-8 were added to the revised supporting information as Table S4, Table S5, and Table S16, while Figure R7 and R8 were added to the revised supporting information as Figure S21 and Figure S46, respectively.

Revised Text in the Manuscript:

"With the introduction of various solvents with increased polarities from n-hexane to acetonitrile, the emission spectra of the 1,2-PhX series undergo gradual red shifts (10-20 nm) and progressive broadening, evolving from well-resolved vibronic structures to featureless bands (Figure 2c–d and Figure S21). The excited-state dipole moments derived from quantitative analysis, ranging from 4.9 to 14.6 D, confirm the involvement of partial CT character in the excited state (Table S4 and Table S5)." (Page 6, *line 122-127* in the revised manuscript).

"Increasing solvent polarity (from n-hexane to acetonitrile) resulted in gradual red shifts of 12 nm (1,2-NpAc) and 16 nm (2,3-NpAc), respectively, along with progressive spectral broadening in their PL spectra. These spectra evolved from well-resolved vibronic structures to featureless bands, closely resembling the

behavior observed for 1,2-PhAc. The derived excited-state dipole moments, ranging from 8.0 D to 9.9 D for 1,2-NpAc and from 6.8 D to 11.8 D for 2,3-NpAc, further confirm the partial CT character of these excited states (Figure 3c, Figure S46, and Table S16).” (Page 10, *line 209-212*, and Page 11, *line 213-214* in the revised manuscript).

Table R6. Calculation of f(ε, n) in various solvents

Solvent	ε	n	f(ε, n)
Cyclohexane	1.88	1.375	-0.002
n-hexane	2.02	1.426	-0.001
Toluene	2.38	1.497	0.013
Chlorobenzene	5.62	1.524	0.143
Chloroform	6.02	1.372	0.150
Ethyl acetate	8.93	1.424	0.199
THF	4.89	1.446	0.210
Dichloromethane	7.58	1.407	0.217
DMF	36.71	1.430	0.274
Acetone	20.56	1.359	0.284
Acetonitrile	35.94	1.344	0.304

Table R7. Solvatochromic effect investigation

Solvent	f(ε, n)	ν_a-ν_f (cm^{-1})				
		1,2-PhPOZ	1,2-PhPTZ	1,2-PhAc	1,2-PhPTZO	1,2-PhPTZOO
Cyclohexane	-0.002	1637.722	3285.129	1894.284	851.3273	968.773
n-hexane	-0.001	1646.316	3362.936	1823.362	851.3273	890.529
Toluene	0.013	1809.651	3464.909	1954.064	1057.027	1174.168
Chlorobenzene	0.143	2107.426	3552.849	2059.354	1129.484	1235.178
Chloroform	0.150	2262.82	3739.36	2367.424	1280.236	1471.158
Ethyl acetate	0.199	2236.257	3661.116	2162.372	1294.41	1397.516
THF	0.210	2335.669	3836.575	2448.362	1431.046	1904.762
Dichloromethane	0.217	2274.552	3620.961	2299.43	1351.92	1471.158
DMF	0.274	2738.152	4045.629	2702.15	1502.347	2046.23
Acetone	0.284	2738.152	4009.516	2716.833	2127.347	2128.096
Acetonitrile	0.304	2798.079	4188.199	2783.088	2012.822	2210.433
a (Å)		4.34	4.24	4.43	4.30	4.35
$\Delta\mu = (\mu_e - \mu_g)^a$		6.7 D	5.4 D	6.5 D	6.5 D	7.1 D
$\Delta\mu = (\mu_e - \mu_g)^b$		-	3.6 / 6.4 D	4.3 / 7.7 D	4.6 / 8.2 D	4.8 / 8.7 D
μ_g		1.2 D	1.3 D	1.0 D	4.6 D	5.9 D
μ_e^a		7.9 D	6.7 D	7.5 D	11.1 D	13.0 D
μ_e^b		-	4.9 / 7.7 D	5.3 / 8.7 D	9.2 / 12.8 D	10.7 / 14.6 D

[a] Using single-linear fitting; [b] Using two-section linear fitting.

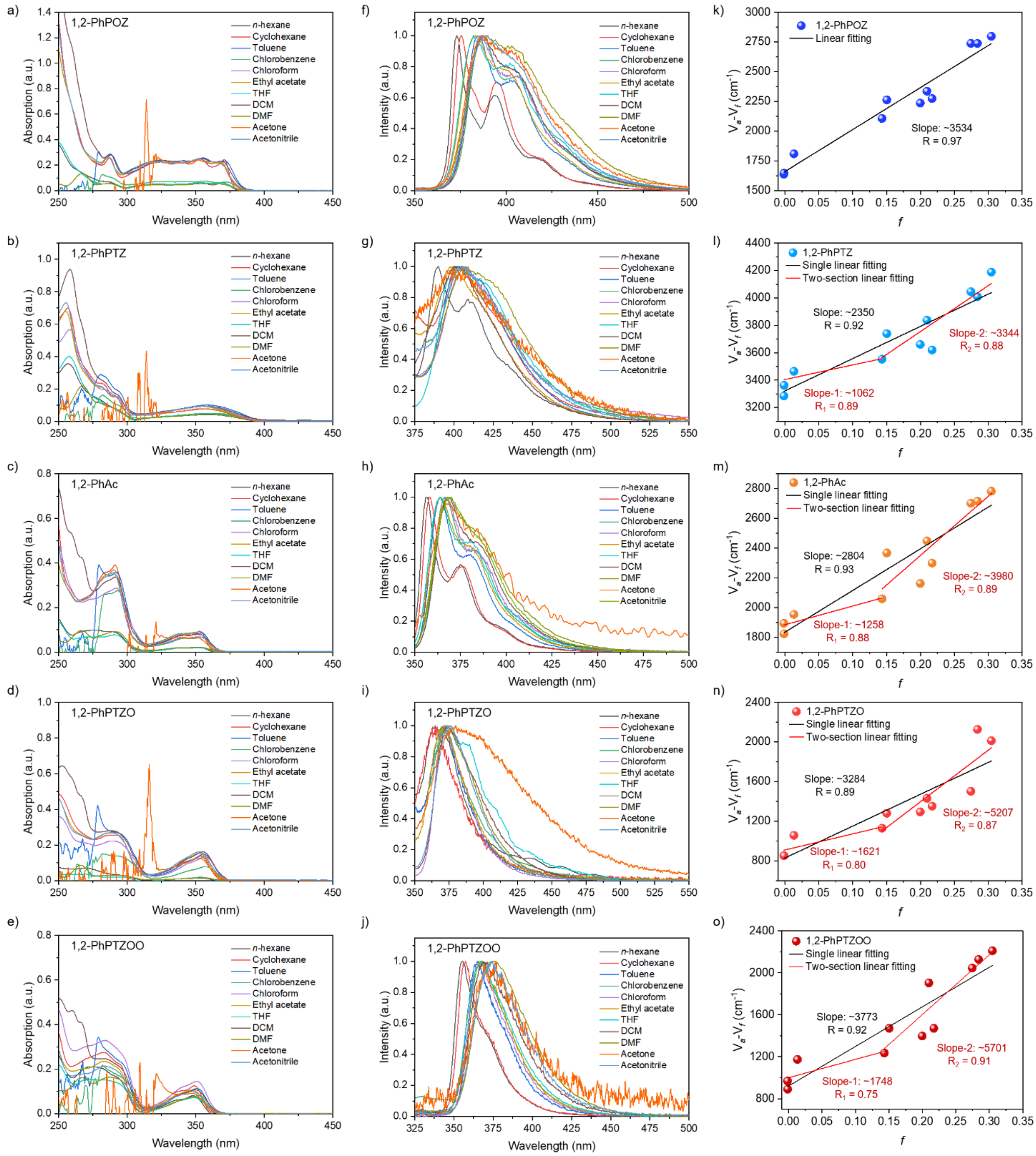

Figure R7. a-e) UV-Vis absorption and f-j) PL spectra of 1,2-PhPOZ, 1,2-PhPTZ, 1,2-PhAc, 1,2-PhPTZO and 1,2-PhPTZOO (10 μM) in different solutions at room temperature. k-o) The solvatochromic Lippert－Mataga models of 1,2-PhPOZ, 1,2-PhPTZ, 1,2-PhAc, 1,2-PhPTZO and 1,2-PhPTZOO (V_a-V_f = $(1/\lambda_{abs} - 1/\lambda_{PL}) * 10^7$).

Table R8. Solvatochromic effect investigation of 1,2-NpAc and 2,3-NpAc

Solvent	$f(\varepsilon, n)$	ν_a-ν_f (cm^{-1})	
		1,2-NpAc	2,3-NpAc
Cyclohexane	-0.002	1841.268	3285.129
n-hexane	-0.001	1775.013	3362.936
Toluene	0.013	1897.08	3464.909
Chlorobenzene	0.143	2080.804	3502.116
Chloroform	0.150	2346.890	3739.36
Ethyl acetate	0.199	2091.817	3661.116
THF	0.210	2359.292	3836.575
Dichloromethane	0.217	2283.611	3620.961
DMF	0.274	2485.553	4045.629
Acetone	0.284	2561.653	4009.516
Acetonitrile	0.304	2700.831	4188.199
a (Å)		6.03	5.92
$\Delta\mu = (\mu_e - \mu_g)^a$		8.3 D	8.8 D
$\Delta\mu = (\mu_e - \mu_g)^b$		7.1 / 9.0 D	5.8 / 10.8 D
μ_g		0.9 D	1.0 D
μ_e^a		9.2 D	9.8 D
μ_e^b		8.0 / 9.9 D	6.8 / 11.8 D

[a] Using single-linear fitting; [b] Using two-section linear fitting

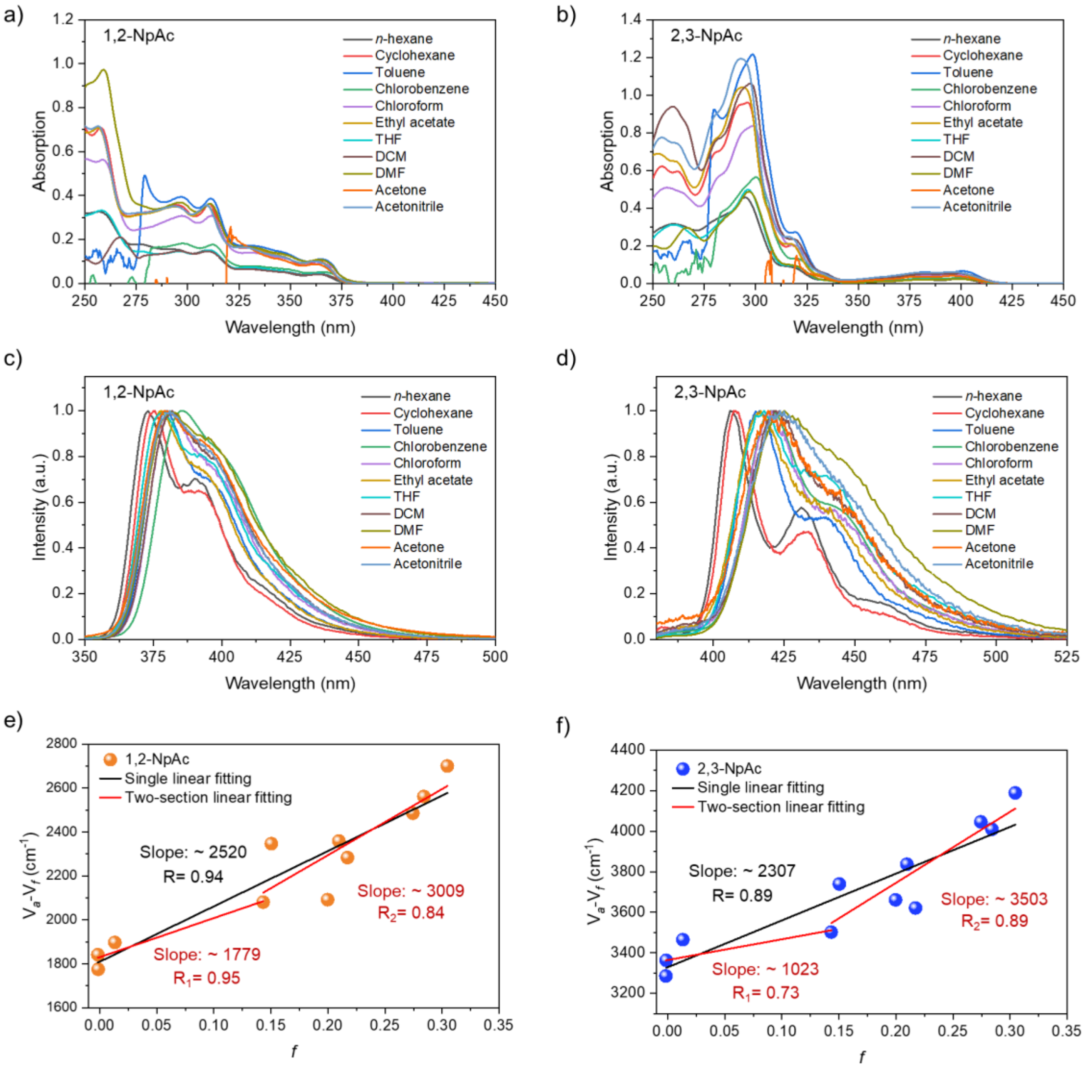

Figure R8. a-b) UV-Vis absorption and c-d) PL spectra of 1,2-NpAc and 2,3-NpAc (10 μM) in different solutions at room temperature. e-f) The solvatochromic Lippert–Mataga models of 1,2-NpAc and 2,3-NpAc (V_a-V_f = $(1/\lambda_{abs} - 1/\lambda_{PL}) * 10^7$).

(R) 6. ***"Page 9 line 180-185. 1. The authors assignment of the pronounced temperature-dependent phosphorescence color shift to emission from higher-lying triplet states (T_n) is intriguing but, in its current form, not fully substantiated. In organic crystals, large spectral shifts upon cooling can also arise from reduced triplet exciton migration and suppression of defect- or trap-mediated emission, which is typically red-shifted. In addition, temperature-induced changes in exciton localization or subtle modifications of intermolecular packing may contribute without requiring a change in the emitting electronic state as shown in the following paper: https://www.sciencedirect.com/science/article/pii/S0022231320316987. where blue shifted and enhanced phosphorescence from crystalline powder was observed upon temperature decrease.***

More comprehensive temperature-dependent lifetime analysis at different temperatures not just at room temperature as shown in Fig. S40, would be valuable to highlight in more detail the mechanism.”

Thanks for your comments. We agree that the possible mechanisms (e.g., suppressed triplet exciton migration, trap-mediated emission, and temperature-induced packing modifications) must be carefully ruled out, and that more comprehensive temperature-dependent lifetime data are essential. A detailed response to each point is provided below.

(i) Ruling Out Triplet Exciton Migration and Trap-Mediated Emission

Although phenomena such as suppressed triplet exciton migration, trap-mediated emission, or enhanced exciton localization can lead to spectral shifts at low temperatures, such continuous shifts are not observed in our system. Critically, the wavelengths of both short- and long-wavelength delayed emission components remain constant throughout the temperature range studied. The observed color change is therefore attributable not to spectral migration, but to a temperature-dependent repartitioning of emission intensity between these two distinct and stationary bands. Upon heating from 220 K to 340 K, the short-wavelength delayed emission (450 nm and 485 nm) exhibits a pronounced intensity increase, which slightly diminishes above 340 K up to 380 K. Meanwhile, the long-wavelength delayed emission (620 nm and 680 nm) undergoes a monotonic intensity decrease with rising temperature. This opposing temperature dependence results in a progressive afterglow color shift from red at 220 K to blue at 300 K, driven solely by intensity redistribution between the two fixed emission bands.

Importantly, trap-mediated emission typically manifests as broadened, structureless, and red-shifted bands, often accompanied by peak shifts or spectral narrowing upon cooling. None of these features is observed in our system. Instead, the persistence of multiple well-defined delayed emission bands with invariant peak positions provides compelling evidence for the coexistence of several intrinsic emissive states originating from triplet configurations.

(ii) Ruling Out Temperature-Induced Exciton Localization and Packing Modifications

Changes in molecular packing can be ruled out based on temperature-dependent crystal structural analysis of 1,2-NpAc. From 220 K to 380 K, the unit-cell volume expands by only 2.4%, with intermolecular distances varying by ~0.1 Å—changes comparable to or smaller than those in the analogous compounds 1,2-PhAc (2.5%) and Ph-Ac (3.3%). Yet, the emission shift in 1,2-NpAc (170 nm) far exceeds those of the analogous compounds (45 nm and 15 nm, respectively). This lack of correlation between structural and spectral changes indicates that thermal expansion or packing modifications are not the primary cause. Instead, the observed behavior arises

from temperature-dependent excited-state population redistribution and relaxation dynamics.

(iii) Comprehensive Temperature-Dependent Lifetime Analysis

Temperature-dependent lifetime measurements provide strong evidence for the distinct mechanisms underlying the short- and long-wavelength delayed emissions in 1,2-NpAc (Figure R9). The data show that as the temperature rises from 220 K to 380 K, the long-wavelength emission (620 nm and 680 nm) undergoes severe quenching, with its lifetime plummeting from 98.22 ms and 96.22 ms to just 1.44 ms and 1.21 ms. This behavior is characteristic of phosphorescence, which is highly susceptible to thermally activated nonradiative decays. In contrast, the short-wavelength afterglow (450 nm and 480 nm) exhibits weaker temperature dependence, with lifetimes decreasing modestly from 41.04 ms and 50.34 ms to 2.64 ms and 1.86 ms, respectively. This behavior is consistent with a T_n-mediated up-conversion pathway ($T_n \rightarrow S_1 \rightarrow S_0$), where thermally activated RISC competes with nonradiative decay, partially offsetting the expected lifetime shortening.

According to the comments, the corresponding discussions have been added to the revised manuscript, and Figure R9 was added to the revised supporting information as Figure S54.

Revised Text in the Manuscript:

"*Thus, emission modulation due to temperature-induced changes in molecular packing can be discounted, as the crystal structure exhibits only negligible variations across the studied temperature range.*" (Page 17, *line 355-356* in the revised manuscript)

"*Additionally, the possibility of defect-related emission was excluded by the similar excitation spectra obtained for emissions at 450 nm, 485 nm, 620 nm, and 680 nm (Figure S60). Collectively, these spectroscopic tests, together with the analyses of temperature-dependent exciton dynamics, crystal structures, and theoretical calculations, provide strong support for T_n-mediated DF and phosphorescence mechanism.*" (Page 18, *line 361-365* in the revised manuscript)

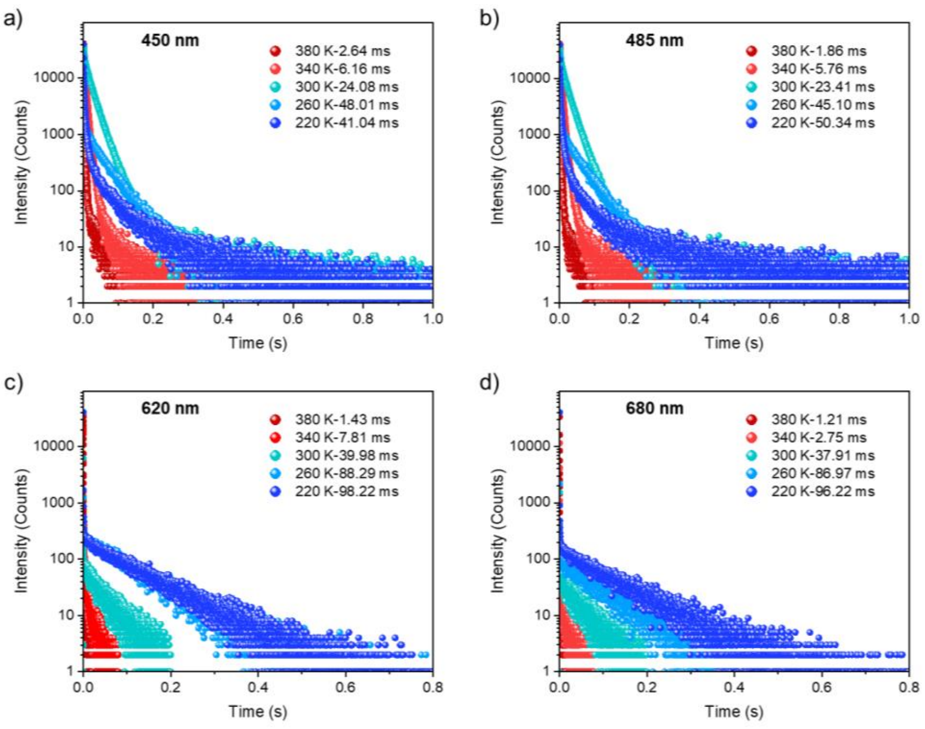

Figure R9. Temperature-dependent delayed emission decay profiles of 1,2-NpAc crystals monitored at a) 450 nm, b) 485 nm, c) 620 nm, and d) 680 nm over the temperature range of 220 K – 380 K.

(R) 7. ***Additionally, the authors mentioned Figure S41 in the following sentence: ".[…] the exponential decay observed in time-resolved emission spectroscopy (TRES) measurements (Figure S40 and S41)" which makes it look like Figure S41 focuses on TRES measurements which is not the case. The fact is the content of Figure S41 is not clearly discussed by the authors in the manuscript.***

We thank the reviewer for identifying the inappropriate placement of Figure S41. We concur that its original citation alongside the TRES measurements was misleading. To rectify this, we have removed Figure S41 from the relevant sentence in the revised manuscript. It has now been moved to the section on excited-state energy-level analysis and renumbered as Figure S43.

(R) 8. ***Page 12 line 237-239 the Authors reported: "the afterglow emission at short wavelengths (450 nm and 485 nm) initially showed exponential decay; subsequently, linear components appeared with a linear slope close to 1, indicating the existence of phosphorescence and TADF emission."***

Thanks for your comments. Accordingly, we address the reviewer's comments point by point:

A few points are unclear about this sentence.

1. Why is the plot reported Figure 3h is in log vs log? This is an uncommon way to report decays usually

shown is log (photon count) vs linear time (as explain above).

We thank the reviewer for pointing out the use of a log - log plot in Figure 3h. We agree that a semi-logarithmic representation is the standard format for displaying photoluminescence decays and determining lifetimes. However, it is important to note that the data shown in Figure 3h are fundamentally different from a conventional decay curve. The plot is constructed from time-resolved emission spectra (TRES), with each point corresponding to the integrated intensity at a given wavelength and delay time. This methodology grants us access to excited-state relaxation processes spanning an unusually wide temporal range, from microseconds to seconds. The log - log format was chosen specifically because it allows for the simultaneous visualization of distinct dynamical regimes (e.g., prompt fluorescence, delayed fluorescence, and phosphorescence) that would be difficult to capture clearly on a semi-log plot. This approach is well-established in the field, as evidenced by recent publications (*Adv. Opt. Mater.* 2022, 10, 2101784; *J. Am. Chem. Soc.* 2023, 145, 25495).

2. ***Why is the ordinate axis of the plot of Figure 3h described as Intensity (a.u.)? is it not photon counts like the previous decays reported?***

We thank the reviewer for pointing out this inconsistency. In the revised manuscript and Supporting Information, the ordinate label has been corrected to "Intensity (counts)" to ensure consistency with the experimental method.

3. ***In the case the ordinate is related to photon count and not an arbitrary intensity, the linear plot appears at extremely low counts (below 10). The late-time decay component, which appears approximately linear on a log–log plot, emerges at photon count levels below ~10, where Poisson noise and background contributions are expected to dominate. In this regime, extracting decay slopes is statistically unreliable, and a linear dependence on a log–log scale merely reflects apparent power-law behavior (linear on log/log plot) rather than a specific emissive process. Consequently, the assignment of this component to phosphorescence and TADF emission is not sufficiently supported. Moreover, usual TADF and phosphorescence should be shown and analyzed on log/linear plots.***

We appreciate the reviewer's constructive comment concerning the potential impact of noise in the low-photon-count regime. We concur that as the signal approaches the background level, extracting reliable quantitative information from the decay dynamics is no longer feasible.

To address this concern, we have carefully re-examined our data. As shown in Figure R5, all background signals have been rigorously subtracted and are consistently well below the reported delayed

emission intensities, ensuring that the extracted decay profiles are not compromised by noise. Moreover, we emphasize that the assignment of DF and phosphorescence does not rely solely on the log–log representation in Figure 3h. Complementary analyses provide robust support: (i) millisecond-scale lifetimes were independently extracted from conventional semi-logarithmic decay fitting, and (ii) the temperature-dependent emission behaviors are fully consistent with the proposed mechanisms. Together, these converging lines of evidence firmly establish the assignment of DF and phosphorescence in this system.

(R) 9. ***"Page 13 line 264-268: The temperature-dependent enhancement of the ESA feature around 780 nm is intriguing and suggests thermally activated excited-state dynamics. However, attributing this behavior directly to a hot reverse intersystem crossing (hRISC) process may be premature. More discussion is necessary."***

Thanks for your comments. We fully agree that attributing the temperature-dependent enhancement of the ~780 nm ESA feature directly to *h*RISC process requires caution and more detailed discussions. We wish to clarify that our original manuscript did not claim this ESA enhancement as definitive proof of *h*RISC; rather, it was presented as an indication of more complex thermally activated dynamics.

To substantiate this point and provide deeper mechanistic insight, we have conducted nanosecond time-resolved transient absorption measurements with higher temporal resolution (Figure R10a). The results reveal a sequential spectral evolution on the nanosecond timescale. Immediately after photoexcitation, ESA bands centered at ~450 nm and ~600 nm rise promptly. Over the subsequent 1.5–2.5 ns window, the 450 nm feature gradually decays, while the 600 nm band and a broad ESA feature spanning 700–800 nm continue to grow before eventually decaying at longer delays. This clear population redistribution demonstrates that the excited-state relaxation involves multiple competing channels rather than a single decay pathway.

Integrating these transient absorption dynamics with temperature-dependent emission spectra (Figure R10b–c) reveals a consistent mechanistic picture. As temperature increases, the short-wavelength delayed emission (450 nm and 485 nm) intensifies markedly, with only a marginal decrease at 380 K, whereas the long-wavelength phosphorescence (620 nm and 680 nm) diminishes progressively across the entire temperature range. This opposing thermal behavior reflects a temperature-modulated competition between distinct relaxation channels, specifically, a shift in population from the $T_n \rightarrow T_1 \rightarrow S_0$ phosphorescence pathway toward $T_n \rightarrow S_1$

→ S_0 delayed fluorescence *via* thermally activated up-conversion.

Collectively, these observations provide robust support for the involvement of thermally activated processes originating from higher-lying triplet states, without overinterpreting the ESA data in isolation.

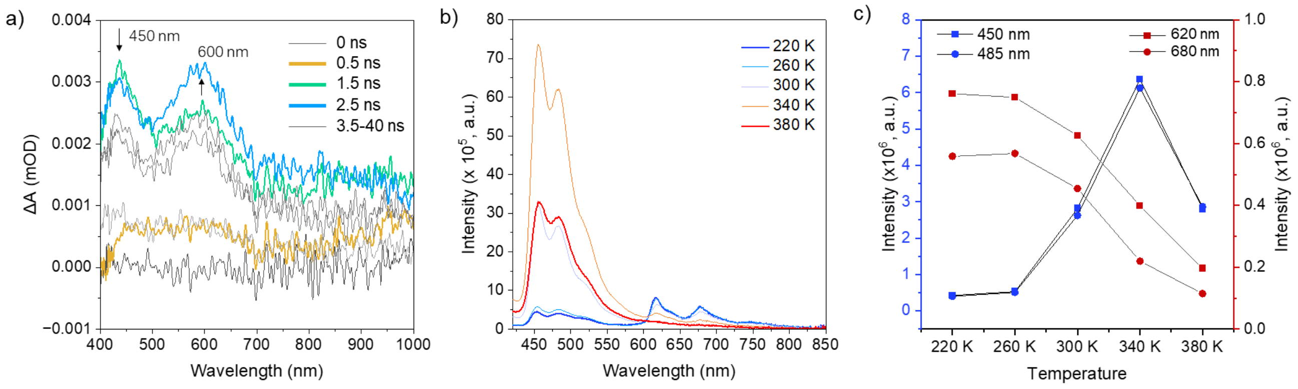

Figure R10. a) Nanosecond-TA spectroscopy of 1,2-NpAc crystal. b) Delayed emission spectra (1 ms delay) of 1,2-NpAc crystals at different temperatures from 220 K to 380 K. c) Temperature-dependent intensity evolution of the delayed emission monitored at 450 nm, 485 nm, 620 nm, and 680 nm over the range of 220 K – 380 K.

According to the comments, the corresponding discussions have been added to the revised manuscript, and Table R10a was added to the supporting information as Figure S57.

Revised Text in the Manuscript:

"*Femtosecond and nanosecond time-resolved transient absorption (TA) spectroscopy were employed to investigate the excited-state dynamics of the 1,2-NpAc crystal across multiple timescales. Following photoexcitation (λ_{ex} = 365 nm) at 220 K and 298 K, two primary excited-state absorption (ESA) bands are observed at ~600–700 nm and ~800–900 nm (Figures 4d and 4e). Notably, at 298 K, the ESA peak around 780 nm became significantly more pronounced within 0.1–0.66 ps, indicating that temperature affects the excited-state processes and promotes additional population redistribution processes. This complexity is further underscored by nanosecond TA measurements (Figure S57), which track the subsequent population evolution. The data reveal a clear spectral cascade: the ESA features at approximately 450 nm and 600 nm emerge promptly, followed by the rise of a broad 700–800 nm band within 1.5–2.5 ns. This sequential spectral evolution spanning the ultrafast to nanosecond timescale indicates that the excited-state relaxation proceeds through multiple pathways, accompanied by population redistribution among different excited states. Such a multichannel excited-state landscape provides the possibility for triplet excitons to undergo both RISC to S_1 state to generate DF and radiative decay to the ground state to produce phosphorescence.*" (Page 16, *line 320-*

333 in the revised manuscript)

Minor

1. ***Figure 1 page 4: The definition of TADF vs hot-exciton as described by Y. Ma in his review: Chem. Soc. Rev., 2021,50, 1030-1069 states than TADF mainly involves S_1 and T_1 excited state in the ISC and RISC process. Ma and colleagues explain that involvement of higher energy excited state such as S_n and or T_n leads to the phenomenon of hot exciton. Can the authors share their opinion of choosing the term TADF in Figure 1b and c as these show T_n involved processes?***

We thank the reviewer for bringing this important terminological distinction to our attention. We have carefully considered the definition of TADF *versus* hot-exciton mechanisms as outlined in the insightful review by Prof. Ma and colleagues (*Chem. Soc. Rev.*, 2021, 50, 1030). We agree that, in a strict sense, TADF primarily involves the lowest singlet (S_1) and triplet (T_1) states, whereas processes involving higher-lying excited states (S_n or T_n) are more accurately described within the "hot-exciton" framework.

In light of this, we acknowledge that the processes illustrated in our original Figure 1b and 1c involve T_n-mediated pathways and therefore should not be labeled as TADF. To avoid ambiguity and adhere to a more rigorous terminology, we have revised Figure 1 accordingly, replacing "TADF" with the more general term "DF" (delayed fluorescence). We believe this correction aligns our manuscript with the precise definitions in the field.

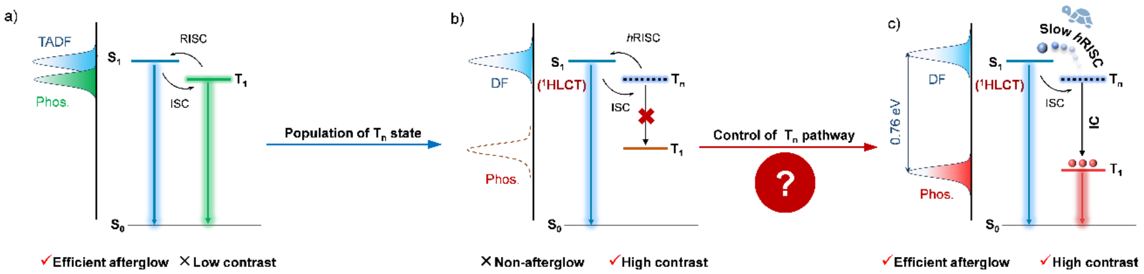

Figure R11. a) Schematic illustration of phosphorescence and TADF emission related to the transitions from T_1 states. b) Schematic illustration of DF (delayed fluorescence) emission from high-level triplet states (T_n) by *h*RISC processes. c) Schematic illustration of both phosphorescence and DF emission related to the transitions from T_n states.

2. ***Figure 1d: 1. In this figure the authors decided to show TADF-Phosphorescence emission gap vs PLQY. The issue is that the TADF-Phosphorescence gap has been reported in units of nanometers which is not***

suitable to report energy gaps as wavelength is not linearly proportional to energy. Such energy differences relevant to thermally activated processes should be reported in energy units (eV or cm^{-1}) to allow meaningful physical interpretation and comparison.

Thank you for your comments. We agree that reporting energy differences, particularly those relevant to thermally activated processes, in energy units (such as eV or cm^{-1}) is essential for accurate physical interpretation and meaningful comparison. In response, we have revised Figure 1d by converting the DF-phosphorescence emission gap from nanometers to the appropriate energy units. This modification ensures that the data are presented in a manner that reflects their physical significance.

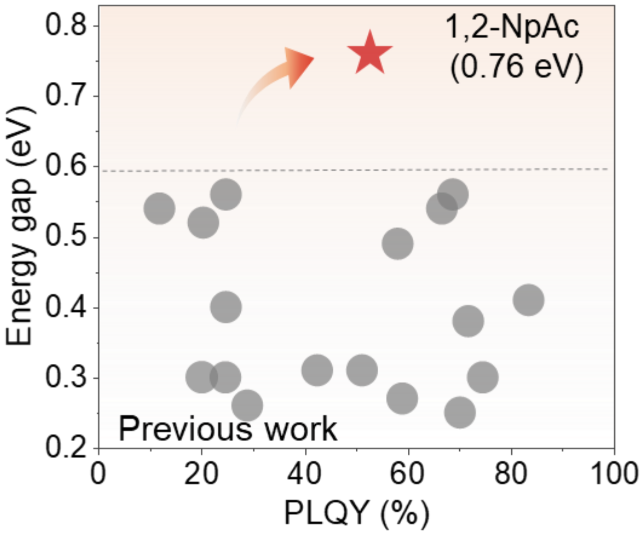

Figure R12. the recent progress in temperature-modulated afterglow (observable by the naked eyes) through the conversion between DF and phosphorescence in single-component systems (y-axis corresponds to the energy gap between DF and phosphorescence, and x-axis corresponds to the PLQY)

3. ***At the end of the manuscript such as page 13 line 244 the authors again use nanometer to highlight a wavelength shift which would be more appropriately underlined in a unit linearly proportional to energy. Throughout the paper all discussions of these values should be change to eV.***

We are grateful to the reviewer for this valuable comment, which highlights an important aspect of data presentation. The reviewer correctly notes that for discussing thermally activated processes, reporting data in energy units (eV) is essential for accurate physical interpretation, as it is linearly proportional to the electronic transition energy. We fully agree with this principle. At the same time, we considered the standard conventions in our field of optical spectroscopy, where spectra are typically recorded and presented in wavelength (nm) to maintain a direct link to the raw experimental data. To reconcile these two valid

perspectives, we have adopted a balanced approach in the revised manuscript. While we have kept the primary presentation of spectral features in nanometers for fidelity to the experimental measurements, we now explicitly provide the corresponding photon energies in electronvolts (eV) in parentheses wherever energetic shifts are discussed. This dual presentation ensures both experimental accessibility and physical relevance.

According to the comments, the corresponding discussions have been added to the revised manuscript.

Revised Text in the Manuscript:

"*Accordingly, the dominated emission peaks exhibit a temperature-dependent shift of up to 170 nm, from 450 nm at 298 K to 620 nm at 220 K, corresponding to an energy gap of 0.76 eV. This represents the largest spectral separation between DF and phosphorescence reported to date in temperature-modulated afterglow materials.*" (Page 14, *line 280-283* in the revised manuscript)

4. ***Page 6 line 110 – Figure S17a: The author chose to show the absorption spectra of compounds in THF. THF absorption cut-off happens at 215 nm, which means that data below the cut-off are not particularly relevant.***

Thanks for your comment. Accordingly, the absorption spectra of the compounds in THF have been truncated below 215 nm and updated in the revised Supporting Information.

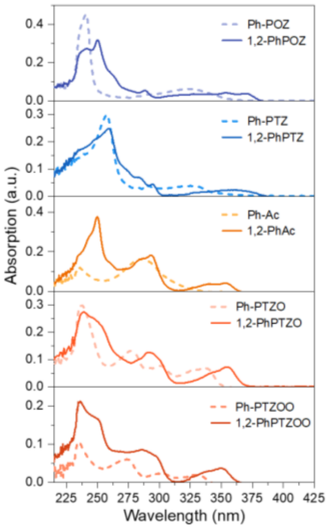

Figure R13. The UV-vis absorption spectra of Ph-X and 1,2-PhX (X = POZ, PTZ, Ac, PTZO and PTZOO) in THF (10 μM) at room temperature.

5. ***Page 8 Line 150-151: Two typos are present: "3.29s@425nm and 5.76s@420nm" should be written as "3.29s at 425nm" and "5.76s at 420nm"***

Thanks for your comment. Accordingly, the *"3.29s@425nm and 5.76s@420nm"* have been revised as *"3.29 s at 425 nm"* and *"5.76 s at 420 nm"*, respectively.

6. ***Page 5 line 98: The phrase "proportions of (n, π) and (π,π*) characterization" is somewhat imprecise and need rephrasing such as: "The introduction of different heteroatoms alters the orbital composition of the excited states, shifting their n→π versus π→π* character and consequently modulating intersystem crossing and radiative decay pathways."***

Thanks for your comment. Accordingly, the phrase "proportions of (n, π) and (π, π*) characterization" has been revised to "The introduction of different heteroatoms alters the orbital composition of the excited states, shifting their n→π* *versus* π→π* character and consequently modulating intersystem crossing and radiative decay pathways" in the revised manuscript (Page 5, *line 100-102*).

7. ***Page 5 line 100: C-N coupling reaction is more commonly known as "Buchwald-Hartwig C-N coupling"***

Thanks for your comment. Accordingly, the "C-N coupling reaction" has been revised to "Buchwald-Hartwig C-N coupling" in the revised manuscript (Page 5, *line 102-103*).

8. ***Figure 2 i): The authors show the evolution of the delayed emission of 1,2-PhAc as a function of temperature. In addition to the figure shown, it would be appreciable to visualize this evolution with normalized spectra even in a 1D plot in the SI.***

Thanks for your comment. Accordingly, the normalized temperature-dependent delayed emission spectra of 1,2-PhAc crystal (Figure R14) are now provided as a 1D plot in the revised Supporting Information (Figure S40) for improved visualization.

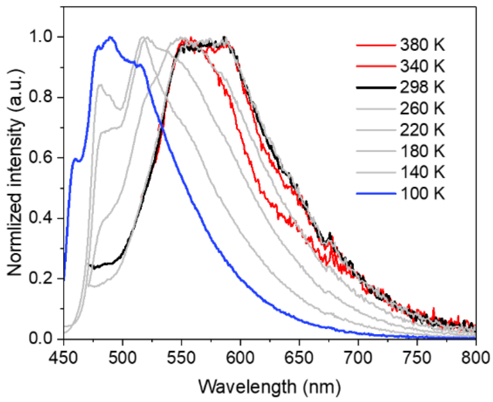

Figure R14. Normalized temperature-dependent delayed emission spectra of 1,2-PhAc crystal across 100 K-380 K.

ESI:

1. **NMRs:**

"All numerical data for carbon NMR reported include the 3 peaks of the solvent: deuterated chloroform. The authors should not include these peaks when reporting the numerical NMR data. Additionally, for molecule 1,2NpAc the signal of the quaternary carbon at 32.17 ppm visible on the spectra reported below, is missing from the description of carbon peaks. It should be added."

Thanks for your comment. Accordingly, the ^{13}C NMR data for all target compounds have been corrected in the revised Supporting Information by removing the solvent peaks (deuterated chloroform) from the reported listings. Additionally, for molecule 1,2NpAc, the missing quaternary carbon signal at 32.17 ppm has been added to the description of carbon peaks.

Compound 1,2-PhPOZ. ^{13}C NMR (100 MHz, $CDCl_3$) δ (ppm): 145.58, 142.71, 136.65, 128.98, 128.85, 126.53, 125.12, 124.24, 123.99, 123.01, 122.93, 121.42, 120.58, 117.55, 114.33, 113.89, 112.21, 108.59.

Compound 1,2-PhPTZ. ^{13}C NMR (100 MHz, $CDCl_3$) δ (ppm): 138.62, 138.24, 136.93, 128.21, 127.59, 126.38, 126.04, 124.37, 123.76, 122.77, 121.89, 121.51, 121.14, 120.89, 117.44, 117.06, 115.33, 114.41.

Compound 1,2-PhAc. ^{13}C NMR (100 MHz, $CDCl_3$) δ (ppm): 138.20, 136.06, 135.95, 134.36, 129.44, 127.26, 127.06, 126.27, 126.18, 123.20, 122.35, 121.90, 121.55, 121.01, 120.74, 117.40, 113.92, 113.62, 36.77, 32.53.

Compound 1,2-PhPTZO. ^{13}C NMR (100 MHz, $CDCl_3$) δ (ppm): 138.82, 135.07, 134.01, 133.31, 133.24, 127.73, 127.46, 126.10, 125.18, 124.38, 124.04, 123.62, 123.09, 121.52, 115.83, 114.84.

Compound 1,2-PhPTZOO. ^{13}C NMR (100 MHz, $CDCl_3$) δ (ppm): 138.85, 136.33, 135.45, 133.34, 128.09, 127.16, 125.67, 125.31, 124.36, 124.31, 123.51, 123.43, 121.68, 120.27, 115.67, 114.76.

Compound 1,2-NpAc. ^{13}C NMR (100 MHz, $CDCl_3$) δ (ppm): 140.35, 137.55, 135.61, 135.07, 133.41, 130.99, 129.15, 126.12, 125.27, 125.02, 124.97, 124.82, 124.62, 123.96, 123.77, 123.70, 123.23, 123.18, 119.79, 119.41, 117.42, 116.76, 37.93, 32.17.

Compound 2,3-NpAc. ^{13}C NMR (100 MHz, $CDCl_3$) δ (ppm): 129.12, 128.73, 128.14, 127.87, 127.57, 127.20, 127.20, 125.55, 123.90, 123.11, 122.93, 122.48, 121.84, 119.18, 118.07, 113.69, 109.67, 109.59, 36.74, 32.42.

2. Synthesis

"The synthetic route of 1,2-PhPTZO and 1,2-PhPTZOO. I assume that the authors obtain a mixture of 1,2-PhPTZO and 1,2-PhPTZOO at the end of the procedure. As such I would appreciate if the author could add the Rf values for both compounds along with order of elution. Adding Rf and elution order for other compounds would be nice but not mandatory as in this case where, to my understanding, both are separated from the same reaction mixture."

Thank you for your comment. Accordingly, we have revised the synthetic procedure in the Supporting

Information to address this point. The updated version now specifies the elution order and provides the Rf values for both 1,2-PhPTZO and 1,2-PhPTZOO, offering a clearer explanation of their separation from the same reaction mixture.

The procedure has been updated as follows:

"*1,2-PhPTZ (1.0 mmol, 273.4 mg) was dissolved in dichloromethane (10 mL), acetic acid (5 mL), and H_2O_2 (0.5 mL), and the mixture was stirred at 60 °C for 8 hours. The reaction mixture was then extracted with dichloromethane and purified by column chromatography. Elution with dichloromethane/petroleum ether (2:1, v/v) first afforded 1,2-PhPTZOO ($R_f \approx 0.5$). Increasing the eluent polarity to dichloromethane/ethyl acetate (10:1, v/v) subsequently yielded 1,2-PhPTZO ($R_f \approx 0.3$).*"

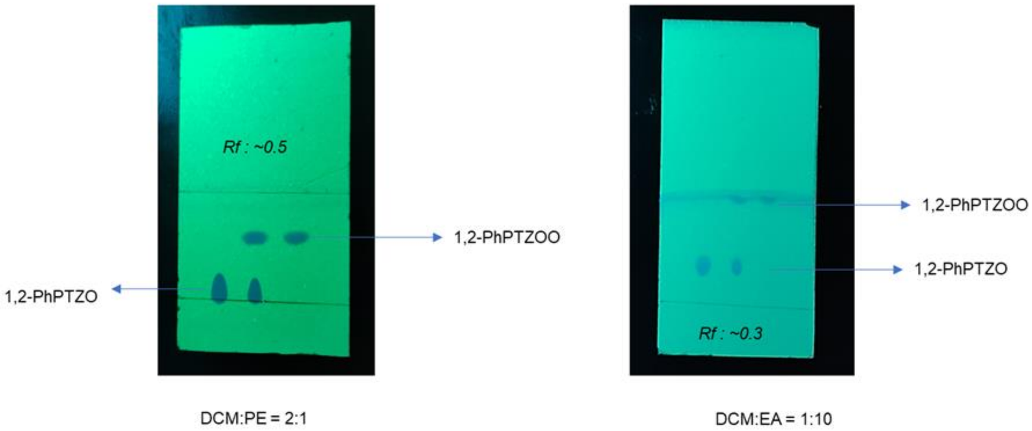

Figure R15. Thin-layer chromatography (TLC) analysis of the 1,2-PhPTZO and 1,2-PhPTZOO mixture.

The synthetic route of 1,2-NpAc and 2,3-NpAc

"The crude product was purified by column chromatography on silica gel using petroleum ether and dichloromethane as eluents." I would appreciate if the authors could describe exactly the proportion of each solvent depending on the molecule or if they were used as gradients. Can be added to the characterization part.

Thanks for your comment. Accordingly, we have incorporated the exact proportions of petroleum ether and dichloromethane into the synthetic procedures for 1,2-NpAc and 2,3-NpAc. The revised procedures now read as follows:

For 1,2-NpAc:

9,9-Dimethyl-9,10-dihydroacridine (3.0 mmol, 1.5 equiv.), 1-bromo-2-iodonaphthalene (2.0 mmol, 1.0 equiv.), potassium *tert*-butoxide (6.0 mmol, 3.0 equiv.) and trans-dichlorobis(tricyclohexylphosphine)palladium(II) (0.2

mmol, 0.1 equiv.) were dissolved in toluene (10 mL) in a Schlenk tube. The resultant mixture was refluxed at 120 °C for 12 hours under nitrogen atmosphere, then extracted with dichloromethane. The combined organic extracts were dried over anhydrous Na_2SO_4 and concentrated by rotary evaporation. The crude product was purified by column chromatography on silica gel using petroleum ether/dichloromethane (**100:1**, v/v) as the eluent.

For 2,3-NpAc:

A similar procedure was followed using 2,3-dibromonaphthalene (2.0 mmol, 1.0 equiv.) in place of 1-bromo-2-iodonaphthalene. The crude product was purified by column chromatography on silica gel using petroleum ether/dichloromethane (**100:1**, v/v) as the eluent.

Note on data processing revisions.

In the revised manuscript, two data-processing procedures have been updated to ensure methodological consistency and facilitate comparison with the literature.

First, the Lippert–Mataga analysis was revisited using a consistent and well-established set of solvent polarity parameters $f(\varepsilon,n)$, calculated from the static dielectric constants (ε) and refractive indices (n) at 298 K following the treatment described in Solvents and Solvent Effects in Organic Chemistry by Christian Reichardt.

Second, the method used to evaluate the energy gap between the DF and phosphorescence emissions has been revised, according to the reviewer's suggestion. In the original analysis, the energy difference was estimated from the spectral onsets of the DF and phosphorescence emissions. However, the onset positions cannot be reliably extracted from the literature spectra used for comparison. Therefore, to ensure a consistent evaluation protocol, the energy gap was recalculated using the difference between the emission peaks of the DF band and the phosphorescence band. This peak-based approach has also been adopted in previous reports (*Angew. Chem. Int. Ed.* 2025, 64, e202423602). As a result, the evaluated energy gap was adjusted from 0.99 eV to 0.76 eV (from ΔE = 1240/450-1240/620), and the corresponding wavelength difference changed from 195 nm to 170 nm. The relevant values in the manuscript have been updated accordingly.

Table R9 Solvatochromic effect investigation

Solvent	f (ε, n)	f (ε, n) (revised)	ν_a-ν_f (cm^{-1}) 1,2-PhPOZ	1,2-PhPTZ	1,2-PhAc	1,2-PhPTZO	1,2-PhPTZOO
Cyclohexane	0.001	-0.002	1637.722	3285.129	1894.284	851.3273	968.773
n-hexane	0.001	-0.001	1646.316	3362.936	1823.362	851.3273	890.529
Toluene	0.013	0.013	1809.651	3464.909	1954.064	1057.027	1174.168
Chlorobenzene	-	0.143	2107.426	3552.849	2059.354	1129.484	1235.178
Chloroform	0.148	0.150	2262.82	3739.36	2367.424	1280.236	1471.158
Ethyl acetate	0.142	0.199	2236.257	3661.116	2162.372	1294.41	1397.516
THF	0.207	0.210	2335.669	3836.575	2448.362	1431.046	1904.762
Dichloromethane	0.148	0.217	2274.552	3620.961	2299.43	1351.92	1471.158
DMF	0.275	0.274	2738.152	4045.629	2702.15	1502.347	2046.23
Acetone	0.284	0.284	2738.152	4009.516	2716.833	2127.347	2128.096
Acetonitrile	0.305	0.304	2798.079	4188.199	2783.088	2012.822	2210.433
